# Expanding Color Control of Anodically Coloring Electrochromes Based on Electron-Rich 1,4-Dihydropyrrolo[3,2-*b*]pyrroles

**DOI:** 10.1021/acsaom.4c00197

**Published:** 2024-06-12

**Authors:** Allison M. Hawks, Lillian M. Daniel, Valentino S. Sorto, Julia Mauro, Perry Skiouris, Graham S. Collier

**Affiliations:** † Department of Chemistry and Biochemistry, Kennesaw State University, Kennesaw, Georgia 30144, United States; ‡ School of Polymer Science and Engineering, University of Southern Mississippi, Hattiesburg, Mississippi 39406, United States

**Keywords:** Pyrrolopyrroles, Electrochromism, Color Control, High Contrast, Absorption, Chromophores

## Abstract

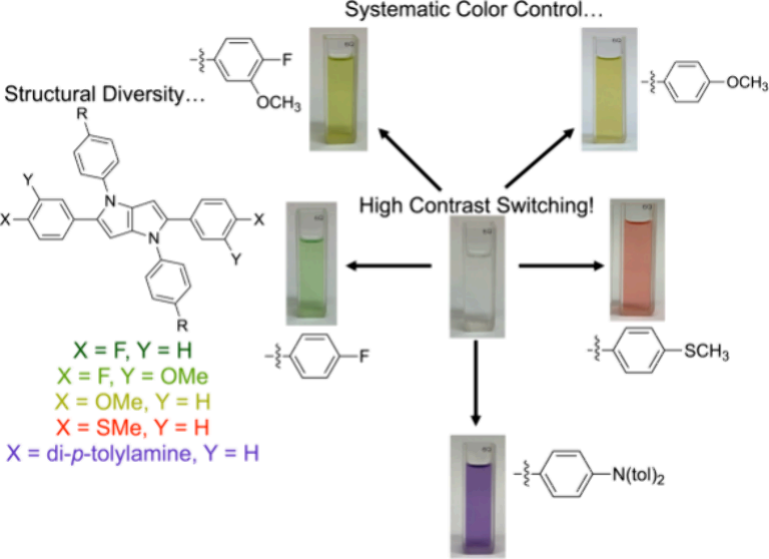

Anodically coloring electrochromes have received attention in recent years as high-contrast alternatives to cathodically coloring electrochromes due to their superior optical contrast during electrochemical switching. While current systems represent significant progress for organic electrochromics, it is necessary to expand the structural diversity of these materials while simultaneously reducing the hazards associated with synthetic protocols. With these considerations in mind, a family of 1,4-dihydropyrrolo[3,2-*b*]pyrrole (DHPP) chromophores with varying functionalities along the 2,5-axis was envisioned to accomplish these goals. After predicting different absorbance traits as oxidized molecules with time-dependent density functional theory, DHPP chromophores with varying peripheral functionalities were synthesized in a single aerobic synthetic step via an iron-catalyzed multicomponent reaction and characterized as high-contrast chromophores. In solution, the DHPP chromophores absorb in the ultraviolet region of the electromagnetic spectrum, resulting in color-neutral L*a*b* color coordinates of ∼100, 0, 0. Upon chemical oxidation, each molecule transitions to absorb at various points across the visible spectrum based on the extent of electron-donating ability and can display five distinct colors. Importantly, the chromophores are redox-active and display switching capabilities with an applied electrochemical potential. In conjunction with building fundamental insights into molecular design of DHPP chromophores, the results and synthetic simplicity of DHPPs make them compelling materials for color-controlled high-contrast electrochromes.

## Introduction

Electrochromic materials based on organic molecules and polymers have many potential applications,^1,2^ especially as multifunctional energy storage/conversion/saving devices,^3^ but suffer from drawbacks such as residual absorbances that minimize attainable contrasts between oxidation states. This is most prevalent within the class of electrochromic polymers known as cathodically coloring electrochromes (CCEs) that absorb within the visible region of the electromagnetic spectrum (EMS) as neutral species and transition to absorbing in the infrared region upon oxidation.^4−6^ This residual absorbance is emphasized in many high-contrast CCE polymers based on 3,4-(alkylene)dioxythiophenes (DOTs)^7^ and other structures.^8^ To overcome this residual absorbance, anodically coloring electrochromes (ACEs) have been developed where molecules absorb in the UV region as neutral molecules and transition to the visible region upon oxidation.^9−12^ By utilizing this approach, ACE molecules achieve true color neutrality in their neutral state with L*a*b* color coordinates of 100, 0, 0 and display systematic color control upon oxidation with maximum optical contrast.

While high-contrast materials are attainable, the structural design space for ACE molecules and polymers is quite sparse, and each set of materials comes with its own drawbacks. For example, ACE materials based on triarylamines^13^ or poly(amine-amides)^14^ can display high oxidation potentials, possess limited color control, or have poor device bistability. These materials also require numerous synthetic steps to prepare the corresponding monomers. Alkylenedioxypyrroles (DOPs)^15^ are also known to be high-contrast electrochromic materials, but their synthesis is relegated to electropolymerization^16^ or a stubborn dehalogenation polycondensation reaction.^17^ More recently, the Reynolds group reported a series of phenylene-functionalized dioxythiophenes that accomplish high-contrast electrochromism and color control of the radical cation.^9,11,18^ However, these molecules typically require multiple synthetic steps and use Stille cross-coupling reactions that produce stoichiometric amounts of toxic waste. The combined drawbacks across each class of materials motivate discovering new scaffolds that reduce the synthetic complexity, eliminate the use of toxic reagents, and accomplish this without sacrificing the color control properties of ACE chromophores.

**Figure 1:**
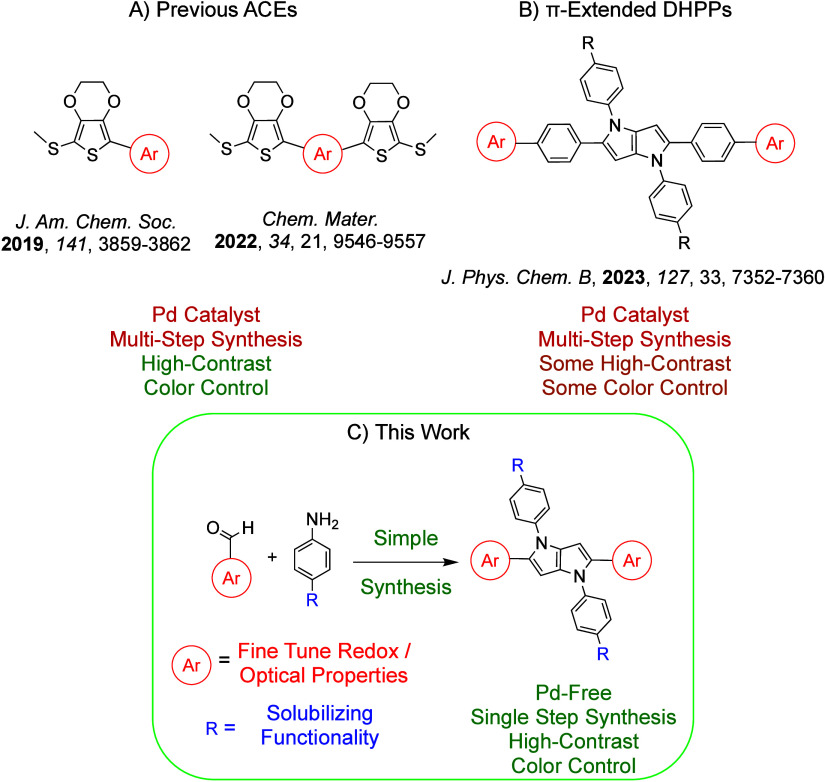
Representative structures used to achieve high-contrast ACE systems.

Recently, our group has been exploring the viability of utilizing 1,4-dihydropyrrolo[3,2-*b*]pyrroles (DHPPs) as synthetically simple monomers that participate in efficient polymerizations to yield simple yet tailorable conjugated polymers.^19^ We hypothesized that DHPPs would be a useful scaffold to accomplish this goal based on the work by the Gryko group, who developed a one-step multicomponent reaction to attain DHPPs with properties such as high fluorescence quantum yields and violet, blue, and green fluorescence.^20−23^ Through our efforts, we have shown that DHPPs reduce the synthetic complexity commonly associated with conjugated polymers,^24^ may be designed with structural handles that impart degradability/recyclability,^25^ and display multicolored electrochromism based on the choice of monomeric or molecular coupling partners.^19,26^ Upon closer examination of structure–property relationships that dictate optoelectronic properties, we showed that with diminishing push–pull nature of electron-rich DHPP chromophores, the absorbance of neutral molecules shifts toward the UV portion of the EMS. Upon oxidation, the absorbance of the radical cation species shifts to the visible, and the positioning and shape of the absorbance profile are dependent on peripheral functionalities. Converting absorbance spectra to L*a*b* color coordinates revealed that two of the molecules possess coordinates of 100, −1, 3, corresponding to highly color-neutral molecules. Encouraged by these results, and as alluded to in Figure 1, we hypothesized that a family of synthetically simple, high-contrast, and color-controlled ACE molecules based on DHPPs was possible. Accomplishing this goal would ultimately eliminate the need for multiple synthetic steps to attain high-contrast ACE materials while simultaneously expanding color control capabilities of DHPP-based electrochromes.

Time-dependent density functional theory (TD-DFT) is a powerful tool used for understanding structure–property relationships of optoelectronic materials.^27^ As such, TD-DFT has been essential in the development of chromophores and polymers that find utility in organic photovoltaics (OPVs),^28−30^ dye-sensitized solar cells (DSSCs),^31,32^ and electrochromics.^33^ However, TD-DFT calculations are usually used as supplemental data to support experimental observations. More recently, there is growing interest in utilizing computation to identify potential synthetic targets with desired properties.^34−36^ For example, TD-DFT has been used to predict the behavior of radical cations based on electronic and steric contributions,^9,11^ isomeric effects on optical properties of electrochromic polymers,^37^ and elastic constants of crystalline materials.^38^ The close agreement between experiment and calculations enables a streamlined approach for designing next-generation optoelectronic materials with reduced waste production and worker-hours in the lab. With these considerations in mind, expanding theory-driven projects will be impactful for the continued development of ACE molecules and polymers and motivates our research efforts.

Herein we report the design, synthesis, and characterization of a family of DHPP chromophores that display high-contrast electrochromism with systematic color control with low synthetic complexity. A theory-guided approach was exploited to predict changes in absorbance properties of neutral and oxidized DHPPs based on peripheral functionalization. Identification of chromophores that are predicted to absorb in the UV portion of the EMS but have different radical cation absorbance spectra ultimately guided synthetic efforts to attain five DHPP chromophores with varying peripheral functionalities via a one-step Fe-catalyzed multicomponent reaction. Electrochemical characterization via cyclic voltammetry (CV) and differential pulse voltammetry (DPV) revealed that increasing the electron-donating nature of the peripheral functional groups lowers the onset of oxidation without sacrificing wide optical bandgaps (∼3.0 eV) that facilitate absorbance in UV region of the EMS. Upon oxidation, absorbances transition to the visible region, where the positioning and shape of the absorbance profile are influenced by the electron-donating or -withdrawing nature of peripheral functional groups. The molecules are highly color-neutral as neutral solutions with L*a*b* color coordinates ∼100, 0, 0 and transition to distinctly different and vibrant colors ranging from green to purple in the oxidized state. Ultimately, this study reports a strategy to attain synthetically simple, high-contrast electrochromes with systematic color control across three color quadrants. Results from this study represent an expansion in the applicability of DHPP chromophores and further reinforces their utility as functional conjugated scaffolds while simultaneously expanding the structural diversity of ACE molecules.

## Results and Discussion

**Figure 2:**
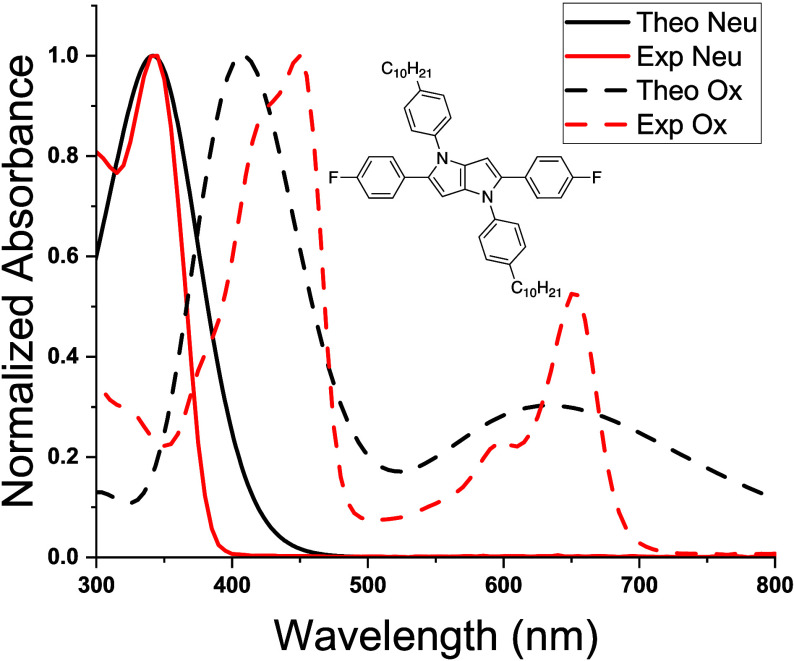
Comparison of calculated and experimental UV–vis absorbance spectra of 4-FDHPP that shows the level of theory used for TD-DFT is reliable and accurate for predicting the neutral and oxidized UV–vis absorbance spectra of DHPP chromophores.

When envisioning strategies to understand how peripheral substituents of DHPP molecules dictate the position and shape of the resulting radical cation, incorporating substituents that are electron-withdrawing, electron-donating, or a combination of the two seems logical. This is motivated by the success of influencing the optoelectronic properties of π-extended DHPPs reported by our group^26^ and studies of dioxythiophene-based ACE molecules.^9,12^ Our initial efforts involved establishing agreement between theory and experiment by modeling and characterizing a series of fluorinated DHPPs. A thorough discussion of these efforts is provided in the Supporting Information and is reported within Figures S15–S19 and Tables S5 and S6. In short, the close agreement between theory and experiment shown in Figure 2 validated the level of theory (B3LYP 6-31G*) and supports screening additional chromophores.

**Figure 3:**
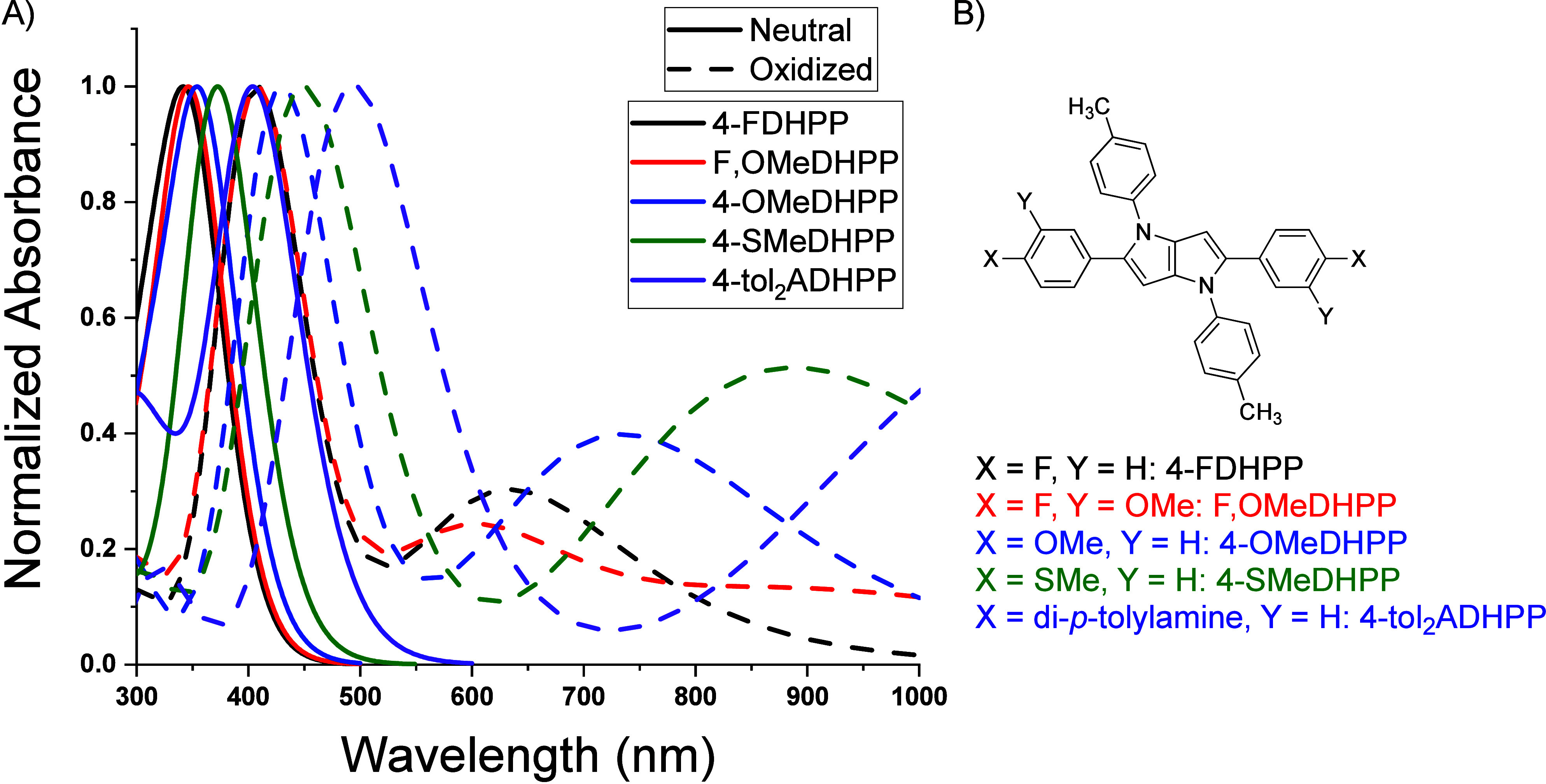
(A) Calculated neutral and oxidized absorbance spectra and (B) the accompanying DHPP structures screened for potential color control. The R groups were reduced to CH_3_ groups to simplify structural input into TD-DFT calculations. The neutral spectra are shown as the solid lines while the oxidized are represented as dashed lines.

Five DHPPs with varying electron-donating capabilities were designed based on commercial availability of starting material and modeled with TD-DFT. As shown in Figure 3, each of the DHPP molecules was predicted to absorb mostly within the UV region of the EMS and achieves the first requirement for anodically coloring materials. After modeling the singly oxidized state for each molecule, the absorbance profiles resemble the dual-band absorbances observed for dioxythiophene-based ACE chromophores that are indicative of SOMO → LUMO transitions.^9−12^ The SOMO-α → LUMO-α (high-energy absorbance) transitions are predicted to appear between 400 and 500 nm, while the intensities (i.e., oscillator strengths) and positions of the SOMO-β → LUMO-β transitions (low-energy absorbance) are more drastically influenced by the electronic character of peripheral substitution. Figure 3 emphasizes the predicted dependence of the SOMO-β → LUMO-β absorbance on the choice of functionality that will lead to color control of DHPP electrochromes. Specifically, as the electron-donating ability is increased, the SOMO-β → LUMO-β transition increasingly red-shifts into the NIR. In sum, these calculations support the notion that simple alterations to peripheral substituents will enable systematic color control and motivate continued synthesis and elucidation of structure–property relationships of DHPP electrochromes.

**Scheme 1:**
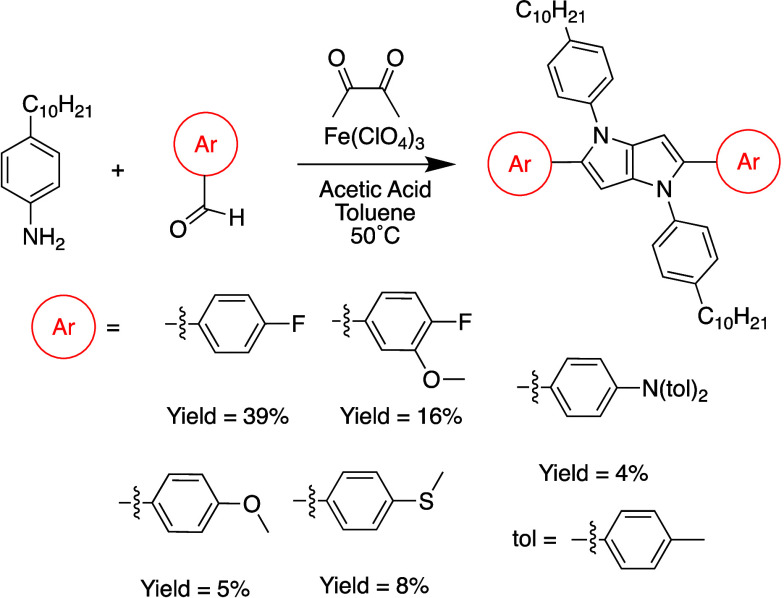
Synthesis of DHPP Molecules with Varying Functional Groups Using the Fe-Catalyzed Multicomponent Reaction Conditions

Motivated by calculations predicting that UV–vis absorbance transitions of the neutral molecules should be positioned within the UV region of the EMS, the five DHPP molecules from Figure 3 were synthesized using the Fe-catalyzed multicomponent reaction developed by Gryko and co-workers^23^ and adopted by our group in our previous studies (Scheme 1).^19,25,26^ The diagnostic DHPP peak at ∼6.5 ppm in the ^1^H NMR spectra that corresponds to the two protons on the fused pyrrolopyrrole ring is present for each molecule and supports successful formation of the DHPP chromophores. This is illustrated in the ^1^H NMR spectra reported in the Supporting Information (for example, Figure S1). Additionally, the multiplicities in the ^13^C NMR spectra for DHPPs with fluorine substituents were analyzed for C–F coupling. The results are reported in Tables S1–S4, and analyses of the C–F coupling constants are consistent with previous reports.^39^ Elemental analysis also was used to confirm the purity of the newly synthesized DHPPs, and the theoretical values matched the experimental ones with a high level of accuracy. Overall, the robust protocol for synthesizing DHPPs yields a structurally diverse family of chromophores suited for elucidating structure–property relationships that progress the development of ACE molecules.

**Figure 4:**
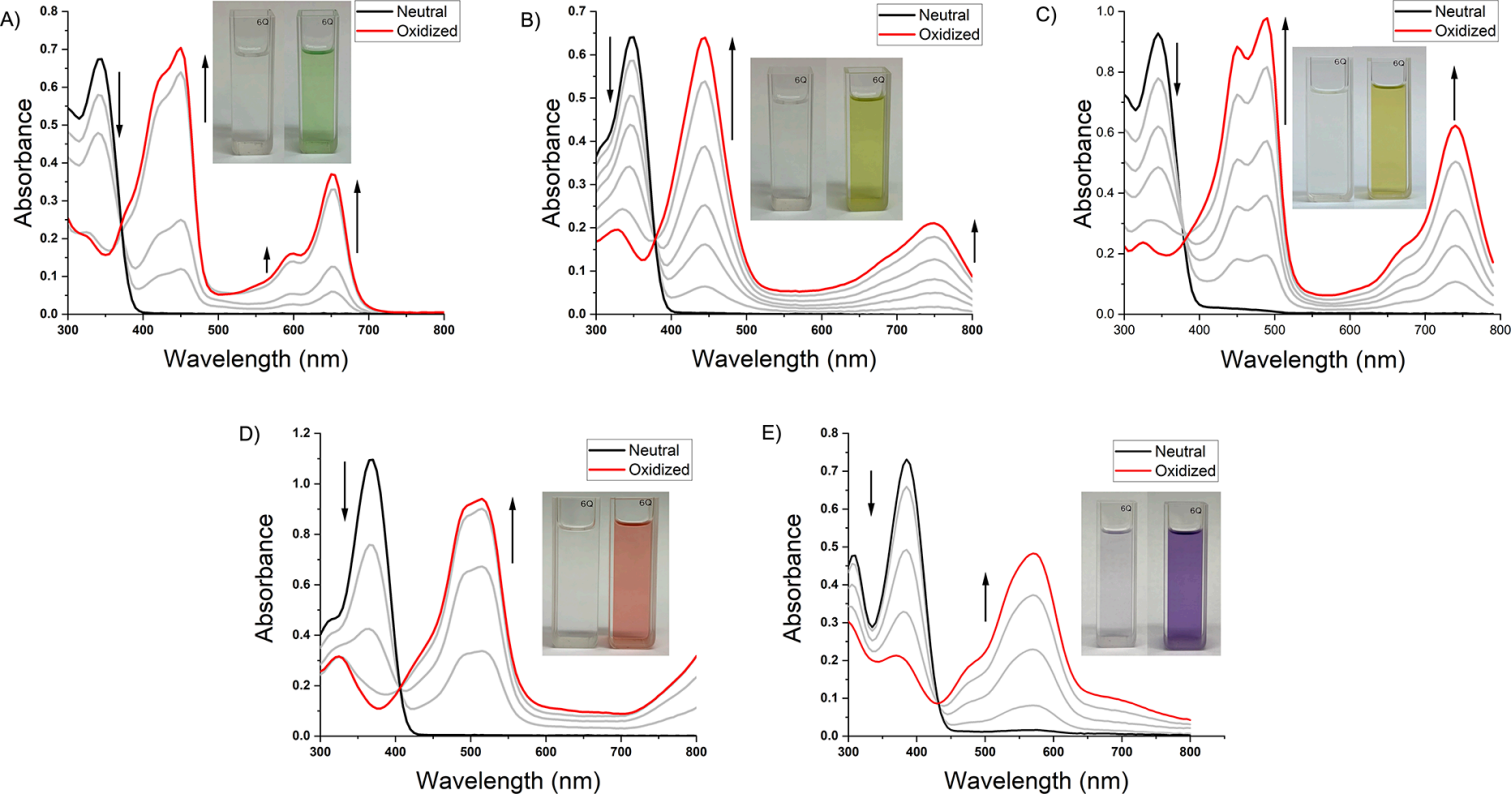
UV–vis solution oxidation studies of (A) 4-FDHPP, (B) F,OMeDHPP, (C) 4-OMeDHPP, (D) 4-SMeDHPP, and (E) 4-tol_2_ADHPP using 0.06 mg/mL Fe(ClO_4_)_3_·*x*H_2_O in ethyl acetate as the dopant. These results demonstrate the ability to manipulate the positioning and shape of the radical cation absorbance by varying the electronic character.

**Figure 5:**
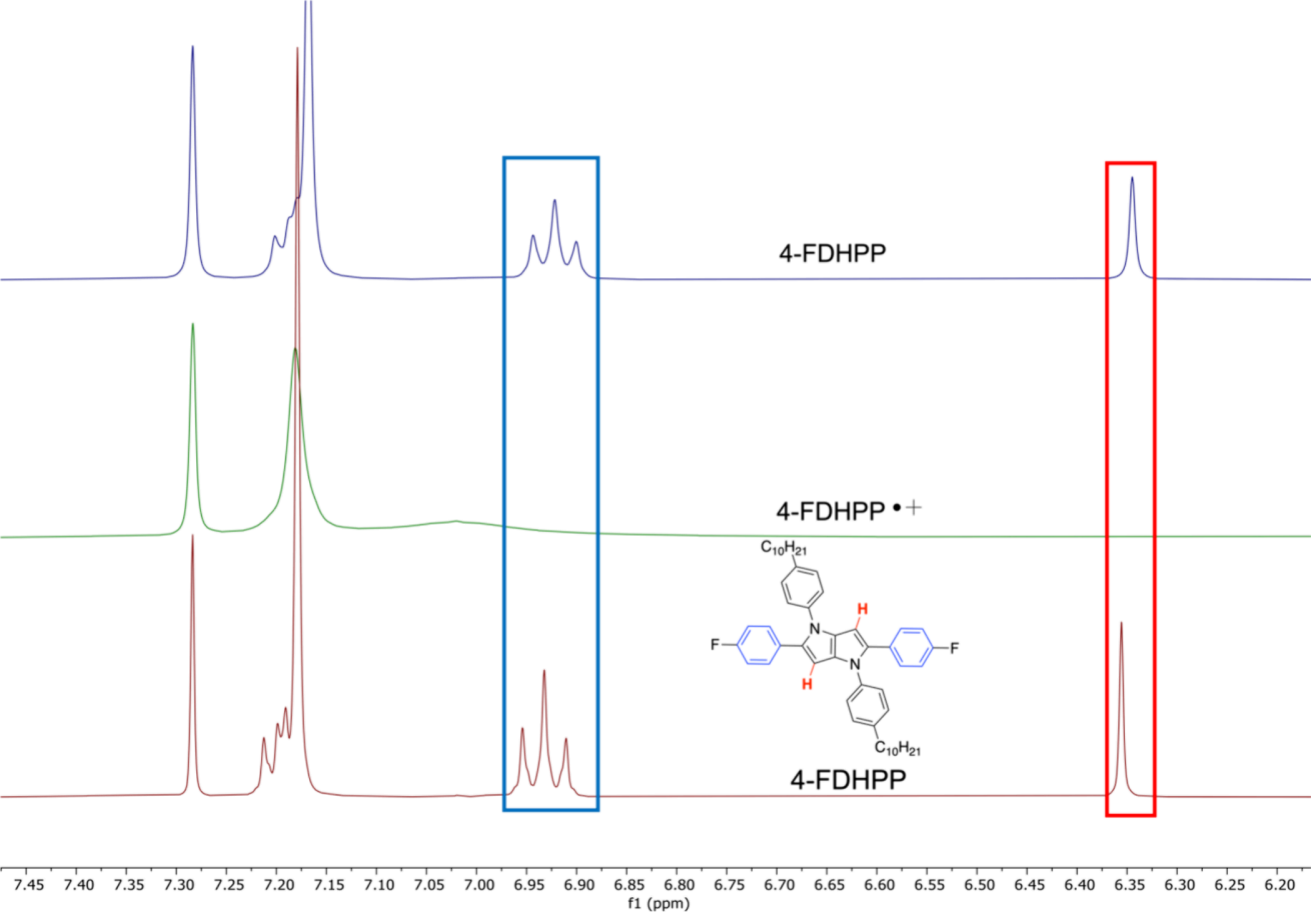
^1^H NMR spectra of 4-FDHPP as a pristine molecule (red), after exposure to Fe(ClO_4_)_3_·*x*H_2_O to form the radical cation 4-FDHPP*^+^ (green), and after reduction back to 4-FDHPP with hydrazine (blue).

The predicted and experimental optical properties were compared and, as shown in Figure S20, all of the molecules absorb in the UV region, and the theoretical and experimental spectra are in adequate agreement. In order to understand changes in optical properties with increasing levels of oxidation, solution oxidation studies were performed by titrating DHPP chromophore solutions with a chemical oxidant and monitoring the change in absorbance. As shown in Figure 4, the absorbance corresponding to the π–π* transition (black trace) for each molecule diminishes with increasing oxidant concentration while transitions evolve across the visible region of the EMS. F,OMeDHPP has a SOMO-α → LUMO-α similar to that of 4-FDHPP with an λ_max_^α^ of 445 and 450 nm, respectively. On the other hand, 4-OMeDHPP has a red-shifted λ_max_^α^ of 490 nm with characteristics of vibronic fine structure that may be attributed to radical dimerization.^10,40−43^ Increasing the electron-donating strength for 4-SMeDHPP results in a further red shift to a λ_max_^α^ of 515 nm, and 4-tol_2_ADHPP demonstrates an even more pronounced red shift with a λ_max_^α^ of 565 nm. The SOMO-β → LUMO-β absorbance differs most significantly in the peak shape, with 4-FDHPP having a well-defined peak at 650 nm with shouldering around ∼600 nm, while F,OMeDHPP has a relatively less distinct peak with a λ_max_^β^ at 750 nm. Alternatively, 4-OMeDHPP has a well-defined peak at 740 nm with shouldering around ∼675 nm. Furthermore, the increased electron-donating capabilities of 4-SMeDHPP and 4-tol_2_ADHPP yield a more pronounced red shift of the SOMO-β → LUMO-β absorbance, resulting in a λ_max_^β^ > 800 nm for both molecules. All of these results are tabulated in Table 1. The differences in the UV–vis absorbances of the radical cations are consistent with previous studies investigating the effects of electronic influence on positioning of radical cation absorbances. For example, Reynolds and co-workers found that manipulating the electron-donating or -withdrawing character of benzene units results in shifts in the absorbance of the radical cation.^9,18^ Additionally, our group showed that choice of peripheral functionality on biphenyl-functionalized DHPPs changed the absorbance profile of neutral and oxidized DHPP chromophores.^26^ Importantly, the neutral absorbance can be recovered with addition of hydrazine, which demonstrates the reversibility of the redox process and is encouraging for repeated switching experiments when incorporated into a device architecture. The reversibility was further studied with ^1^H NMR to confirm that the DHPP chromophore was recovered after the doping/dedoping process. As shown in Figure 5, the diagnostic pyrrolopyrrole proton at ∼6.37 ppm for 4-FDHPP disappears after the addition of the Fe oxidant and formation of the radical cation (green trace). Furthermore, the protons associated the benzene rings along the 2,5-axis also are absent, while protons on the 1,4-benzene rings display line broadening, which is a common phenomenon when studying doped molecules via NMR.^44,45^ The different trends observed for the benzene rings are attributed to the varying electronic communication along the conjugation pathways^46,47^ and the 2,5-axis contributing to the redox processes more readily than the 1,4-axis. Following the addition of hydrazine, the radical cation is reduced back to the parent DHPP structure, as evidenced by the reappearance of the protons distributed across the 2,5-axis of the molecule (blue trace), and this confirms the absence of undesired side reactions during the doping protocols. Overall, the solution oxidation studies of these five molecules demonstrate the ability to manipulate the absorbance of the radical cations of DHPP molecules while maintaining a neutral absorbance in the UV region of the EMS, and make them suited for high-contrast electrochromes.

**Table 1:** Optical data for all ACE molecules including the λ_max_ and color coordinates for the neutral and oxidized species

		λ_max_^ox^ (nm)		L*a*b* color coordinates	
chromophore	λ_max_^neu^ (nm)	SOMO-α	SOMO-β	neu.	ox.
4-FDHPP	345	450	650	100, 0, 0	91, −27, 38
F,OMeDHPP	350	445	750	100, 0, 0	94, −11, 46
4-OMeDHPP	345	490	740	100, 0, 2	89, −4, 68
4-SMeDHPP	370	515	>800	100, 0, 0	73, 20, −27
4-tol_2_ADHPP	385	565	N/A	99, −1, 4	95, −12, 23

**Figure 6:**
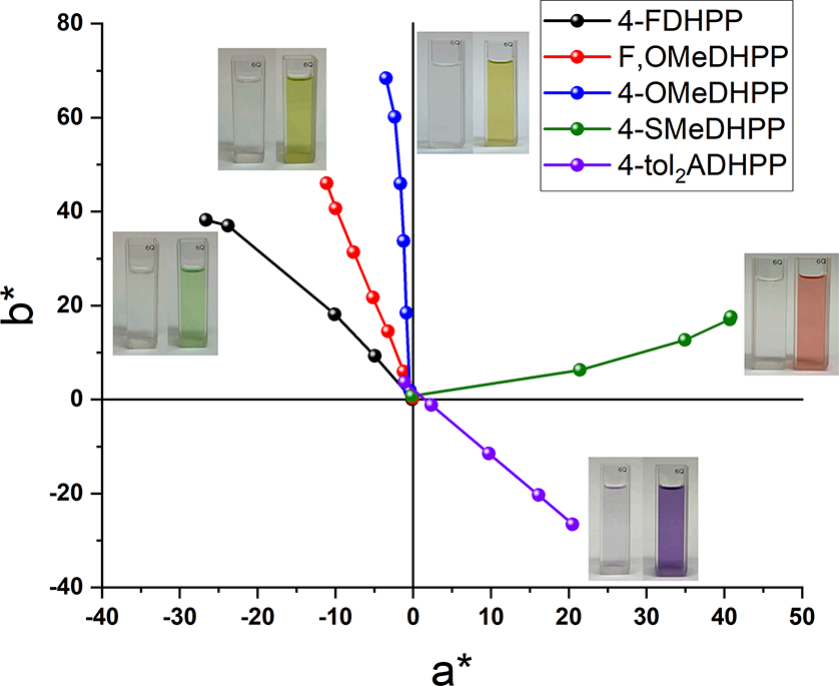
Color coordinate diagram of 4-FDHPP (black), F,OMeDHPP (red), 4-OMeDHPP (blue), 4-SMeDHPP (green), and 4-tol_2_ADHPP (purple) obtained from the solution oxidation studies. All five start at the origin as transmissive, neutral solutions and upon oxidation track away from the origin to produce five distinct colors.

The changes in absorbance led to distinct color changes of the solutions during oxidation experiments. The observed color changes enabled calculation of color coordinates using the absorbance data collected during solution oxidation studies. Colorimetric analysis based on the Commission Internationale de l’Eclairage 1976 L*a*b* color space was used at a D50 illuminant as a 2° observer to quantify the color of these DHPP chromophores.^48^ All five DHPP molecules begin in their neutral state at the origin with L*a*b* values of ∼100, 0, and 0 (Figure 6 and Table 1), and the transmissive solutions are emphasized by the photographs in the insets of Figure 6. Color neutrality is defined by a* and b* values falling within the range ±10, while L* values of ∼100 correspond to transmissive samples.^49^ As the Fe oxidant is added, the color tracks away from the graph’s origin toward the color quadrant that corresponds to the absorbance profile of the radical cation. Excitingly, as shown in Figure 6 and tabulated in Table 1, the color data for this family of DHPP molecules appear across three different quadrants of the color coordinate diagram. This expansion of color control is important because our first report of high-contrast DHPP electrochromes was restricted to a single color quadrant.^26^ The electron-withdrawing 4-FDHPP is green, and as the positioning and strength of the electron-donating functionality changes, the color coordinates shift toward the yellow region, to the red region, and finally within the purple region. These results are in excellent agreement with UV–vis absorbance data that show a red shift in the low-energy transition of the radical cation into the IR as well as the colored solutions in the photographs in Figure 6. Overall, the colorimetry data confirm the hypothesis that making small changes to the periphery of DHPP chromophores enables systematic color control of high-contrast DHPP-based ACE molecules.

**Figure 7:**
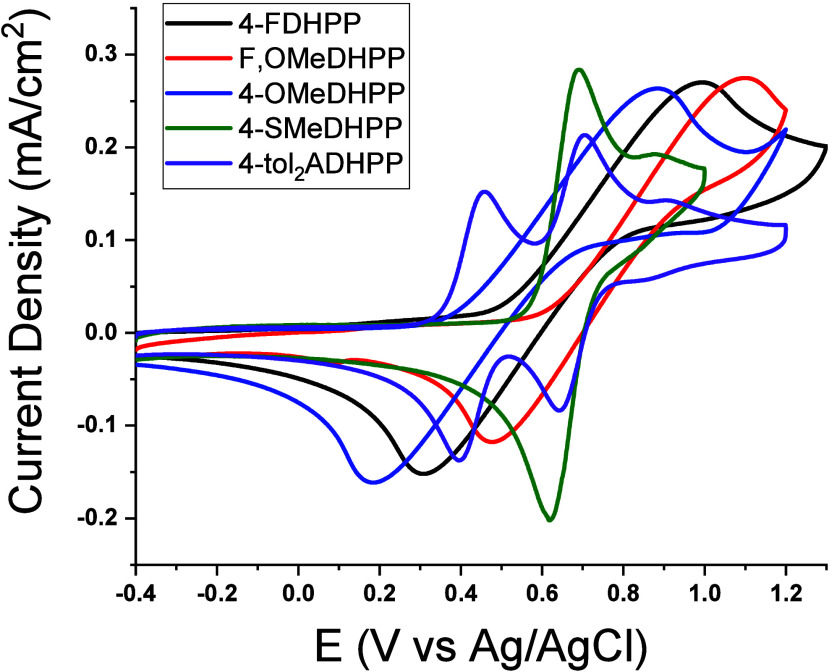
CV traces of 4-FDHPP, F,OMeDHPP, 4-OMeDHPP, 4-SMeDHPP, and 4-tol_2_ADHPP using a Ag/AgCl reference electrode and a 0.5 M TBAPF_6_/DCM supporting electrolyte. This figure demonstrates the ability to manipulate the redox properties of DHPPs depending on the functionalization at the *para*-position of the benzene units.

While the optical properties were found to be appropriate for a new class of ACE molecules, redox properties required for electrochemical switching need to be elucidated as well. DHPPs are known to be abundantly electron-rich conjugated systems,^23^ and electrochemical measurements are warranted due to the reported redox activity of numerous DHPPs.^46,50−58^ Redox properties for this study were measured via CV and DPV to understand structural influences on the properties such as the onset of oxidation and reversibility of the DHPP molecules. The ability to manipulate the onset of oxidation of a DHPP through variation of electron-withdrawing or/and -donating groups is shown in Figure 7 and Table 2. Specifically, as electron-donating ability is increased, the onset of oxidation is decreased. Importantly, for electrochemical switching, a reduction accompanies the oxidation for all five DHPPs. Furthermore, Figure S21 illustrates the similarities in the energy band gaps for all the DHPP molecules and the calculated band gaps (∼3.0 eV) agree with optical measurements where the molecules absorb in the UV region. If the electrochemical window is expanded to 2 V, dications form that lead to the degradation of the molecules (Figure S18). Overall, these results establish these DHPPs as electroactive molecules and motivate the continued investigation into color changes with an electrochemical potential for eventual use in electrochromic applications.

**Table 2:** Electronic Properties of DHPPs with Various Substituents

chromophore	*E*_ox_^onset^ (V)	*E*_HOMO_ (eV)^a^	*E*_LUMO_ (eV)^b^	*E*_gap_ (eV)^c^
4-FDHPP	0.42	–5.5	–2.4	3.1
F,OMeDHPP	0.49	–5.6	–2.6	3.0
4-OMeDHPP	0.26	–5.4	–2.3	3.1
4-SMeDHPP	0.51	–5.6	–2.7	2.9
4-tol_2_ADHPP	0.28	–5.4	–2.6	2.8

a Calculated given *E*_HOMO_ = −(*E*_ox_^onset^ + 5.12 eV).

b Calculated from the absorbance onset given *E*_LUMO_ = 1240/*λ*_onset_ + *E*_HOMO_.

c Calculated from (LUMO – HOMO). All equations were adopted from Cardona and co-workers.^59^

**Figure 8:**
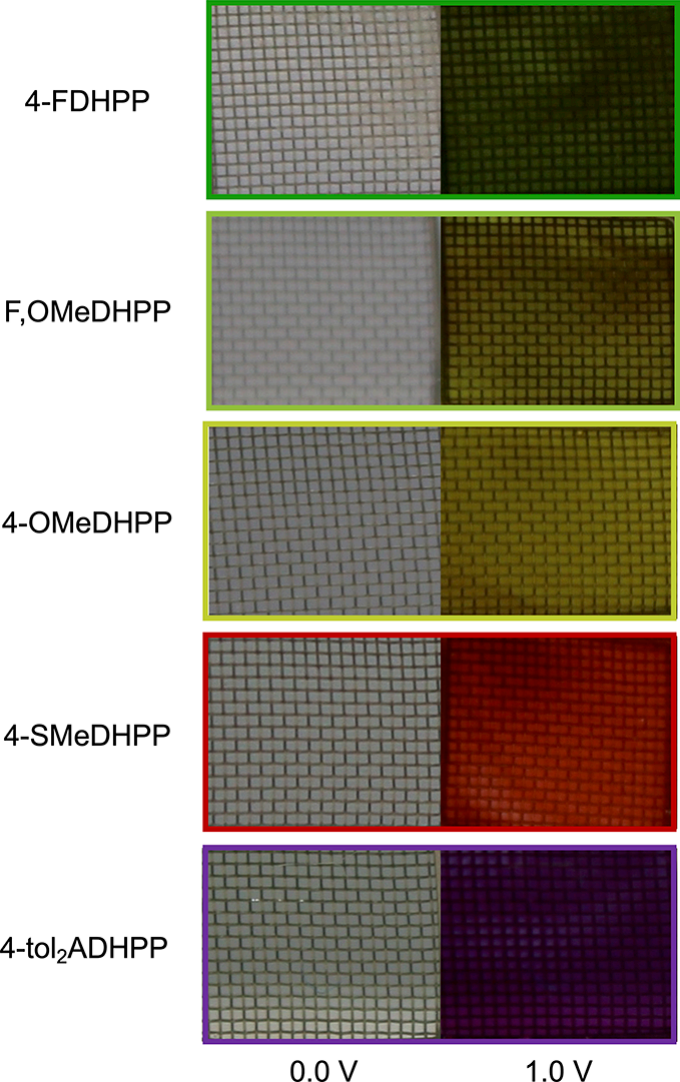
Electrochemical switching experiments of the color-controlled high-contrast DHPP chromophores, including 4-FDHPP, F,OMeDHPP, 4-OMeDHPP, 4-SMeDHPP, and 4-tol_2_ADHPP.

After elucidating structure–property relationships that govern color control of DHPP ACE molecules, efforts involved demonstrating that these color changes occur under electrochemical switching conditions. Here, the molecules were dissolved in anhydrous DCM and placed into a SEC-C thin-layer quartz glass spectroelectrochemical cell with a platinum gauze working electrode. The solutions were held at 0.0 V and photographed to emphasize color neutrality before applying a 1.0 V potential until full color saturation was achieved. As shown in the photographs in Figure 8, each molecule starts as a transmissive solution in its neutral state and shifts to a vibrant color upon application of an electrochemical potential. Attempts to study these materials as thin films were futile because the chromophores are soluble in many organic solvents that are used in electrochemical measurements (i.e., acetonitrile, propylene carbonate, etc.). These solubility constraints necessitate future design strategies that render DHPP films solvent-resistant, either through polymerization or chemical cross-linking, such that electrochromic properties in the solid state may be studied. Ultimately, the high contrast between neutral and oxidized states with an electrochemical potential paves the way for these DHPPs to be incorporated into electrochromic devices.

## Conclusion

Organic electrochromes offer the ability to systematically control color properties through the careful choice of structural motifs, but there is a need to improve the overall optical contrast during electrochemical switching protocols. In a broader context for the field of conjugated materials, reducing the synthetic complexity of materials with tailorable properties is highly desired. This study addresses both of these goals by exploiting the simple synthesis of DHPPs to access a family of highly tailorable ACE molecules in a single step. Attaining these molecules subsequently enables understanding fundamental structure–property relationships of DHPP chromophores in their neutral and oxidized states. A theory-guided approach is utilized to guide synthetic efforts that ultimately create the first examples of high-contrast, color-controlled DHPP electrochromes. TD-DFT calculations provide precedent that subtle changes in electronic character along the 2,5-axis of DHPP chromophores influence the positioning of the radical cation absorbance while neutral molecules absorb in the UV portion of the EMS. Subsequently, a structurally diverse family of DHPP chromophores was synthesized via an Fe-catalyzed multicomponent reaction and isolated with vacuum filtration. Analysis of optoelectronic properties of the DHPP chromophores revealed a relationship between electrochemical and optical properties and peripheral functionality and corroborated TD-DFT calculations. Specifically, with increasing electron-donating ability of peripheral functionalities, the absorbance of the radical cation species red-shifts across the visible spectrum while also having a lower onset of oxidation. Results from optical measurements are quantified in the L*a*b* color space where neutral solutions are highly color-neutral with L*a*b* ∼ 100, 0, 0 and oxidized solutions occupy three distinct color quadrants from green to purple. In sum, results from these efforts provide useful fundamental insights into design–structure–property relationships of DHPP chromophores and inspire the investigation into additional design motifs that expand the color palette of DHPP electrochromes. This work also represents a simplification of the preparation of electrochromic materials. Ultimately, efforts described in this article reveal new design motifs for high-contrast color-controlled DHPP-based electrochromes and pave the way for incorporating DHPPs into redox devices.

## Materials and Methods

Comprehensive details used for experiments are compiled in the Supporting Information. TD-DFT calculations with the B3LYP/6-31G* functional/basis set were performed using Gaussian 16^60^ to elucidate the optical properties of the DHPP molecules that are synthetic targets. All materials were purchased from commercial sources and used as received unless otherwise stated. ^1^H and ^13^C NMR spectra were collected on a Bruker Avance III HD 400 MHz NMR spectrometer with nominal concentrations of 5 mg/mL in CDCl_3_. Peaks are referenced to the residual CHCl_3_ peaks (^1^H, δ = 7.26 ppm; ^13^C, δ = 77.23 ppm). Optical absorbance spectra were acquired using a Varian Cary 60 Scan single-beam UV–vis–NIR spectrophotometer scanning from 300 to 800 nm. Cyclic voltammetry (CV) and differential pulse voltammetry (DPV) measurements were performed with a CH Instruments electrochemical workstation (CHI660D) using a glassy carbon electrode as the working electrode, a Ag/AgCl reference electrode (calibrated versus the Fc/Fc^+^ redox couple, *E*_1/2_ = 40 mV), and a Pt wire as the counter electrode. Photography was performed in a light booth designed to exclude outside light with controllable LED lighting above providing illumination. A Canon Rebel T7 camera with an 18–55 mm lens was used to capture images.

## Supporting Information Available

Detailed synthetic protocols, ^1^H and ^13^C NMR spectra, supporting spectroscopic and color analysis, and electrochemical and electronic data (PDF)

## References

[ref1] Gu C.; Jia A. B.; Zhang Y. M.; Zhang S. X. A. Emerging Electrochromic Materials and Devices for Future Displays. Chem. Rev. 2022, 122 (18), 14679–14721. 10.1021/acs.chemrev.1c01055. 35980039 PMC9523732

[ref2] Li X.; Perera K.; He J.; Gumyusenge A.; Mei J. Solution-Processable Electrochromic Materials and Devices: Roadblocks and Strategies Towards Large-Scale Applications. J. Mater. Chem. C 2019, 7 (41), 12761–12789. 10.1039/C9TC02861G.

[ref3] Kandpal S.; Ghosh T.; Rani C.; Chaudhary A.; Park J.; Lee P. S.; Kumar R. Multifunctional Electrochromic Devices for Energy Applications. ACS Energy Lett. 2023, 8 (4), 1870–1886. 10.1021/acsenergylett.3c00159.

[ref4] Beaujuge P. M.; Reynolds J. R. Color Control in π-Conjugated Organic Polymers for Use in Electrochromic Devices. Chem. Rev. 2010, 110 (1), 268–320. 10.1021/cr900129a. 20070115

[ref5] Madasamy K.; Velayutham D.; Suryanarayanan V.; Kathiresan M.; Ho K.-C. Viologen-Based Electrochromic Materials and Devices. J. Mater. Chem. C 2019, 7 (16), 4622–4637. 10.1039/C9TC00416E.

[ref6] Zhou D.; Xie D.; Xia X.; Wang X.; Gu C.; Tu J. All-Solid-State Electrochromic Devices Based on WO_3_||NiO Films: Material Developments and Future Applications. Sci. China: Chem. 2017, 60, 3–12. 10.1007/s11426-016-0279-3.

[ref7] Kumar A.; Welsh D. M.; Morvant M. C.; Piroux F.; Abboud K. A.; Reynolds J. R. Conducting Poly(3,4-Alkylenedioxythiophene) Derivatives as Fast Electrochromics with High-Contrast Ratios. Chem. Mater. 1998, 10 (3), 896–902. 10.1021/cm9706614.

[ref8] Neo W. T.; Ye Q.; Chua S. J.; Xu J. Conjugated Polymer-Based Electrochromics: Materials, Device Fabrication and Application Prospects. J. Mater. Chem. C 2016, 4 (31), 7364–7376. 10.1039/C6TC01150K.

[ref9] Christiansen D. T.; Tomlinson A. L.; Reynolds J. R. New Design Paradigm for Color Control in Anodically Coloring Electrochromic Molecules. J. Am. Chem. Soc. 2019, 141, 3859–3862. 10.1021/jacs.9b01507. 30794389

[ref10] Österholm A. M.; Nhon L.; Shen D. E.; Dejneka A. M.; Tomlinson A. L.; Reynolds J. R. Conquering Residual Light Absorption in the Transmissive States of Organic Electrochromic Materials. Mater. Horiz. 2022, 9 (1), 252–260. 10.1039/D1MH01136G. 34635899

[ref11] Nhon L.; Tennyson S. L.; Butt M. W.; Bacsa J.; Tomlinson L.; Reynolds J. R. Theory-Driven Spectral Control of Bis-EDOT Arylene Radical Cation Chromophores. Chem. Mater. 2022, 34 (21), 9546–9557. 10.1021/acs.chemmater.2c02054.

[ref12] Nhon L.; Wilkins R.; Reynolds J. R.; Tomlinson A. Guiding Synthetic Targets of Anodically Coloring Electrochromes through Density Functional Theory. J. Chem. Phys. 2021, 154, 054110 10.1063/5.0039511. 33557540

[ref13] Yen H. J.; Liou G. S. Solution-Processable Triarylamine-Based Electroactive High Performance Polymers for Anodically Electrochromic Applications. Polym. Chem. 2012, 3 (2), 255–264. 10.1039/C1PY00346A.

[ref14] Liou G. S.; Lin H. Y. Synthesis and Electrochemical Properties of Novel Aromatic Poly(Amine-Amide)s with Anodically Highly Stable Yellow and Blue Electrochromic Behaviors. Macromolecules 2009, 42 (1), 125–134. 10.1021/ma8021019.

[ref15] Walczak R. M.; Reynolds J. R. Poly(3,4-Alkylenedioxypyrroles): The PXDOPs as Versatile yet Underutilized Electroactive and Conducting Polymers. Adv. Mater. 2006, 18 (9), 1121–1131. 10.1002/adma.200502312.

[ref16] Schottland P.; Zong K.; Gaupp C. L.; Thompson B. C.; Thomas C. A.; Giurgiu I.; Hickman R.; Abboud K. A.; Reynolds J. R. Poly(3,4-Alkylenedioxypyrrole)s: Highly Stable Electronically Conducting and Electrochromic Polymers. Macromolecules 2000, 33 (19), 7051–7061. 10.1021/ma000490f.

[ref17] Arroyave F. A.; Reynolds J. R. Dioxypyrrole-Based Polymers via Dehalogenation Polycondensation Using Various Electrophilic Halogen Sources. Macromolecules 2012, 45 (15), 5842–5849. 10.1021/ma300684t.

[ref18] Wagner J. S.; Smith E.; Bacsa J.; Tomlinson A. L.; Reynolds J. R. Color Control in Bis-Ethylenedioxythiophene Phenylene Anodically Coloring Electrochromes. Chem. Mater. 2023, 35 (24), 10550–10563. 10.1021/acs.chemmater.3c02167.

[ref19] Bell K.-J. J.; Kisiel A. M.; Smith E.; Tomlinson A. L.; Collier G. S. Simple Synthesis of Conjugated Polymers Enabled via Pyrrolo[3,2-*b*]Pyrroles. Chem. Mater. 2022, 34, 8729–8739. 10.1021/acs.chemmater.2c01884.

[ref20] Krzeszewski M.; Gryko D.; Gryko D. T. The Tetraarylpyrrolo[3,2-*b*]Pyrroles - From Serendipitous Discovery to Promising Heterocyclic Optoelectronic Materials. Acc. Chem. Res. 2017, 50 (9), 2334–2345. 10.1021/acs.accounts.7b00275. 28795799

[ref21] Janiga A.; Glodkowska-Mrowka E.; Stoklosa T.; Gryko D. T. Synthesis and Optical Properties of Tetraaryl-1,4-Dihydropyrrolo[3,2-*b*]Pyrroles. Asian J. Org. Chem. 2013, 2 (5), 411–415. 10.1002/ajoc.201200201.

[ref22] Tasior M.; Koszarna B.; Young D. C.; Bernard B.; Jacquemin D.; Gryko D.; Gryko D. T. Fe(III)-Catalyzed Synthesis of Pyrrolo[3,2-*b*] Pyrroles: Formation of New Dyes and Photophysical Studies. Org. Chem. Front. 2019, 6 (16), 2939–2948. 10.1039/C9QO00675C.

[ref23] Tasior M.; Vakuliuk O.; Koga D.; Koszarna B.; Górski K.; Grzybowski M.; Kielesiński Ł.; Krzeszewski M.; Gryko D. T. Method for the Large-Scale Synthesis of Multifunctional 1,4-Dihydro-Pyrrolo[3,2-*b*]Pyrroles. J. Org. Chem. 2020, 85, 13529–13543. 10.1021/acs.joc.0c01665. 32907329 PMC7656515

[ref24] Bell K.-J. J.; Sabury S.; Phan V.; Wagner E. M.; Hawks A. M.; Bartlett K. A.; Collier G. S. Synthesis of 1,4-Dihhydropyrrolo[3,2-*b*]Pyrrole-Containing Donor-Acceptor Copolymers and Their Optoelectronic Properties. J. Polym. Sci. 2024, 10.1002/pol.20240093.

[ref25] Bartlett K. A.; Charland-Martin A.; Lawton J.; Tomlinson A. L.; Collier G. S. Azomethine-Containing Pyrrolo[3,2-*b*]Pyrrole Copolymers for Simple and Degradable Conjugated Polymers. Macromol. Rapid Commun. 2024, 45, 2300220 10.1002/marc.202300220. 37449343

[ref26] Hawks A. M.; Altman D.; Faddis R.; Wagner E. M.; Bell K. J.; Charland-Martin A.; Collier G. S. Relating Design and Optoelectronic Properties of 1,4-Dihydropyrrolo[3,2-*b*]Pyrroles Bearing Biphenyl Substituents. J. Phys. Chem. B 2023, 127 (33), 7352–7360. 10.1021/acs.jpcb.3c03061. 37561612 PMC10461294

[ref27] Adamo C.; Jacquemin D. The Calculations of Excited-State Properties with Time-Dependent Density Functional Theory. Chem. Soc. Rev. 2013, 42 (3), 845–856. 10.1039/C2CS35394F. 23117144

[ref28] Ku J.; Lansac Y.; Jang Y. H. Time-Dependent Density Functional Theory Study on Benzothiadiazole-Based Low-Band-Gap Fused-Ring Copolymers for Organic Solar Cell Applications. J. Phys. Chem. C 2011, 115 (43), 21508–21516. 10.1021/jp2062207.

[ref29] Berube N.; Gosselin V.; Gaudreau J.; Cote M. Designing Polymers for Photovoltaic Applications Using Ab Intio Calculations. J. Phys. Chem. C 2013, 117 (16), 7964–7972. 10.1021/jp309800f.

[ref30] Cui Y.; Li P.; Song C.; Zhang H. Terminal Modulation of D-π-A Small Molecule for Organic Photovoltaic Materials: A Theoretical Molecular Design. J. Phys. Chem. C 2016, 120 (51), 28939–28950. 10.1021/acs.jpcc.6b09927.

[ref31] Azaid A.; Raftani M.; Alaqarbeh M.; Kacimi R.; Abram T.; Khaddam Y.; Nebbach D.; Sbai A.; Lakhlifi T.; Bouachrine M. New Organic Dye-Sensitized Solar Cells Based on the D-A-π-A Structure for Efficient DSSCs: DFT/TD-DFT Investigations. RSC Adv. 2022, 12 (47), 30626–30638. 10.1039/D2RA05297K. 36337973 PMC9597288

[ref32] Huang J.; Yang L.; Chen Z.; Zhou Y.; Zeng S. DFT/TDDFT in Silico Design of Ullazine-Derived D-π-A-π-A Dye Photosensitiser. New J. Chem. 2023, 47 (23), 11030–11039. 10.1039/D3NJ00519D.

[ref33] Wheeler D. L.; Rainwater L. E.; Green A. R.; Tomlinson A. L. Modeling Electrochromic Polydioxythiophene-Containing Materials Through TDDFT. Phys. Chem. Chem. Phys. 2017, 19 (30), 20251–20258. 10.1039/C7CP04130F. 28726889

[ref34] Christiansen D. T.; Ohtani S.; Chujo Y.; Tomlinson A. L.; Reynolds J. R. All Donor Electrochromic Polymers Tunable Across the Visible Spectrum Via Random Copolymerization. Chem. Mater. 2019, 31 (17), 6841–6849. 10.1021/acs.chemmater.9b01293.

[ref35] Chen G.; Shen Z.; Iyer A.; Ghumman U. F.; Tang S.; Bi J.; Chen W.; Li Y. Machine-Learning-Assisted de Novo Design of Organic Molecules and Polymers: Opportunities and Challenges. Polymers (Basel). 2020, 12 (1), 163 10.3390/polym12010163. 31936321 PMC7023065

[ref36] Schleder G. R.; Padilha A. C. M.; Acosta C. M.; Costa M.; Fazzio A. From DFT to Machine Learning: Recent Approaches to Materials Science - A Review. J. Phys. Mater. 2019, 2 (3), 032001 10.1088/2515-7639/ab084b.

[ref37] Collier G. S.; Wilkins R.; Tomlinson A. L.; Reynolds J. R. Exploring Isomeric Effects on Optical and Electrochemical Properties of Red/Orange Electrochromic Polymers. Macromolecules 2021, 54, 1677–1692. 10.1021/acs.macromol.0c02719.

[ref38] Kiely E.; Zwane R.; Fox R.; Reilly A. M.; Guerin S. Density Functional Theory Predictions of the Mechanical Properties of Crystalline Materials. CrystEngComm 2021, 23 (34), 5697–5710. 10.1039/D1CE00453K.

[ref39] Weigert F. J.; Roberts J. D. ^13^C Nuclear Magnetic Resonance Spectroscopy. Determination of Carbon-Fluorine Couplings. J. Am. Chem. Soc. 1971, 93 (10), 2361–2369. 10.1021/ja00739a001.

[ref40] Teran N. B.; Reynolds J. R. Discrete Donor-Acceptor Conjugated Systems in Neutral and Oxidized States: Implications toward Molecular Design for High Contrast Electrochromics. Chem. Mater. 2017, 29 (3), 1290–1301. 10.1021/acs.chemmater.6b04725.

[ref41] Nielsen C. B.; Angerhofer A.; Abboud K. A.; Reynolds J. R. Discrete Photopatternable π-Conjugated Oligomers for Electrochromic Devices. J. Am. Chem. Soc. 2008, 130 (30), 9734–9746. 10.1021/ja7112273. 18593166

[ref42] Christiansen D. T.; Wheeler D. L.; Tomlinson A. L.; Reynolds J. R. Electrochromism of Alkylene-Linked Discrete Chromophore Polymers with Broad Radical Cation Light Absorption. Polym. Chem. 2018, 9 (22), 3055–3066. 10.1039/C8PY00385H.

[ref43] Nishinaga T.; Sotome Y. Stable Radical Cations and Their π-Dimers Prepared from Ethylene- and Propylene-3,4-Dioxythiophene Co-Oligomers: Combined Experimental and Theoretical Investigations. J. Org. Chem. 2017, 82 (14), 7245–7253. 10.1021/acs.joc.7b00816. 28650158

[ref44] Murai M.; Ku S. Y.; Treat N. D.; Robb M. J.; Chabinyc M. L.; Hawker C. J. Modulating Structure and Properties in Organic Chromophores: Influence of Azulene as a Building Block. Chem. Sci. 2014, 5 (10), 3753–3760. 10.1039/C4SC01623H.

[ref45] Sprutta N.; Siczek M.; Latos-Grazyński L.; Pawlicki M.; Szterenberg L.; Lis T. Dioxadiazuliporphyrin: A near-IR Redox Switchable Chromophore. J. Org. Chem. 2007, 72 (25), 9501–9509. 10.1021/jo7015218. 18001092

[ref46] Canjeevaram Balasubramanyam R. K.; Kumar R.; Ippolito S. J.; Bhargava S. K.; Periasamy S. R.; Narayan R.; Basak P. Quadrupolar (A-π-D-π-A) Tetra-Aryl 1,4-Dihydropyrrolo[3,2-*b*]Pyrroles as Single Molecular Resistive Memory Devices: Substituent Triggered Amphoteric Redox Performance and Electrical Bistability. J. Phys. Chem. C 2016, 120 (21), 11313–11323. 10.1021/acs.jpcc.5b11509.

[ref47] Sadowski B.; Hassanein K.; Ventura B.; Gryko D. T. Tetraphenylethylenepyrrolo[3,2-*b*]Pyrrole Hybrids as Solid-State Emitters: The Role of Substitution Pattern. Org. Lett. 2018, 20 (11), 3183–3186. 10.1021/acs.orglett.8b01011. 29790766

[ref48] Lindbloom B. J. CIE Color Calculator. http://www.brucelindbloom.com/index.html?ColorCalculator.html (accessed 2023-01-31).

[ref49] Lo C. K.; Shen D. E.; Reynolds J. R. Fine-Tuning the Color Hue of π-Conjugated Black-to-Clear Electrochromic Random Copolymers. Macromolecules 2019, 52 (17), 6773–6779. 10.1021/acs.macromol.9b01443.

[ref50] Ryu H. G.; Mayther M. F.; Tamayo J.; Azarias C.; Espinoza E. M.; Banasiewicz M.; Łukasiewicz Ł. G.; Poronik Y. M.; Jezewski A.; Clark J.; et al. Bidirectional Solvatofluorochromism of a Pyrrolo[3,2-*b*]Pyrrole-Diketopyrrolopyrrole Hybrid. J. Phys. Chem. C 2018, 122 (25), 13424–13434. 10.1021/acs.jpcc.7b11194.

[ref51] Canjeevaram Balasubramanyam R. K.; Kandjani A. E.; Harrison C. J.; Abdul Haroon Rashid S. S. A.; Sabri Y. M.; Bhargava S. K.; Narayan R.; Basak P.; Ippolito S. J. 1,4-Dihydropyrrolo[3,2-*b*]Pyrroles as a Single Component Photoactive Layer: A New Paradigm for Broadband Detection. ACS Appl. Mater. Interfaces 2017, 9 (33), 27875–27882. 10.1021/acsami.7b08906. 28777542

[ref52] Bulumulla C.; Gunawardhana R.; Gamage P. L.; Miller J. T.; Kularatne R. N.; Biewer M. C.; Stefan M. C. Pyrrole-Containing Semiconducting Materials: Synthesis and Applications in Organic Photovoltaics and Organic Field-Effect Transistors. ACS Appl. Mater. Interfaces 2020, 12 (29), 32209–32232. 10.1021/acsami.0c07161. 32584535

[ref53] Domínguez R.; Montcada N. F.; de la Cruz P.; Palomares E.; Langa F. Pyrrolo[3,2-*b*]Pyrrole as the Central Core of the Electron Donor for Solution-Processed Organic Solar Cells. ChemPlusChem 2017, 82 (7), 1096–1104. 10.1002/cplu.201700158. 31961618

[ref54] Wang J.; Chai Z.; Liu S.; Fang M.; Chang K.; Han M.; Hong L.; Han H.; Li Q.; Li Z. Organic Dyes Based on Tetraaryl-1,4-Dihydropyrrolo-[3,2-*b*]Pyrroles for Photovoltaic and Photocatalysis Applications with the Suppressed Electron Recombination. Chem. - Eur. J. 2018, 24 (68), 18032–18042. 10.1002/chem.201803688. 30307090

[ref55] Krzeszewski M.; Thorsted B.; Brewer J.; Gryko D. T. Tetraaryl-, Pentaaryl-, and Hexaaryl-1,4-Dihydropyrrolo[3,2-*b*]Pyrroles: Synthesis and Optical Properties. J. Org. Chem. 2014, 79, 3119–3128. 10.1021/jo5002643. 24655027

[ref56] Krzeszewski M.; Kodama T.; Espinoza E. M.; Vullev V. I.; Kubo T.; Gryko D. T. Nonplanar Butterfly-Shaped π-Expanded Pyrrolopyrroles. Chem. - Eur. J. 2016, 22 (46), 16478–16488. 10.1002/chem.201603282. 27659591

[ref57] Liu H.; Ye J.; Zhou Y.; Fu L.; Lu Q.; Zhang C. New Pyrrolo[3,2-*b*]Pyrrole Derivatives with Multiple-Acceptor Substitution: Efficient Fluorescent Emission and near-Infrared Two-Photon Absorption. Tetrahedron Lett. 2017, 58 (52), 4841–4844. 10.1016/j.tetlet.2017.11.028.

[ref58] Banasiewicz M.; Stężycki R.; Kumar G. D.; Krzeszewski M.; Tasior M.; Koszarna B.; Janiga A.; Vakuliuk O.; Sadowski B.; Gryko D. T.; Jacquemin D. Electronic Communication in Pyrrolo[3,2-*b*]Pyrroles Possessing Sterically Hindered Aromatic Substituents. Eur. J. Org. Chem. 2019, 2019, 5247–5253. 10.1002/ejoc.201801809.

[ref59] Cardona C. M.; Li W.; Kaifer A. E.; Stockdale D.; Bazan G. C. Electrochemical Considerations for Determining Absolute Frontier Orbital Energy Levels of Conjugated Polymers for Solar Cell Applications. Adv. Mater. 2011, 23 (20), 2367–2371. 10.1002/adma.201004554. 21462372

[ref60] Frisch M. J.; Trucks G. W.; Schlegel H. B.; Scuseria G. E.; Robb M. A.; Cheeseman J. R.; Scalmani G.; Barone V.; Petersson G. A.; Nakatsuji H.; Gaussian 16; Gaussian, Inc.: Wallingford, CT, 2016.

