# Supplementary material for: Expanding Color Control of Anodically Coloring Electrochromes Based on Electron-Rich 1,4-Dihydropyrrolo[3,2-b]pyrroles
Source: ACS Appl Opt Mater. 2024 Jun 12;2(6):1235–44. doi: 10.1021/acsaom.4c00197 (PMC11217944; doi:10.1021/acsaom.4c00197)
Supplement: Supplementary file 1 — ot4c00197_si_001.pdf [file ot4c00197_si_001.pdf]

Supporting Information

Expanding Color-Control of Anodically Coloring Electrochromes Based on Electron-Rich 1,4-Dihydropyrrolo[3,2-*b*]pyrroles

Allison M. Hawks,[1] Lillian M. Daniel,[1] Valentino S. Sorto,[1] Julia Mauro, [1] Perry Skiouris,[1] and Graham S. Collier[1,2]*

[1]Department of Chemistry and Biochemistry, Kennesaw State University, Kennesaw, GA, 30144, United States

[2]School of Polymer Science and Engineering, University of Southern Mississippi, Hattiesburg, MS, 39406, United States

*E-mail graham.collier@usm.edu

Materials and Methods

TD-DFT calculations using Gaussian 16[1] and the B3LYP-631G* functional/basis set were performed to elucidate the optical properties of the DHPP molecules that are synthetic targets. First, the molecules were constructed in Gaussview and a geometry optimization was performed to ensure the correct geometry was used in the subsequent calculations. Next, the excited state calculations were run to understand the positioning of the radical cation absorbance. After completion, the data was collected, normalized to the absorbance maximum, and plotted in Origin to report calculated UV-vis absorbance spectra.

All materials were purchased from commercial sources and used as received unless otherwise stated. Anhydrous dichloromethane (DCM) and toluene were obtained from a Pure Process Technology GC-SPS-7 Glass Contour 800L Solvent Purification System stored under Ar and degassed with argon (Ar) for 15 min. before use. Anhydrous dimethylformamide (DMF) was purchased from Fisher Scientific and was degassed with Ar for 15 min. before use. ^{1}H NMR and ^{13}C NMR spectra were collected on a Bruker Advance III HD 400 MHz NMR spectrometer with nominal concentrations of 5 mg/mL in $CDCl_3$. Peaks are referenced to the residual $CHCl_3$ peak (^{1}H: $\delta = 7.26$ ppm; ^{13}C: $\delta = 77.23$ ppm). Optical absorbance spectra were acquired using a Varian Cary 60 Scan single-beam UV−vis−near-IR spectrophotometer scanning from 300 to 800 nm. Sample solutions were made to concentrations of 12 – 24 mM in DCM. Each molecule and polymer were titrated dropwise with a 0.6 mg/mL $Fe(ClO_4)_3$xH$_2$O solution in ethyl acetate until the radical cation peak reached its maximum absorbance intensity. Next to the UV-vis absorbance spectra are photographs taken of neutral and oxidized solutions in quartz cuvettes after the addition of the maximum amount of oxidant. Photographs are presented without manipulation except for cropping. Colorimetric analysis was performed using a programmed excel sheet based on the Commission Internationale de l'Eclairage 1976 *L*a*b** color standards using a D50 illuminant as a 2˚ observer.[2] Cyclic voltammetry (CV) and differential pulse voltammetry (DPV) measurements were performed with a CH Instruments electrochemical workstation (CHI660D), using a glassy carbon electrode as the working electrode, an Ag/AgCl reference electrode (calibrated versus the Fc/Fc$^+$ redox couple, $E_{1/2} = 40$ mV), and a Pt wire as the counter electrode. A 50 mV/s scan rate was used for all electrochemical measurements. An electrolyte solution of 0.5 M tetrabutylammonium hexafluorophosphate ($TBAPF_6$, 98%) in anhydrous DCM was used for all molecular electrochemical measurements. The electrochemical cell used to capture color changes

upon electrochemical oxidation was a SEC-C thin-layer quartz glass spectroelectrochemical cell with a platinum gauze working electrode. Photography was performed in a light booth designed to exclude outside light with controllable LED lighting above providing illumination. A Canon Rebel T7 camera with an 18-55 mm lens was used to capture images. Images are presented without manipulations except for cropping. There are no hidden risks or hazards to declare for this work.

Synthesis of DHPP Functionalized Molecules via an Fe(III)-Catalyzed Multicomponent Reaction

The desired alkyl-aniline (8 mmol) and functionalized benzaldehyde (8 mmol) were added to a solution of toluene (6 mL) and glacial acetic acid (6 mL) inside a 25 mL round bottom flask that was equipped with a magnetic stir bar. This reaction mixture was stirred at 50 °C for 1 h. Once the initial heating time was completed, $Fe(ClO_4)_3 \cdot xH_2O$ (0.085 g) was added to the reaction flask, followed by 2,3-butanedione (0.35 mL, 4.00 mmol). After these additions, the reaction was allowed to stir at 50 ˚C overnight. The next day the reaction was removed from heat and allowed to cool to room temperature. Next, the reaction flask was placed in an ice bath to encourage the formation of a precipitate which was then collected via vacuum filtration followed by washes with cold MeOH and acetone to reveal a white or pale-yellow solid on the filter paper. Finally, the precipitate was transferred to a vial and dried overnight under vacuum. After structural analysis, each molecule was confirmed to be the desired product.

2,5-bis(4-fluorophenyl)-1,4-bis(4-*n*-decylphenyl)-1,4-dihydropyrrolo[3,2-*b*]pyrrole (4-FDHPP): White solid. Yield: 1.13 g (39%). ^{1}H NMR: (400 MHz, $CDCl_3$), δ: 0.91 (t, 6H), 1.30-1.36 (m, 30H), 1.62-1.70 (m, 4H), 2.64 (t, 4H), 6.36 (s, 2H), 6.92-6.96 (m, 4H), 7.18-7.22 (m, 13H). ^{13}C NMR: (400 MHz, $CDCl_3$), δ: 14.1, 22.7, 29.4, 29.5, 29.6, 31.4, 31.9, 35.5, 94.3, 115.1 (d, J = 21.5 Hz), 125.1, 129.0, 129.8 (d, J = 7.8 Hz), 130.0 (d, J = 3.3 Hz), 131.3, 134.7, 137.4,

140.6, 161.5 (d, J = 245.9 Hz). Anal. calc'd for $C_{50}H_{60}F_2N_2$ Theory: C, 82.60; H, 8.32; N, 3.85 Actual: C, 82.74; H, 8.37; N, 3.97.

2,5-bis(3,4-difluorophenyl)-1,4-bis(4-*n*-decylphenyl)-1,4-dihydropyrrolo[3,2-*b*]pyrrole (3,4-F_2DHPP): Pale-yellow solid. Yield: 1.05 g (34%). ^{1}H NMR: (400 MHz, $CDCl_3$), δ: 0.91 (t, 6H), 1.30-1.39 (m, 30H), 1.63-1.70 (m, 4H), 2.64 (t, 4H), 6.36 (s, 2H), 6.91-6.95 (m, 2H), 6.98-7.05 (m, 4H), 7.16-7.22 (m, 9H). ^{13}C NMR: (400 MHz, $CDCl_3$), δ: 14.1, 22.7, 29.3, 29.5, 29.6, 31.3, 31.9, 35.5, 94.7, 116.7, 116.9 (d, J = 5.3), 117.1, 124.0, 125.1, 129.2, 130.8 (m, J = 7.0 Hz), 131.7, 134.0, 137.0, 141.1, 147.7 (d, J = 12.8 Hz), 148.8 (d, J = 12.8 Hz), 149.0 (dd, J = 248.0 Hz), 150.3 (d, J = 12.8 Hz), 151.2 (d, J = 12.8 Hz), 150.0 (dd, J = 247.0 Hz). Anal. calc'd for $C_{50}H_{58}F_4N_2$ Theory: C, 78.71; H, 7.66; N, 3.67 Actual: C, 78.71; H, 7.61; N, 3.61.

2,5-bis(3,4,5-trifluorophenyl)-1,4-bis(4-*n*-decylphenyl)-1,4-dihydropyrrolo[3,2-*b*]pyrrole (3,4,5-F_3DHPP): Pale-yellow solid. Yield: 1.01 g (34%). ^{1}H NMR: (400 MHz, $CDCl_3$), δ: 0.91 (t, 6H), 1.30-1.37 (m, 31H), 1.64-1.71 (m, 4H), 2.68 (t, 4H), 6.36 (s, 2H), 6.77-6.83 (m, 4H), 7.17 (d, J = 8.3 Hz, 4H), 7.24 (d, J = 8.3 Hz, 4H). ^{13}C NMR: (400 MHz, $CDCl_3$), δ: 14.1, 22.7, 29.3, 29.4, 29.5, 29.6, 31.3, 31.9, 35.5, 95.1, 111.7 (dd, J = 22.0 Hz), 125.2, 129.5, 132.2, 133.4, 136.6, 141.6, 150.9 (dt J = 235.0 Hz). Anal. Calc'd for $C_{50}H_{56}F_2N_2$ Theory: C, 75.16; H, 7.06; N, 3.51 Actual: C, 74.89; H, 6.92; N, 3.57.

2,5-bis(4-fluoro-3-methoxyphenyl)-1,4-bis(4-*n*-decylphenyl)-1,4-dihydropyrrolo[3,2-*b*]pyrrole (F,OMeDHPP): Beige solid. Yield: 500 mg (16%). ^{1}H NMR: (400 MHz, $CDCl_3$), δ: 0.91 (t, 7H), 1.30-1.39 (m, 32H), 1.61-1.69 (m, 5H), 2.65 (t, 4H), 3.63 (s, 6H), 6.38 (s, 2H), 6.73-6.75 (dd, 2H), 6.81-6.84 (m, 2H), 6.94-6.99 (m, 2H), 7.21 (s, 9H). ^{13}C NMR: (400 MHz, $CDCl_3$), δ: 14.1, 22.7, 29.3, 29.5, 29.6, 31.5, 31.9, 35.5, 55.8, 94.1, 113.5, 115.8 (d, J = 18.5 Hz), 120.4 (d, J = 6.5 Hz), 125.3, 129.1, 130.2 (d, J = 3.8 Hz), 131.4, 135.0, 137.5, 140.8, 147.0 (d, J = 10.9 Hz),

151.1 (d, $J = 245.7$ Hz). Anal. Calc'd for $C_{52}H_{64}F_2N_2O_2$ Theory: C, 79.35; H, 8.20; N, 3.56 Actual: C, 79.40; H, 8.33; N, 3.69.

2,5-bis(4-methoxyphenyl)-1,4-bis(4-*n*-decylphenyl)-1,4-dihydropyrrolo[3,2-*b*]pyrrole (4-OMeDHPP): Yellow solid. Yield: 313 mg (5%). ^{1}H NMR: (400 MHz, $CDCl_3$), δ: 0.91 (t, 7H), 1.30-1.39 (m, 35H), 1.62-1.69 (m, 5H), 2.64 (t, 4H), 3.77-3.82 (m, 7H), 6.33 (s, 2H), 6.78-6.81 (m, 4H), 7.16-7.22(m, 13H). ^{13}C NMR: (400 MHz, $CDCl_3$), δ: 14.1, 22.7, 29.4, 29.5, 29.7, 31.4, 31.9, 35.5, 55.2, 93.8, 113.6, 125.0, 126.7, 128.9, 129.5, 131.0, 135.2, 137.8, 140.2. Analytical data aligns with previously published molecules.

2,5-bis(4-meththiophenyl)-1,4-bis(4-*n*-decylphenyl)-1,4-dihydropyrrolo[3,2-*b*]pyrrole (4-SMeDHPP): Yellow solid. Yield: 519.1 mg (8%). ^{1}H NMR (400 MHz, $CDCl_3$), δ: 0.91 (t, 6H), 1.35 (t, 29 H), 1.67 (m, 4H), 2.48 (s, 6H) 2.65 (t, 4H), 6.38 (s, 2H), 7.17 (m, 16H). ^{13}C NMR (400 MHz, $CDCl_3$), δ: 14.1, 15.8, 22.7, 29.4, 29.5, 29.7, 31.4, 31.9, 35.5, 77.2, 94.3, 125.1, 126.3, 128.4, 129.0, 130.7, 131.7, 135.3, 135.8, 137.6, 140.5. Anal. calc'd for $C_{52}H_{66}N_2S_2$ Theory: C, 79.74; H, 8.49; N, 3.58; S, 8.19. Actual: C, 79.26; H, 8.38; N, 3.62; S, 8.05.

2,5-bis(diyl)bis(*N,N*-di-*p*-tolylamine)-1,4-bis(4-*n*-decylphenyl)-1,4-dihydropyrrolo[3,2-*b*]pyrrole (4-tol$_2$ADHPP): Yellow solid. Yield: 345.2 mg (4%). ^{1}H NMR (400 MHz, $CDCl_3$), δ: 0.90 (t, 8H), 1.30 (m, 37H), 1.64 (m, 5H), 2.32 (s, 15H), 2.64 (t, 5H), 6.34 (s, 2H), 6.87 (d, 6H), 7.05 (dd, 24H), 7.16 (d, 11H). ^{13}C NMR (400 MHz, $CDCl_3$), δ: 14.1, 20.8, 22.7, 29.4, 29.4, 29.5, 29.6, 29.7, 31.4, 31.9, 35.5, 77.2, 93.7, 122.0, 124.6, 125.1, 127.2, 128.7, 128.8, 129.8, 131.3, 132.4, 135.5, 137.9, 140.2, 145.3, 146.2. Anal. calc'd for $C_{78}H_{88}N_4$. Theory: C, 88.62; H, 8.20; N, 5.18. Actual: C, 86.68; H, 8.19; N, 5.26.

NMR Spectroscopy

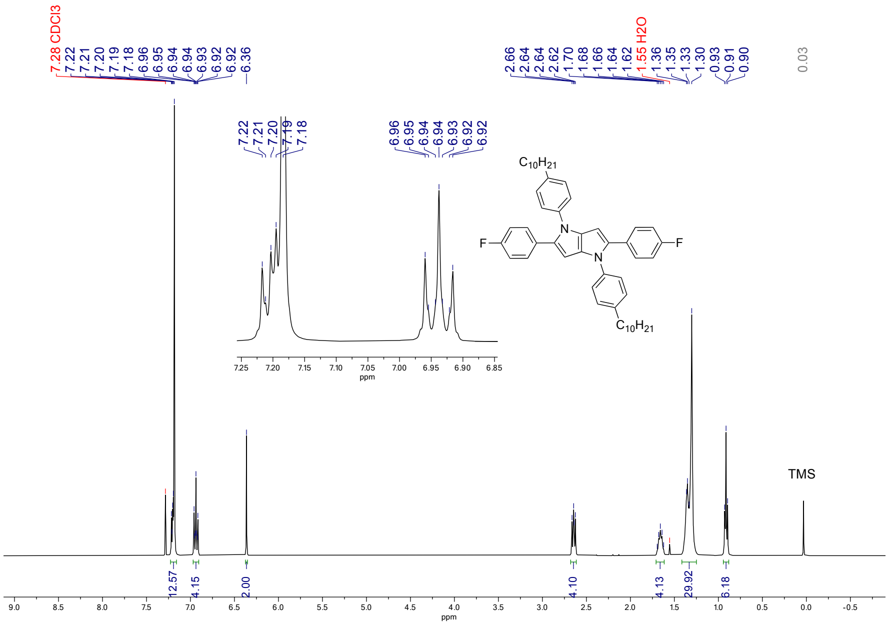

Figure S1. ^{1}H NMR (400 MHz, 25 ˚C, $CDCl_3$) of 4-FDHPP.

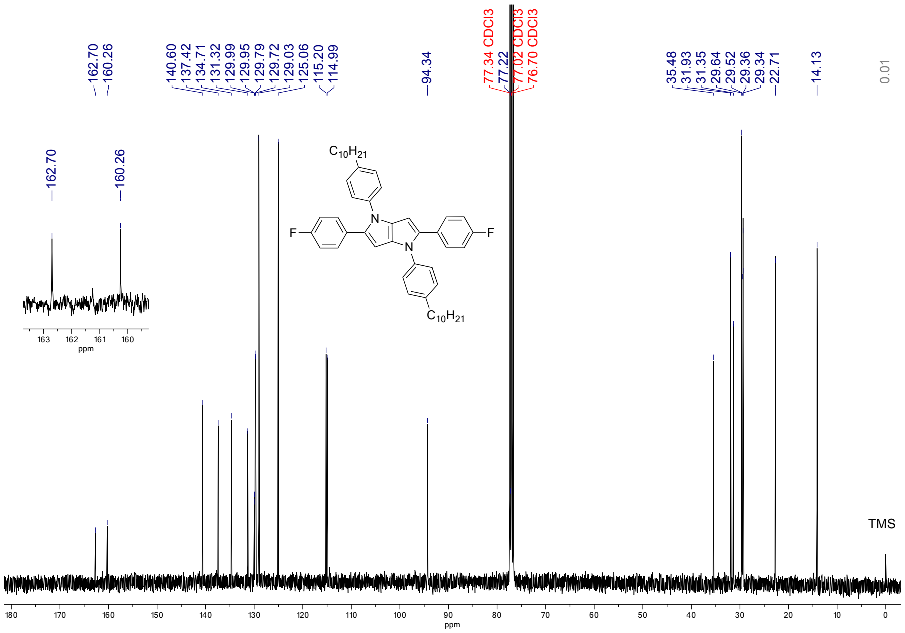

Figure S2. ^{13}C NMR (400 MHz, 25 ˚C, $CDCl_3$) of 4-FDHPP.

Table S1. *J*-coupling constants and peak assignments for the ^{13}C NMR of 4-FDHPP. Calculated coupling constants are consistent with aromatic carbon-fluorine coupling constants reported by Weigert and coworkers.[3]

**Chemical Shift (ppm)**	**Peak**	**Coupling Constant *J* (Hz)**	**Interaction**
161.5	d	245.9	^{1}J (C-F)
115.1	d	21.5	^{2}J (C-C-F)
129.8	d	7.8	^{3}J (C-C-C-F)
130.0	d	3.3	^{4}J (C-C-C-C-F)

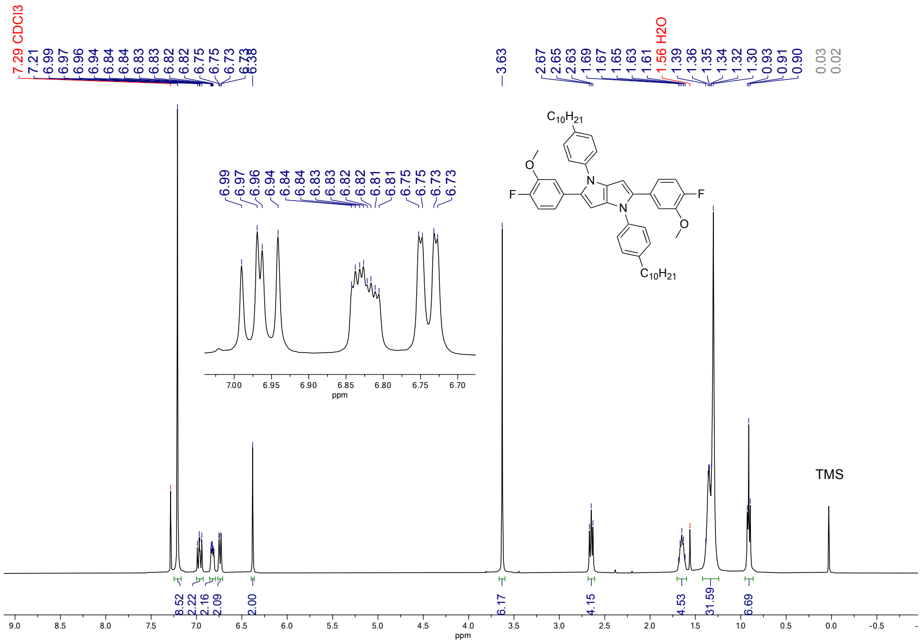

Figure S3. ^{1}H NMR (400 MHz, 25 ˚C, $CDCl_3$) of F,OMeDHPP.

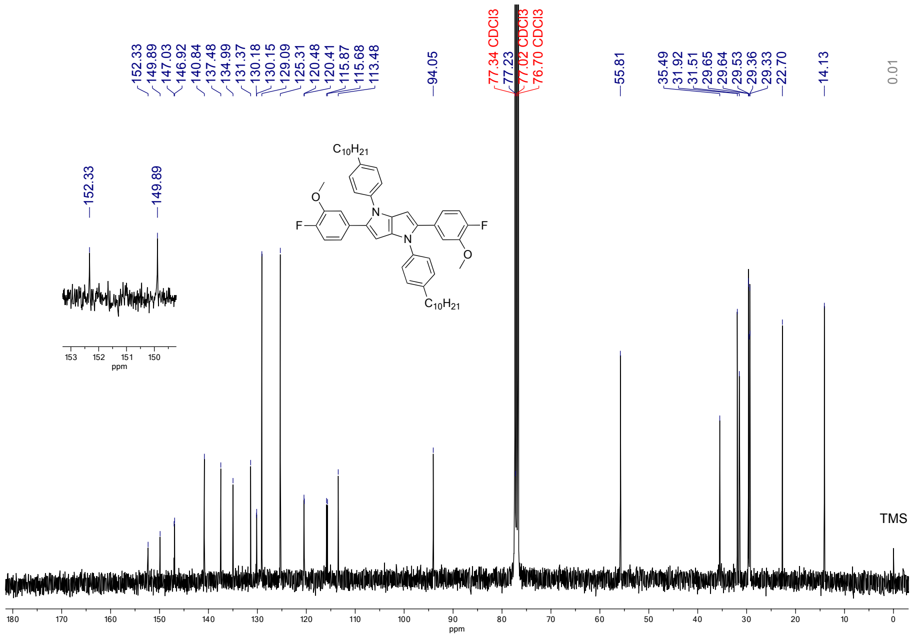

Figure S4. ^{13}C NMR (400 MHz, 25 ˚C, $CDCl_3$) of F,OMeDHPP.

Table S2. *J*-coupling constants and peak assignments for the ^{13}C NMR of F,OMeDHPP. Calculated coupling constants are consistent with aromatic carbon-fluorine coupling constants reported by Weigert and coworkers.[3]

Chemical Shift (ppm)	Peak	Coupling Constant *J* (Hz)	Interaction
151.1	d	246	^1J (C-F)
147.0	d	10.9	^2J (C-C-F)
115.8	d	18.5	^2J (C-C-F)
120.4	d	6.5	^3J (C-C-C-F)
130.2	d	3.8	^4J (C-C-C-C-F)

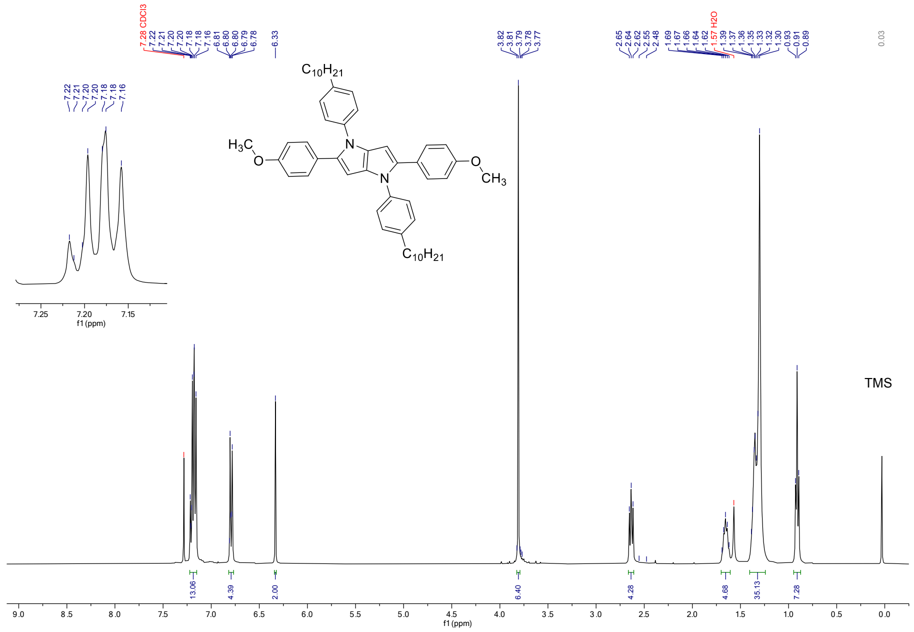

Figure S5. ^{1}H NMR (400 MHz, 25 ˚C, $CDCl_3$) of 4-OMeDHPP.

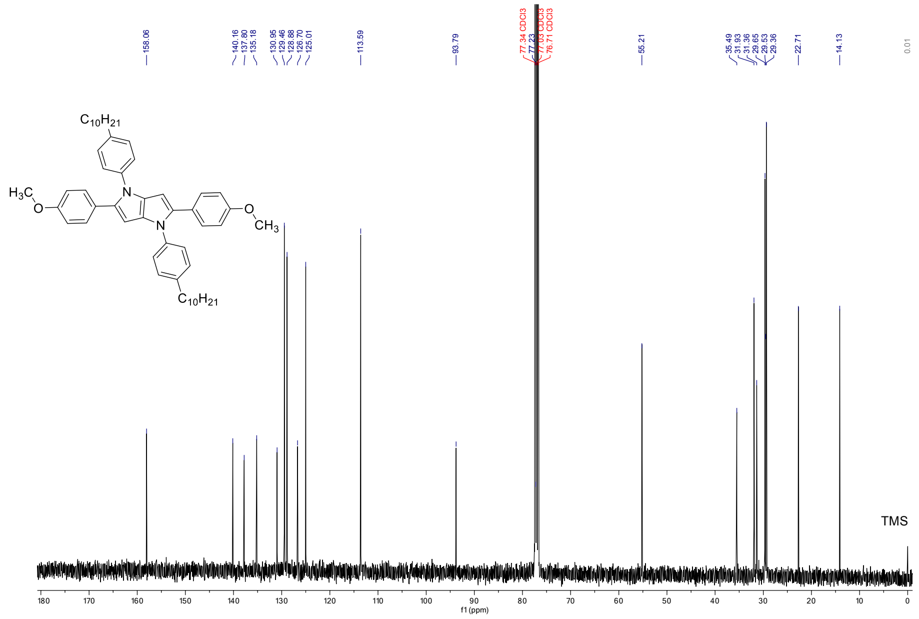

Figure S6. ^{13}C NMR (400 MHz, 25 ˚C, $CDCl_3$) of 4-OMeDHPP.

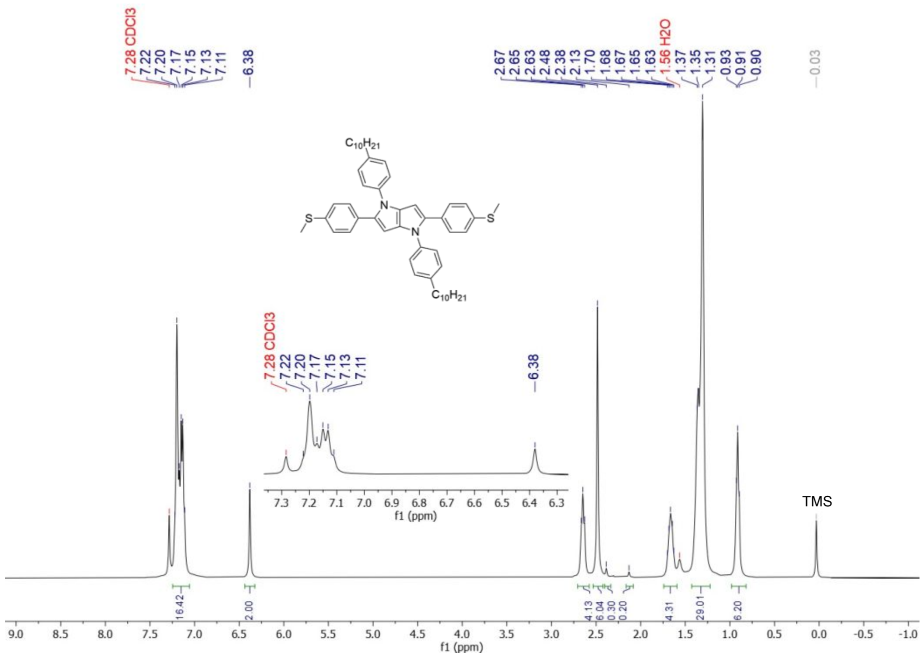

Figure S7. ^{1}H NMR (400 MHz, 25 ˚C, $CDCl_3$) of 4-SMeDHPP.

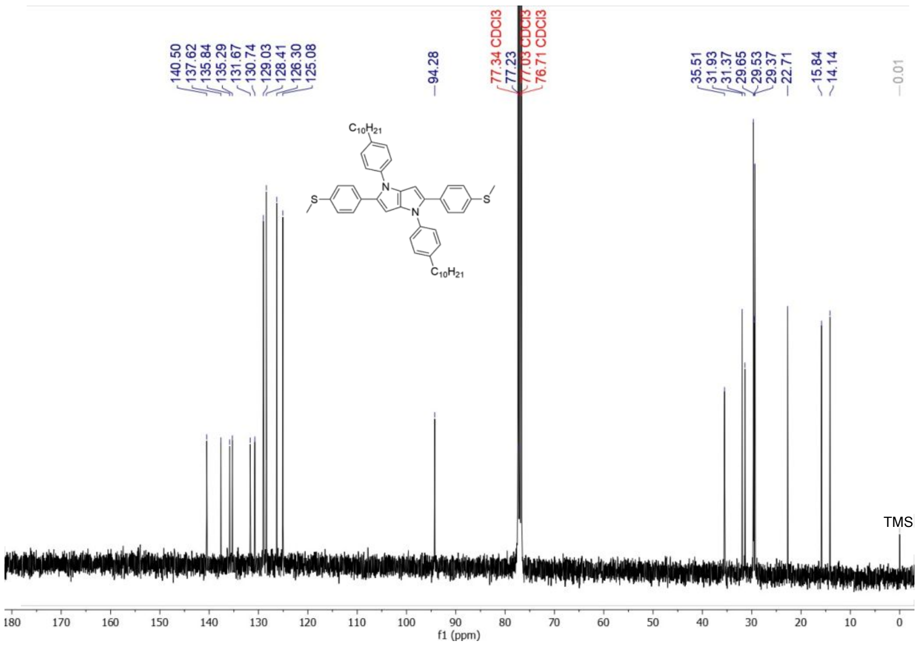

Figure S8. ^{13}C NMR (400 MHz, 25 ˚C, $CDCl_3$) of 4-SMeDHPP.

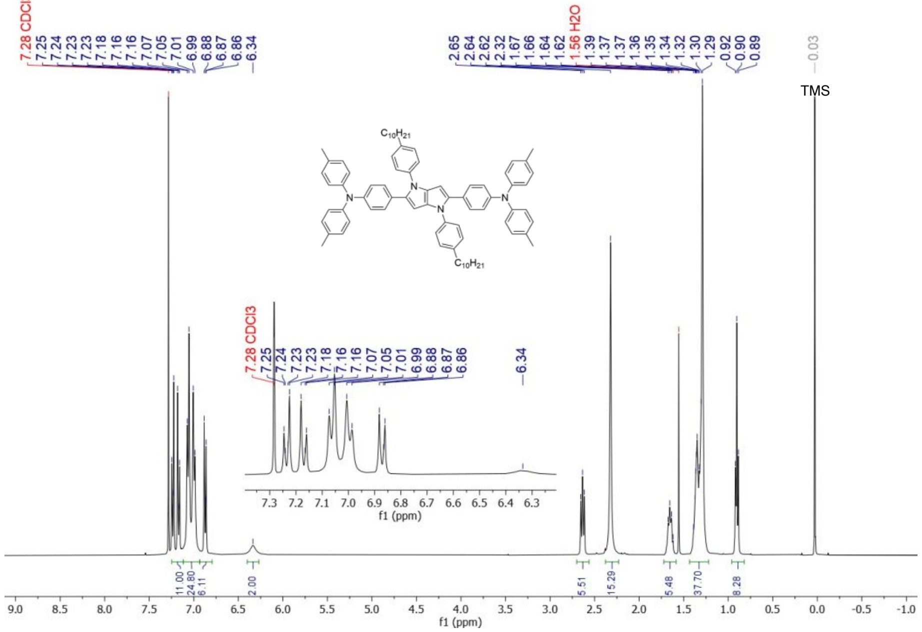

Figure S9. ^{1}H NMR (400 MHz, 25 ˚C, $CDCl_3$) of 4-tol$_2$ADHPP.

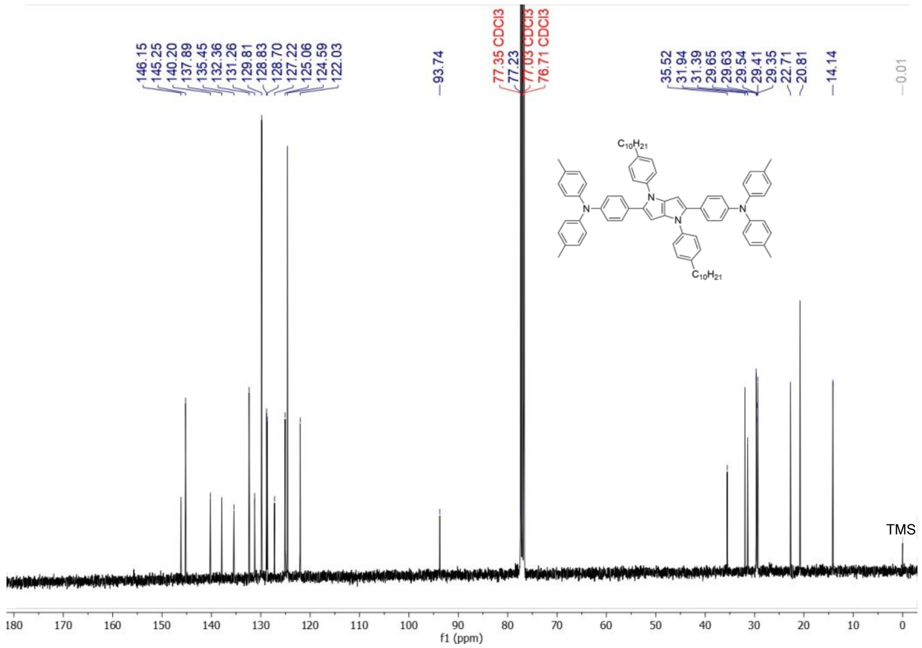

Figure S10. ^{13}C NMR (400 MHz, 25 ˚C, $CDCl_3$) of 4-tol$_2$ADHPP.

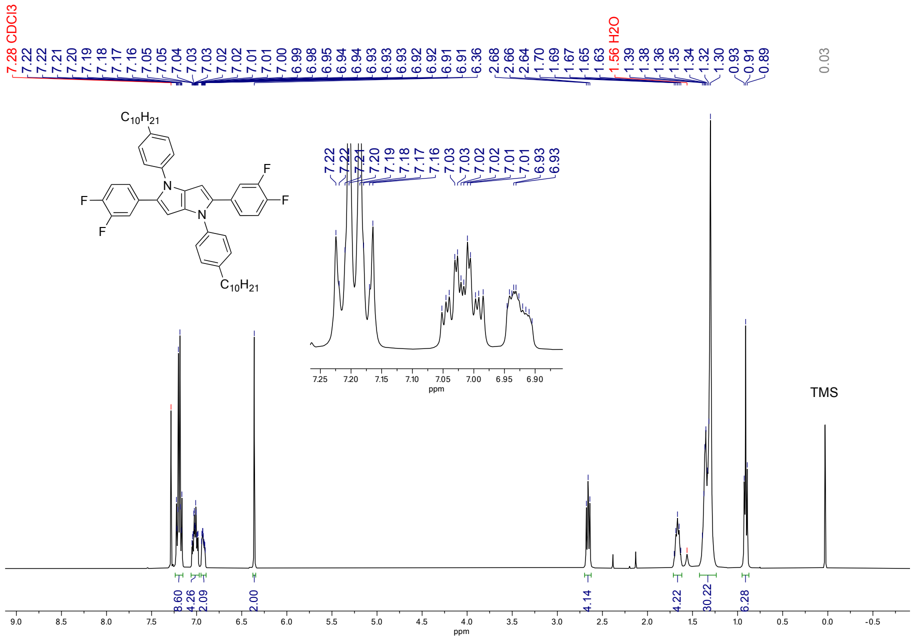

Figure S11. ^{1}H NMR (400 MHz, 25 ˚C, $CDCl_3$) of 3,4-F_2DHPP.

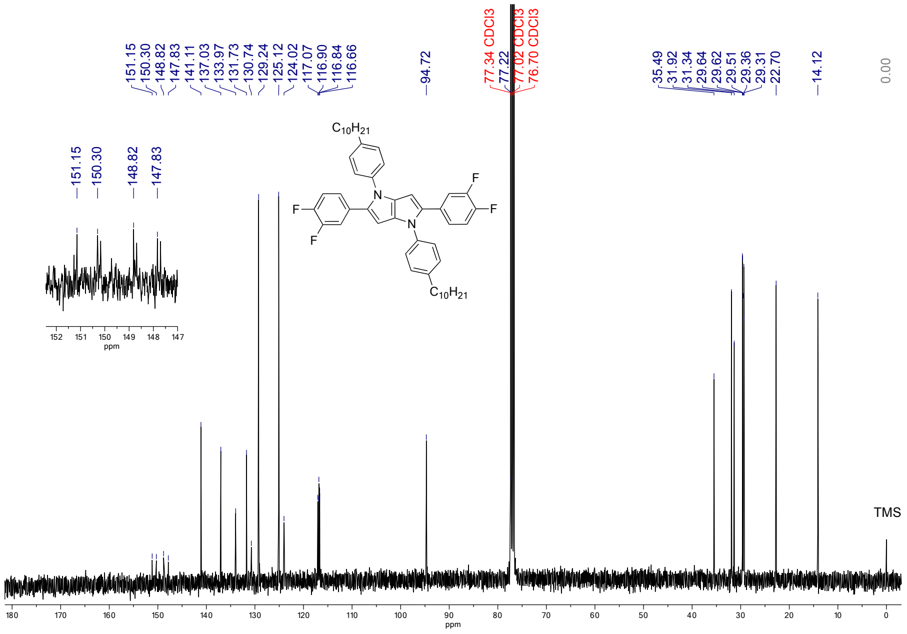

Figure S12. ^{13}C NMR (400 MHz, 25 ˚C, $CDCl_3$) of 3,4-F_2DHPP.

Table S3. *J*-coupling constants and peak assignments for the ^{13}C NMR of 3,4-F_2DHPP. Calculated coupling constants are consistent with aromatic carbon-fluorine coupling constants reported by Weigert and coworkers.[3]

Chemical Shift (ppm)	Peak	Coupling Constant *J* (Hz)	Interaction
150.0	dd	247.0	^1J (C-F^1)
149.1	dd	248.0	^1J (C-F^2)
151.2, 150.3, 148.8, 147.8	d	12.8	^2J (C-C-F BOTH)
130.8	m	7.0	^3J (C-C-C-F^1)
116.9	d	5.3	^2J (C-C-F^2)

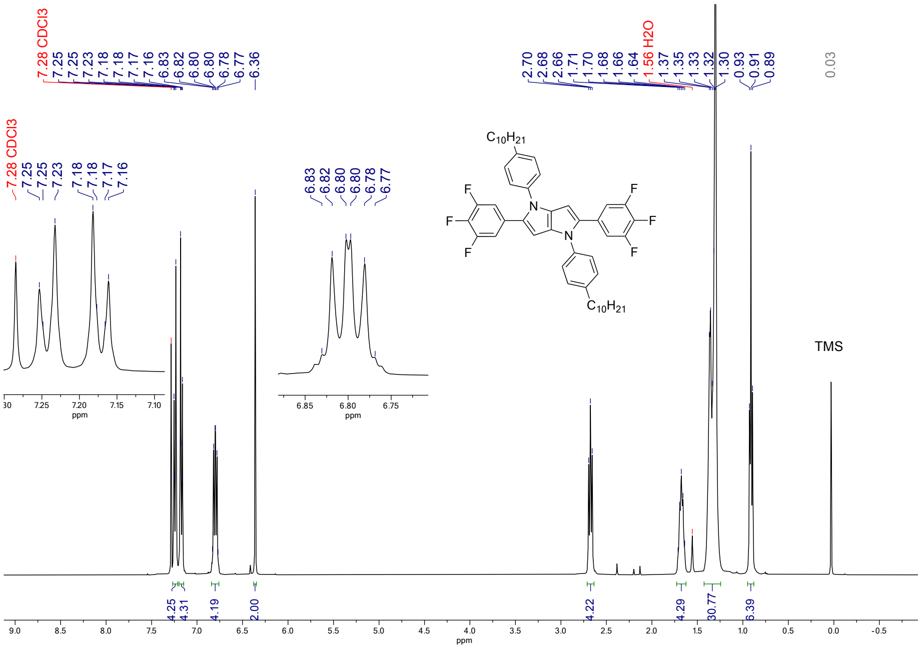

Figure S13. ^{1}H NMR (400 MHz, 25 ˚C, $CDCl_3$) of 3,4,5-F_3DHPP.

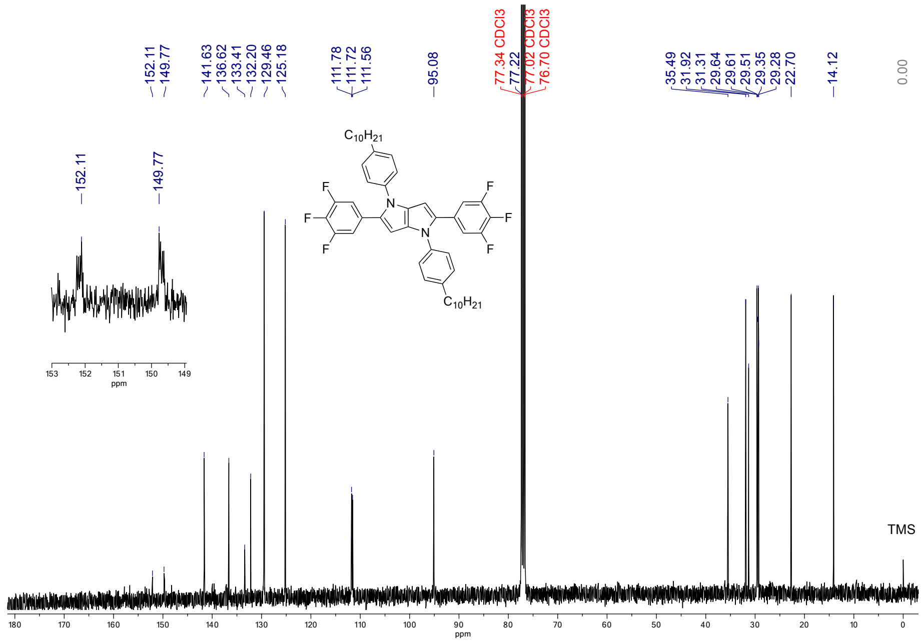

Figure S14. ^{13}C NMR (400 MHz, 25 ˚C, $CDCl_3$) of 3,4,5-F_3DHPP.

Table S4. *J*-coupling constants and peak assignments for the ^{13}C NMR of 3,4,5-F_3DHPP. Calculated coupling constants are consistent with aromatic carbon-fluorine coupling constants reported by Weigert and coworkers.[3]

Chemical Shift (ppm)	Peak	Coupling Constant *J* (Hz)	Interaction
150.9	dm	235.0	^1J (C-F^1)
111.7	dm	22.0	^2J (C-C-F^1)
111.8	d	6.0	^3J (C-C-C-F^1)
111.6	d	6.0	^3J (C-C-C-F^2)

Fluorinated DHPPs to Establish Agreement Between Theory and Experiment

Our theory-guided efforts began by benchmarking agreement between theory and experiment using model DHPP chromophores. Figure S15 shows the calculated UV-vis absorbance spectra of three DHPPs with varying degrees of fluorination as neutral and oxidized chromophores. The neutral absorbance spectra of this initial set of molecules are all predicted to be positioned in the UV region of the EMS, evident by their similar calculated absorbance maxima

(λ_{max}) shown in Figure S15(A). This is encouraging as absorbance in this region is the first criterion for anodically coloring electrochromes. However, the electron-withdrawing effect of fluorines at the *meta*-position does not seem to significantly impact the positioning of the radical cation based on the similar absorbance profiles represented by the dashed lines in Figure S15(A). The high-energy singly occupied molecular orbital transition (SOMO-α) to the lowest unoccupied molecular orbital (LUMO) (SOMO-α → LUMO-α) is ~ 450 nm for all of the molecules and the lower-energy SOMO→LUMO transition (SOMO-β → LUMO-β) has a broad peak of ~ 650 nm. While the predicted spectra do not show a large difference between the set of molecules, the three molecules were synthesized and their structure-property relationships were explored to determine the reliability of the TD-DFT calculations.

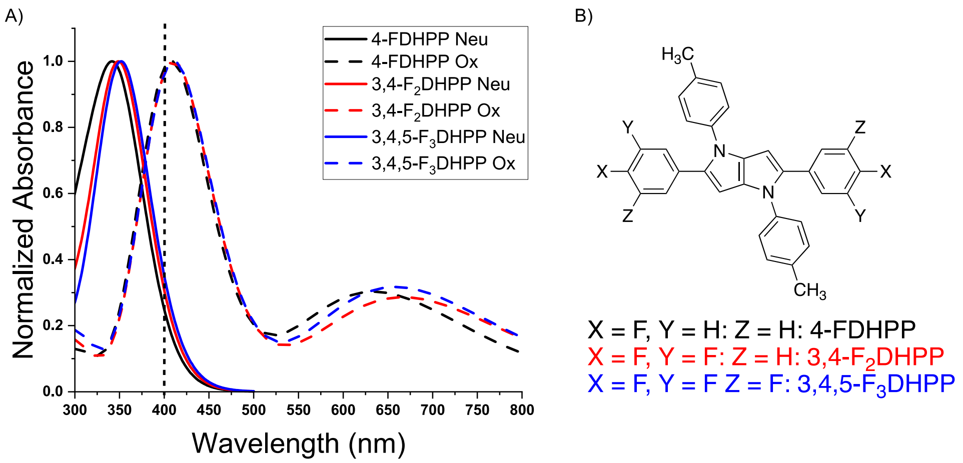

Figure S15. (A) Normalized UV-vis absorbance spectra generated from TD-DFT calculations of the fluorinated DHPP molecules using B3LYP-631G*. The neutral spectra are the solid lines while the oxidized are dashed. (B) Representative structures of the fluorinated DHPP molecules where the R groups were truncated to -CH_3 groups for simplicity.

In an effort to verify the level of theory used to predict the UV-vis absorbance of DHPP molecules, the calculated absorbance spectra were compared with the experimental (Figure S16). Importantly, the experimental and calculated neutral spectra for all three fluorinated DHPPs are aligned which is a strong indicator of the accuracy of the TD-DFT level of theory. There is a slight

increase in the neutral absorbance maxima wavelength (λ_{max}^{neu}) from 4-FDHPP (345 nm) to 3,4-F_2DHPP (350 nm) to 3,4,5-F_3DHPP (355 nm) and this red shift is attributed to the increasing electron-withdrawing nature with increasing fluorination. The results demonstrate that positioning the electron-withdrawing substituents at the *meta* and *ortho* positions does not significantly alter the neutral absorbance and keeps the neutral absorbance in the UV region on the EMS. Moving to the oxidized spectra, the experimental high energy transition, SOMO-α → LUMO-α, is red shifted compared to the calculated absorbance for each of the molecules resulting in absorbance maxima (λ_{max}^{α}) that are ~ 40 nm shifted from calculated to experimental. It is not uncommon for there to be discrepancies in λ_{max} of calculated and experimental absorbance because the predicted UV-vis spectra are Gaussian distributions of the allowed transitions.[4,5] Encouragingly there is significant overlap of calculated and experimental spectra across the visible spectrum and when combined with the accuracy for the calculated neutral spectra, this level of theory has been deemed suitable. The low energy SOMO-β → LUMO-β transition absorbance maxima (λ_{max}^{β}) are similar with the largest difference of these transitions between the molecules coming from the shape of the peaks. The SOMO-β of the experimental UV-vis lies within the broad peak for the calculated and, therefore, is considered an accurate representation of allowed transitions for the oxidized molecules. Overall, Figure S16 demonstrates that the level of theory is accurate in predicting the UV-vis absorbance for DHPP molecules in both their neutral and oxidized states which will be useful for continued design and discovery of novel electrochromes.

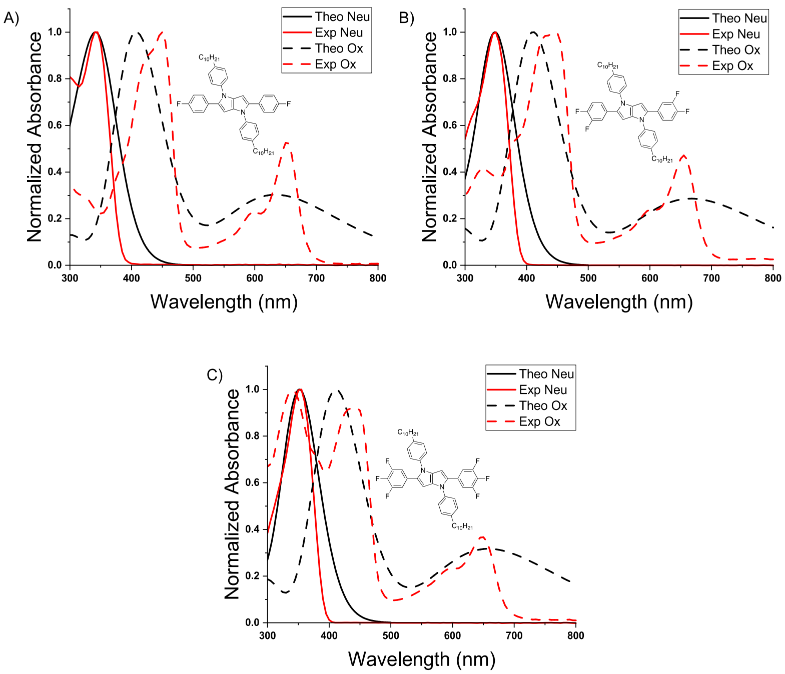

Figure S16. Overlayed calculated and experimental UV-vis absorbance spectra of (A) 4-FDHPP, (B) 3,4-F_2DHPP and (C) 3,4,5-F_3DHPP determining that the level of theory used for TD-DFT is reliable and accurate for the neutral and oxidized UV-vis absorbance spectra of DHPP chromophores.

Figure S17(A) displays the CV data for all three fluorinated DHPPs and shows the effect of increasing peripheral electronegativity of a DHPP on electrochemical properties. As illustrated in Figure S17(A) and tabulated in Table S5, an increase in the onset of oxidation (E_{onset}^{ox}) is seen from 0.42 V, 0.51 V, and 0.70 V (vs. Ag/AgCl) for 4-FDHPP to 3,4-F_2DHPP to 3,4,5-F_3DHPP, respectively (Table S5). The increase in E_{onset}^{ox} from 4-FDHPP to 3,4,5-F_3DHPP ($\Delta E \approx 0.29$ eV) is attributed to the increase in the electronegativity from the increased fluorine composition on the benzene rings that ultimately increases the difficulty of removing an electron from the molecule (Figure S17(A)).[6] The onsets of oxidation measured via DPV shown in Figure 17(B) agree with CV results showing an increase in E_{onset}^{ox} with increasing electronegativity. DPV also shows the second oxidation peak that is attributed to the formation of the dication of the DHPP molecules. The energy gaps for these DHPPs were calculated to be 3.1 eV and agrees with the molecules

absorbing in the UV region of the electromagnetic spectrum (Table S5). Given the energy gaps remain unchanged with varying functionality, these experiments show peripheral functionalities can manipulate the redox properties of DHPP electrochromes without sacrificing the optical properties required for ACE molecules. What is encouraging from these results is, that while the optical properties were not significantly influenced by the subtle changes in fluorination, the changes in redox response emphasizes an influence of peripheral functionalization that motivates alternative design strategies that will accomplish manipulation of both optical and redox properties.

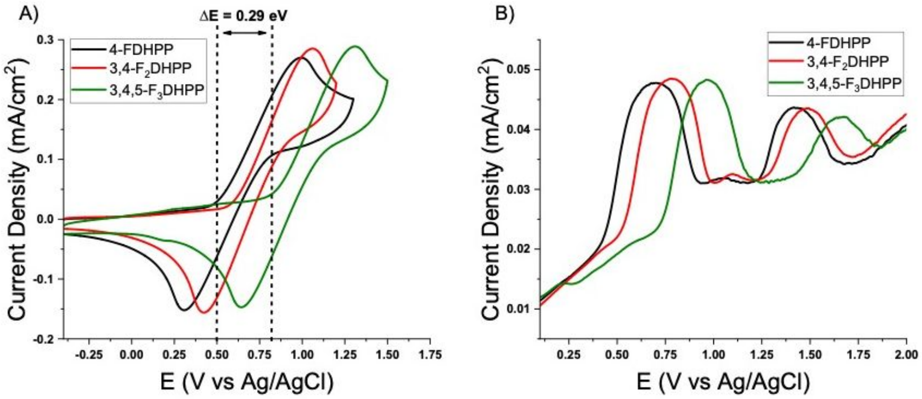

Figure S17. (A) CV traces from (-0.4 V to ~ 1.5 V) and (B) DPV traces from (-0.4 V to 2 V) of the fluorinated DHPP electrochromes against an Ag/AgCl reference electrode using a 0.5 M $TBAPF_6$/DCM supporting electrolyte.

Table S5. Optoelectronic properties of fluorinated DHPP molecules.

**Chromophore**	E_{onset}^{ox} **(V)**	**HOMO (eV)**[a]	**LUMO (eV)**[b]	E_{gap} **(eV)**[c]
4-FDHPP	0.42	-5.5	-2.4	3.1
3,4-F_2DHPP	0.51	-5.6	-2.5	3.1
3,4,5-F_3DHPP	0.70	-5.8	-2.7	3.1

[a]Calculated given HOMO = - (E_{onset}^{ox} + 5.12 eV); [b]Calculated from absorbance onset given eV = $1240/\lambda_{onset}$ + HOMO; [c]Calculated from (LUMO – HOMO); all equations are adopted from Cardona and coworkers.[7]

The electrochemical window was expanded to investigate the formation of the dication. The experimental window was adjusted to -0.4 V to 2 V vs. Ag/AgCl (Figure S18). The expanded

electrochemical window led to the formation of two new oxidation peaks and a shift in the onset of oxidation from ~ 0.5 V to ~ 0.55 V in addition to the formation of two new reduction peaks at ~ 0.4 V and ~ -0.1 V. The results are consistent with previous reports and supports the notion of the formation of new species or the degradation of our molecule.[8,9] These results support the notion of needing careful selection of experimental parameters that will be used for future studies of DHPP chromophores.

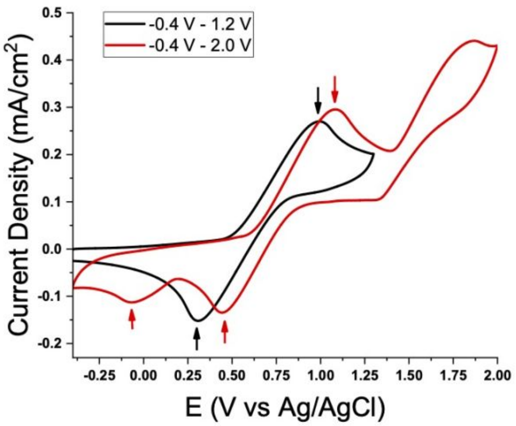

Figure S18. CV traces of 4-FDHPP demonstrating redox behavior in multiple electrochemical windows. The CV was measured from -0.4 V to 1.2 V (black trace) and -0.4 V to 2 V vs. Ag/AgCl (red trace) using a 0.5 M $TBAPF_6$/DCM supporting electrolyte.

While the absorbance profile of each DHPP is different in intensity and peak shape, all three oxidized DHPPs absorb between 400-500 nm and 600-700 nm (Figure S19 and Table S6). The λ_{max}^{α} between 400-500 nm, or the high energy transition, for 4-FDHPP displays traits of vibronic fine-structure while 3,4-F_2DHPP and 3,4,5-F_3DHPP both have broad absorbance features. The observed vibronic-fine structure may be attributed to dimerization occurring between the oxidized molecules in solution.[5,10–12] The only difference in the SOMO-β → LUMO-β is in the intensity of the peaks which also may be indicative of varying degrees of dimerization. For example, thiophene oligomers with greater charge delocalization have increased charge repulsion

resulting in less dimers being formed.[11] Here, as the fluorine substitution is increased, there is a greater push-pull effect which leads to increased charge delocalization upon photoexcitation and supports this intuition. At the same time, these results demonstrate that while increasing the electronegativity of DHPP molecules gives slight variations in the ratios between the SOMO-α → LUMO-α and SOMO-β → LUMO-β of the radical cation, the differences are not significant.

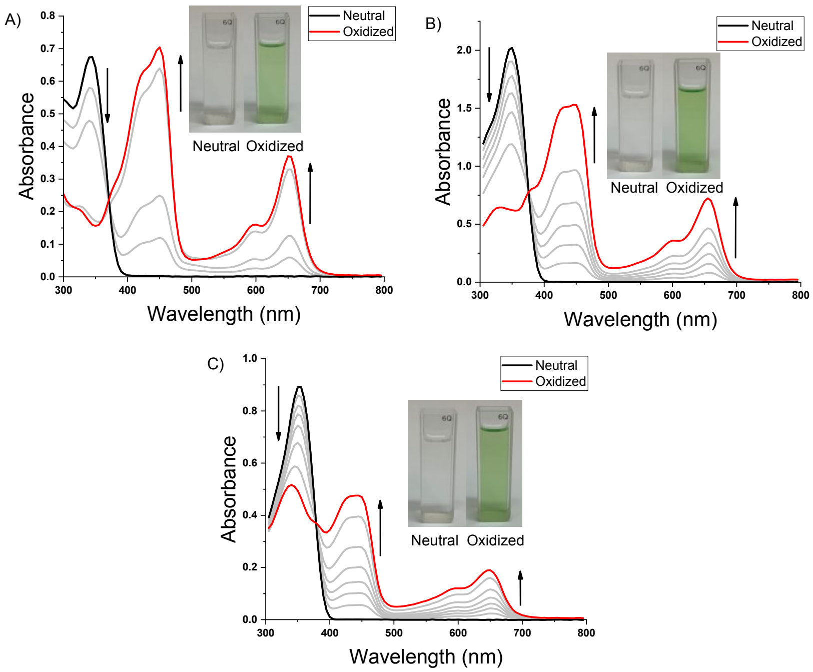

Figure S19. UV-vis solution oxidation spectra of (A) 4-FDHPP, (B) 3,4-F_2DHPP, and (C) 3,4,5-F_3DHPP in DCM using varying levels of a 0.06 mg/mL $Fe(ClO_4)_3$⍰xH_2O in ethyl acetate solution as the dopant.

Table S6. Optical data for the fluorinated DHPPs, including the absorbance λ_{max} and color coordinates for the neutral and oxidized species.

**Chromophore**	λ_{neu}^{max} **(nm)**	λ_{ox}^{max} **(nm)**		**Color Coordinates**	
		**SOMO-α**	**SOMO-β**	**Neu. ($L^*a^*b^*$)**	**Ox. ($L^*a^*b^*$)**
4-FDHPP	345	450	650	100, 0, 0	91, -27, 38
3,4-F_2DHPP	350	450	655	100, 0, 0	91, -26, 40
3,4,5-F_3DHPP	355	445	650	100, 0, 0	96, -12, 19

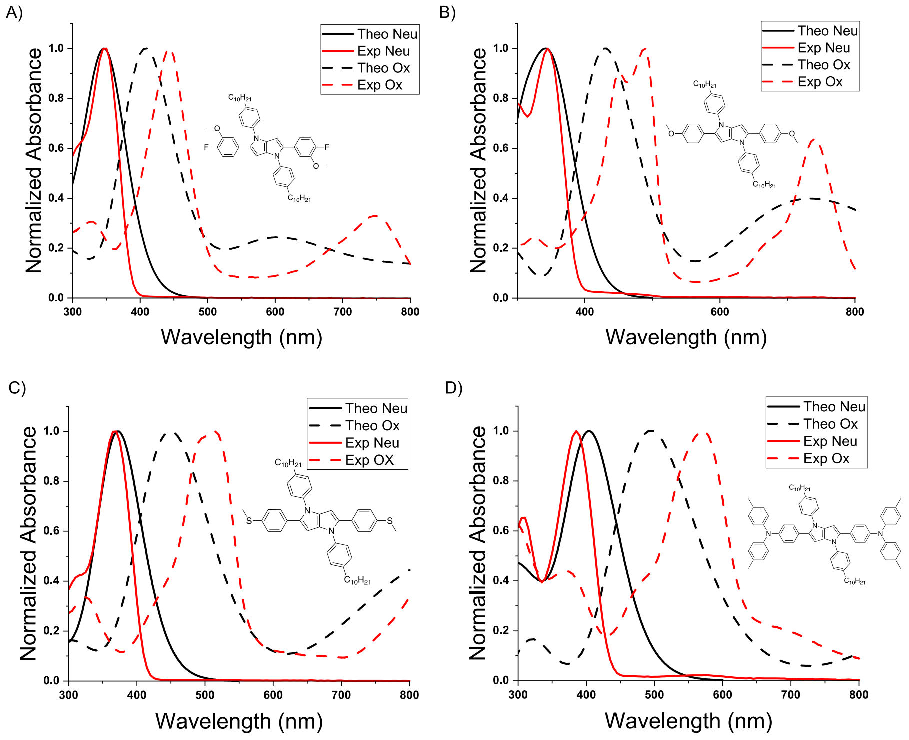

Figure S20. Comparison of calculated and experimental UV-vis absorbance spectra for the seven DHPPs (A) F,OMeDHPP, (B) 4-OMeDHPP, (C) 4-SMeDHPP, and (D) 4-tol$_2$ADHPP that confirms the level of theory is sufficient in predicting the UV-vis spectra of neutral and oxidized molecules.

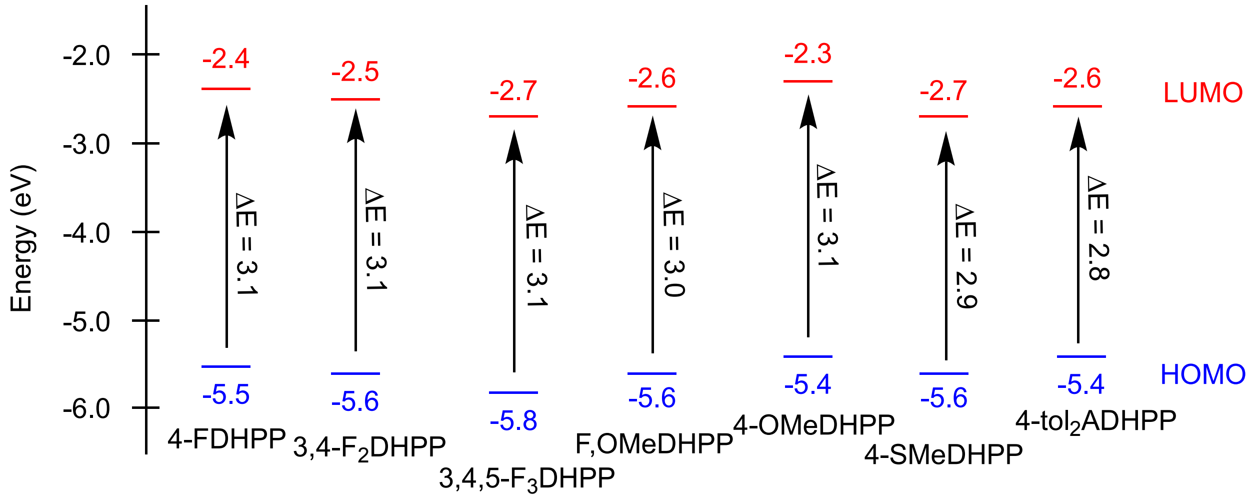

Figure S21. Energy level diagrams for DHPP chromophores characterized in this study. While the energy gap is not changing, there is a modulation of the HOMO/LUMO levels resulting from the varying choice of substituents.

DHPP DPV Results

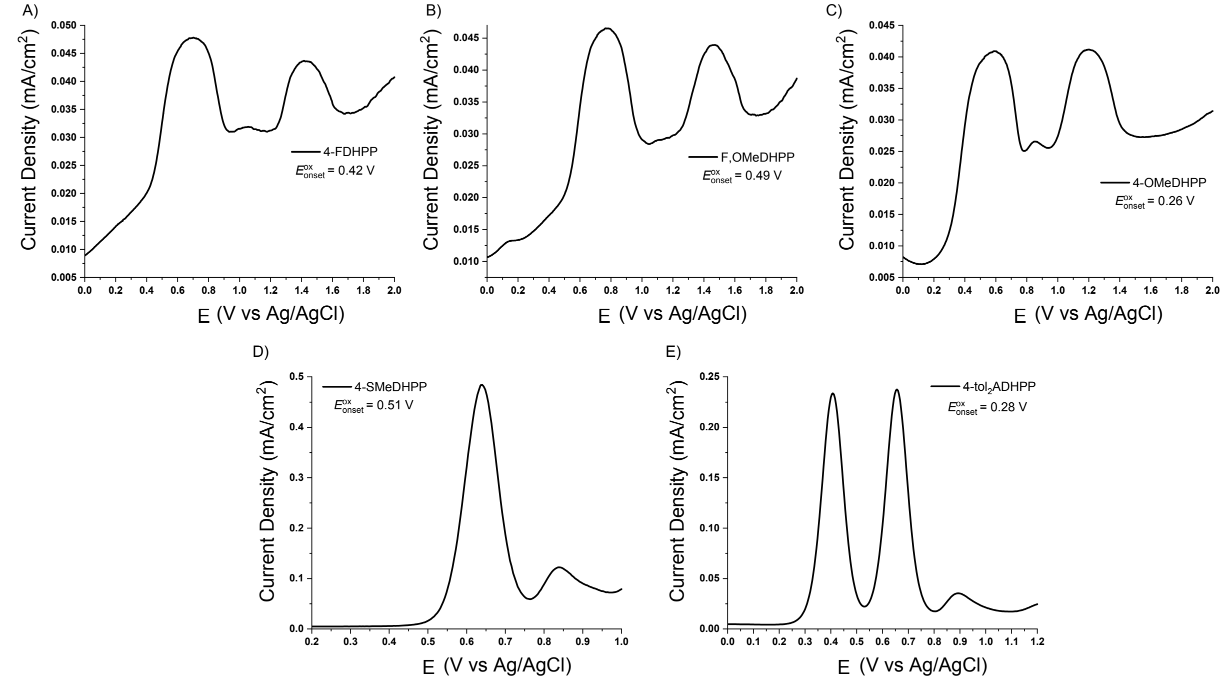

Figure S22. DPV traces of the five color-controlled DHPPs with their onsets of oxidation including A) 4-FDHPP, B) F,OMeDHPP, C) 4-OMeDHPP, D) 4-SMeDHPP, and E) 4-tol$_2$ADHPP using an Ag/AgCl reference electrode in a 0.5 M TBAPF$_6$/DCM supporting electrolyte.

Calculated Cartesian Coordinates and Simulated Absorbance Spectra

4-FDHPP

Neutral:

C	0.59389900	-0.50016700	1.12902900
C	-0.75498200	-0.15759400	1.16871600
C	-0.83578400	1.18247500	0.72124400
C	0.44320500	1.62891400	0.40491500
C	-0.98634400	3.31188600	-0.00191400
C	0.36247700	2.96920000	-0.04190100
H	-1.55766700	-0.83534100	1.42151300
H	1.16515000	3.64712000	-0.29424100
N	-1.73106000	2.21172300	0.46903800
N	1.33854700	0.59973500	0.65735100
C	-1.55925800	4.63247800	-0.29013900
C	-1.04309500	5.39424300	-1.35570400
C	-2.57520000	5.20467900	0.49989500
C	-1.51038800	6.67900900	-1.62311700
H	-0.27306900	4.96325900	-1.98852500
C	-3.06144800	6.48239600	0.23433300
H	-2.97865300	4.65067000	1.34060100
C	-2.51934500	7.20285500	-0.82382200

H	-1.11551700	7.26745600	-2.44499700
H	-3.84169500	6.92833400	0.84262700
C	-3.14433000	2.04430800	0.48366200
C	-3.75866700	1.49946500	1.61715900
C	-3.92755200	2.38391900	-0.62517700
C	-5.13737100	1.29571700	1.63389100
H	-3.15349400	1.25012000	2.48364600
C	-5.30673800	2.19174800	-0.58576500
H	-3.45505400	2.79620600	-1.51026300
C	-5.93866100	1.64093400	0.53763100
H	-5.60058700	0.87431400	2.52348300
H	-5.90367200	2.46829800	-1.45207600
C	2.75179100	0.76747100	0.64476400
C	3.53287000	0.43142800	1.75648600
C	3.36684700	1.31954200	-0.48460000
C	4.91070200	0.63284900	1.72303600
H	3.05810400	0.02397500	2.64260000
C	4.74435600	1.53261700	-0.49533600
H	2.76217700	1.57648400	-1.34921100
C	5.54376400	1.18839900	0.60237600
H	5.50463800	0.36898400	2.59539300
H	5.20665400	1.96938700	-1.37791900
C	1.16724600	-1.82028300	1.41832800
C	0.65063700	-2.58191400	2.48379700
C	2.18435000	-2.39217300	0.62955400
C	1.11857800	-3.86621300	2.75228400
H	-0.12021400	-2.15112900	3.11574800
C	2.67121900	-3.66943800	0.89619500
H	2.58816200	-1.83826900	-0.21104700
C	2.12864700	-4.38975900	1.95419400
H	0.72339000	-4.45450100	3.57413000
H	3.45227100	-4.11519100	0.28878900
F	2.59673900	-5.63035400	2.21405800
F	-2.98677600	8.44388500	-1.08263400
C	-7.43082900	1.38921900	0.55000900
H	-7.93790600	2.15088800	-0.05712600
H	-7.81492900	1.50156500	1.57277400
C	-7.81478300	-0.00851500	0.02254300
H	-7.42768100	-0.12499000	-0.99938900
H	-7.30693900	-0.77215500	0.62821400
C	-9.32739900	-0.26126700	0.03430700
H	-9.70888300	-0.13604800	1.05872500
H	-9.82910400	0.50802700	-0.57152200
C	-9.71840300	-1.65077500	-0.48567900
H	-9.33596100	-1.77612100	-1.50949900
H	-9.21710500	-2.41951100	0.12093000

C	-11.23159700	-1.90404900	-0.47529800
H	-11.61380400	-1.77631300	0.54849500
H	-11.73225400	-1.13567300	-1.08311600
C	-11.62469700	-3.29396600	-0.99236700
H	-11.24224000	-3.42208100	-2.01597000
H	-11.12438500	-4.06225900	-0.38418800
C	-13.13799400	-3.54625800	-0.98202800
H	-13.52036900	-3.41665900	0.04151500
H	-13.63800400	-2.77836800	-1.59108400
C	-13.53254900	-4.93634800	-1.49700000
H	-13.15094700	-5.06695700	-2.52079000
H	-13.03340800	-5.70504300	-0.88807100
C	-15.04581600	-5.18845500	-1.48658000
H	-15.42717800	-5.05818500	-0.46369700
H	-15.54456900	-4.42122400	-2.09591100
C	-15.43124600	-6.57901700	-2.00151700
H	-16.51701400	-6.72752600	-1.98117100
H	-15.09432400	-6.72647400	-3.03510500
H	-14.97568800	-7.36826400	-1.39068000
C	7.04503200	1.37494300	0.56635500
H	7.41181700	1.60393900	1.57587500
H	7.29371700	2.24313700	-0.05841800
C	7.79701900	0.13983200	0.02945800
H	7.42987600	-0.09249600	-0.98010900
H	7.29371700	2.24313700	-0.05841800
C	7.79701900	0.13983200	0.02945800
H	7.42987600	-0.09249600	-0.98010900
H	7.54639700	-0.73079200	0.65157600
C	9.31850400	0.32931300	-0.00517500
H	9.56173700	1.20522800	-0.62501300
H	9.67836700	0.56570600	1.00731600
C	10.07401400	-0.89481800	-0.53876200
H	9.71352100	-1.13093500	-1.55097900
H	9.83039900	-1.77026300	0.08127000
C	11.59630600	-0.70675500	-0.57418000
H	11.83938700	0.16929400	-1.19383800
H	11.95633600	-0.47046000	0.43825600
C	12.35314300	-1.92992400	-1.10787100
H	11.99296700	-2.16626400	-2.12025100
H	12.11005700	-2.80590300	-0.48818700
C	13.87534100	-1.74161300	-1.14331400
H	14.11821500	-0.86534900	-1.76283400
H	14.23534000	-1.50526700	-0.13081700
C	14.63303400	-2.96403300	-1.67701000
H	14.27396100	-3.20093800	-2.68982200
H	14.39108200	-3.84087100	-1.05779500

C	16.15520300	-2.77598200	-1.71253000
H	16.39717800	-1.90029700	-2.33165500
H	16.51427000	-2.54014100	-0.70053900
C	16.90396400	-4.00121400	-2.24689700
H	16.59194000	-4.24100100	-3.27096700
H	17.98731600	-3.83523800	-2.25945300
H	16.70975100	-4.88570700	-1.62761500

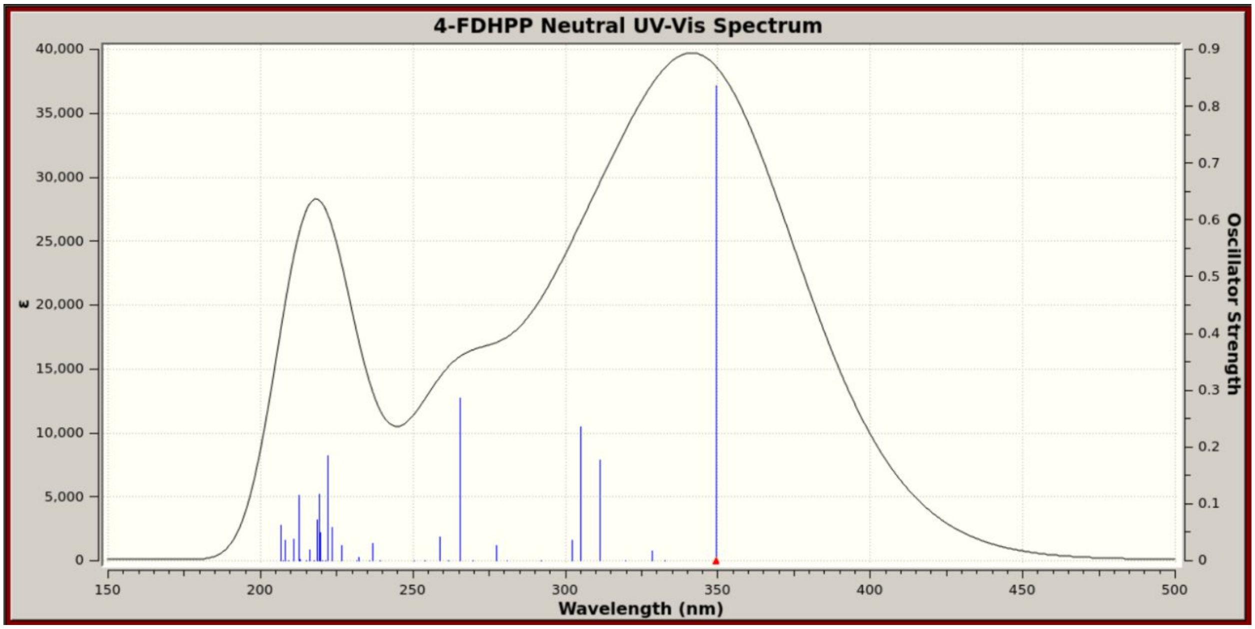

Radical Cation:

C	0.57700000	-0.44809500	0.68214400
C	-0.80674800	-0.09681600	0.76095200
C	-0.87393900	1.25022700	0.46685400
C	0.43914700	1.73421600	0.20922000
C	-1.01214400	3.43247600	-0.00609900
C	0.37175400	3.08120900	-0.08490700
H	-1.61208000	-0.79045400	0.94980200
H	1.17696200	3.77495900	-0.27388700
N	-1.76023000	2.30259500	0.32583000
N	1.32532900	0.68161600	0.35028600
C	-1.55076100	4.76676000	-0.18764000
C	-0.84606500	5.67966900	-1.00833100
C	-2.72489300	5.21445500	0.46217800
C	-1.29509100	6.97910000	-1.18648100
H	0.04554900	5.35445800	-1.53400600
C	-3.17454800	6.51646900	0.29866500
H	-3.27091600	4.55001600	1.11998900
C	-2.45680100	7.38061700	-0.52758500
H	-0.77015300	7.68118300	-1.82489300
H	-4.06423200	6.87609900	0.80395400

C	-3.18962200	2.12904300	0.32766900
C	-3.83062700	1.75821700	1.51207200
C	-3.91981300	2.28986200	-0.85191000
C	-5.20984800	1.55366100	1.50937900
H	-3.25630300	1.64584800	2.42737100
C	-5.29887900	2.09327000	-0.83326700
H	-3.41368200	2.56879600	-1.77109000
C	-5.96898300	1.71993400	0.34176300
H	-5.70627800	1.27087700	2.43407200
H	-5.86536500	2.22723200	-1.75115600
C	2.75483600	0.85388900	0.35194900
C	3.48143200	0.69252600	1.53383200
C	3.39838600	1.23274700	-0.82839600
C	4.85961500	0.89463500	1.52089500
H	2.97178900	0.41702700	2.45210200
C	4.77683000	1.44308700	-0.81991500
H	2.82564100	1.35498700	-1.74342500
C	5.53267100	1.27372400	0.34927600
H	5.42195300	0.76925700	2.44254800
H	5.27385200	1.74119800	-1.73944600
C	1.11566100	-1.78253200	0.86306500
C	0.41163200	-2.69560100	1.68403500
C	2.28909000	-2.23006100	0.21196400
C	0.86063200	-3.99521700	1.86120400
H	-0.47943700	-2.37040900	2.21066000
C	2.73869100	-3.53224400	0.37447700
H	2.83464800	-1.56527400	-0.44590700
C	2.02160600	-4.39662600	1.20101400
H	0.33621600	-4.69747600	2.49985400
H	3.62783800	-3.89178400	-0.13182000
F	2.45867300	-5.64808600	1.36365500
F	-2.89386300	8.63186000	-0.69119300
C	-7.45874700	1.46254800	0.33949000
H	-7.94345300	2.11763600	-0.39531100
H	-7.87816600	1.72602100	1.31871200
C	-7.81316000	-0.00544600	0.01770800
H	-7.39081400	-0.27109200	-0.96151700
H	-7.32310900	-0.66228400	0.75016500
C	-9.32419300	-0.26819600	0.01790900
H	-9.74003400	0.00496500	0.99878100
H	-9.80832000	0.39568100	-0.71314400
C	-9.68571400	-1.72506700	-0.30017500
H	-9.26991500	-1.99627800	-1.28185900
H	-9.19803100	-2.38780200	0.43008200
C	-11.19649200	-1.99299800	-0.29771600
H	-11.61143400	-1.72099100	0.68404900

H	-11.68378500	-1.32947300	-1.02726500
C	-11.55906200	-3.44948700	-0.61560100
H	-11.14452100	-3.72054300	-1.59799900
H	-11.06972500	-4.11263000	0.11331200
C	-13.06937300	-3.71921600	-0.61124900
H	-13.48344000	-3.44790800	0.37122700
H	-13.55850300	-3.05563500	-1.33973400
C	-13.43249100	-5.17536100	-0.92917800
H	-13.01931400	-5.44705800	-1.91215600
H	-12.94314100	-5.83962500	-0.20107700
C	-14.94256600	-5.44620400	-0.92408500
H	-15.35526000	-5.17543800	0.05818100
H	-15.43139900	-4.78290600	-1.65177700
C	-15.29620600	-6.90241200	-1.24264300
H	-16.38015400	-7.06246000	-1.23109300
H	-14.92753200	-7.19242400	-2.23429300
H	-14.85076300	-7.58803000	-0.51124600
C	7.03352300	1.45493200	0.33927900
H	7.36441800	1.82852100	1.31653000
H	7.30626700	2.22087700	-0.39760100
C	7.79259500	0.15004100	0.01489500
H	7.45967900	-0.22581300	-0.96286000
H	7.51497000	-0.61933300	0.74908300
C	9.31553300	0.33129900	0.00752000
H	9.58524800	1.10763500	-0.72334800
H	9.64070100	0.71004500	0.98750300
C	10.07758200	-0.96021200	-0.31750600
H	9.75128100	-1.33710300	-1.29818300
H	9.80460200	-1.73642800	0.41271000
C	11.60176600	-0.78394900	-0.32376700
H	11.87393200	-0.00582900	-1.05210700
H	11.92745900	-0.40821600	0.65742200
C	12.36436300	-2.07427000	-0.65148000
H	12.03826800	-2.44904300	-1.63313600
H	12.09049600	-2.85254600	0.07634500
C	13.88862000	-1.89986000	-0.65683000
H	14.16202500	-1.12047200	-1.38356000
H	14.21443500	-1.52598300	0.32519100
C	14.65143600	-3.18938000	-0.98630600
H	14.32629900	-3.56341000	-1.96873400
H	14.37821000	-3.96943500	-0.25991200
C	16.17579100	-3.01612900	-0.99141900
H	16.44877400	-2.23689400	-1.71720900
H	16.50072000	-2.64340700	-0.00959300
C	16.92949600	-4.30831500	-1.32197400
H	16.65208900	-4.68684100	-2.31351600

H	18.01391500	-4.15109200	-1.31758100
H	16.70453700	-5.09646400	-0.59285400

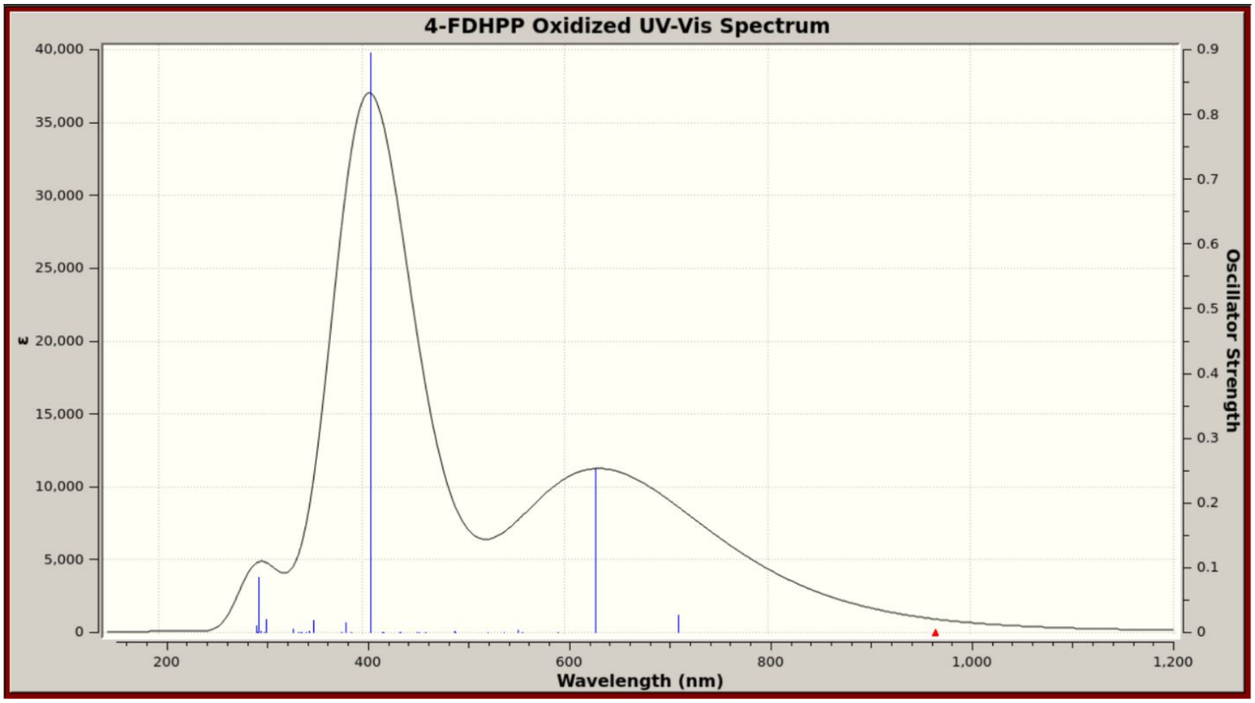

F,OMeDHPP

Neutral:

C	-1.99424900	-0.72673000	-0.26377700
C	-0.85214300	-1.52319200	-0.27451500
C	0.24133200	-0.64850900	-0.07204700
C	-0.24133100	0.64850900	0.07204600
C	1.99425000	0.72673000	0.26377800
C	0.85214300	1.52319100	0.27451600
H	-0.85120000	-2.60118400	-0.34750700
H	0.85120100	2.60118400	0.34750600
N	1.62271100	-0.61552700	0.04940600
N	-1.62271000	0.61552700	-0.04940500
C	3.38247200	1.19652300	0.34882100
C	3.70079900	2.26050900	1.21391000
C	4.40125800	0.66787800	-0.46057600
C	4.99650700	2.76510600	1.26534000
H	2.93290800	2.67597900	1.85823000
C	5.71077900	1.15378600	-0.41095100
H	4.19654800	-0.12910700	-1.16619900
C	5.98997700	2.20514100	0.47343100
H	5.26451800	3.57468400	1.93701400
C	2.42917200	-1.78298800	0.16236600
C	2.32027700	-2.78827800	-0.80386400

C	3.29751800	-1.96341700	1.24532100
C	3.07108300	-3.95715200	-0.68353100
H	1.65133400	-2.64768100	-1.64757800
C	4.05446000	-3.12793100	1.34174000
H	3.37012000	-1.19594700	2.00844800
C	3.95756000	-4.14650100	0.38320800
H	2.97183300	-4.73107000	-1.44120800
H	4.72548500	-3.25305000	2.18857300
C	-2.42917200	1.78298900	-0.16236400
C	-3.29751700	1.96341800	-1.24531900
C	-2.32027600	2.78827700	0.80386700
C	-4.05445800	3.12793200	-1.34173800
H	-3.37011800	1.19594800	-2.00844700
C	-3.07108300	3.95715100	0.68353500
H	-1.65133300	2.64768000	1.64758200
C	-3.95755900	4.14650100	-0.38320500
H	-4.72548300	3.25305200	-2.18857100
H	-2.97183300	4.73106900	1.44121200
C	-3.38247200	-1.19652300	-0.34882100
C	-3.70079800	-2.26050900	-1.21391000
C	-4.40125800	-0.66787800	0.46057500
C	-4.99650600	-2.76510500	-1.26534200
H	-2.93290600	-2.67597900	-1.85823000
C	-5.71078000	-1.15378600	0.41094900
H	-4.19655000	0.12910700	1.16619900
C	-5.98997700	-2.20514000	-0.47343400
F	-7.25436600	-2.68758600	-0.57912300
F	7.25436600	2.68758700	0.57911900
C	4.80063700	-5.39568100	0.48925300
H	5.81203600	-5.22853100	0.09521400
H	4.36271600	-6.22420800	-0.07699900
C	-4.80063800	5.39568000	-0.48924700
H	-4.90988900	5.71877600	-1.53052900
H	-4.36273900	6.22419600	0.07703800
O	6.62773700	0.53596700	-1.21161400
C	7.67448500	1.31172200	-1.80008300
H	8.47449100	1.52339600	-1.08608700
H	8.06403400	0.70191500	-2.61838200
H	7.28907200	2.25602300	-2.20282500
H	4.90992000	-5.71875300	1.53053900
H	-5.81204900	5.22851600	-0.09524500
O	-6.62773800	-0.53596800	1.21161000
C	-7.67448800	-1.31172200	1.80007700
H	-8.06403300	-0.70191900	2.61838000
H	-8.47449600	-1.52338800	1.08608000
H	-7.28907900	-2.25602700	2.20281200

H	-5.26451700	-3.57468300	-1.93701600

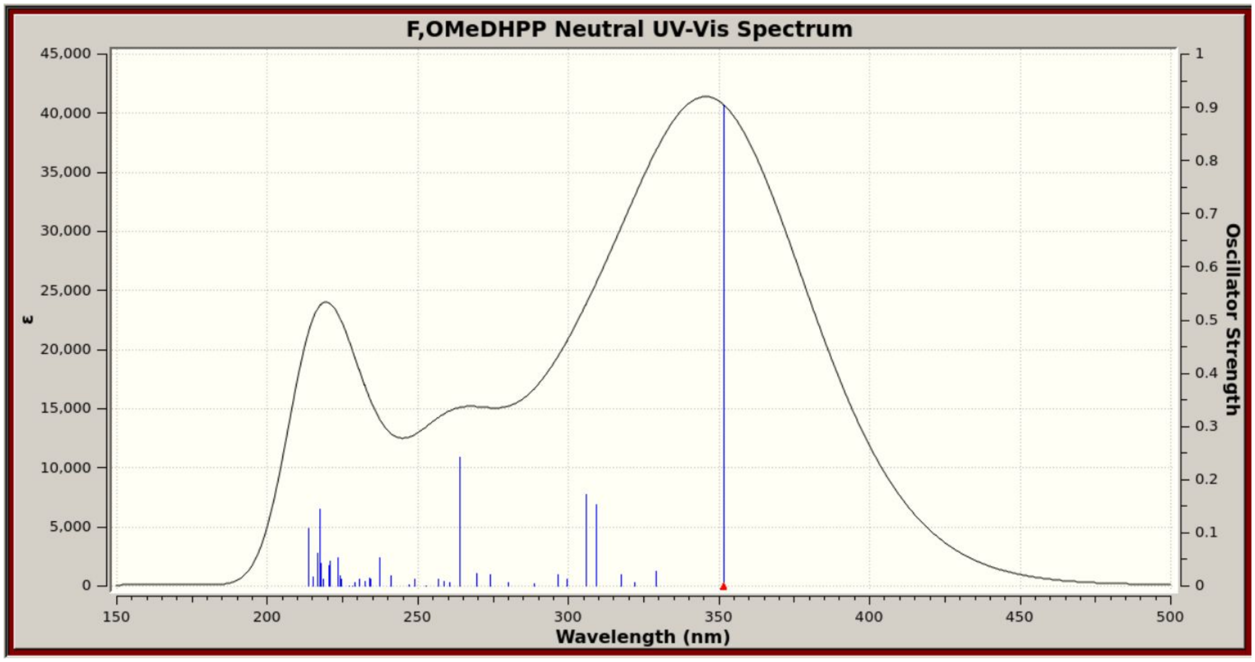

Radical Cation:

C	-2.10541600	-0.25602400	0.11504100
C	-1.14957400	-1.31489200	0.03604700
C	0.08834700	-0.70565200	-0.02055700
C	-0.08743800	0.70558600	0.02232500
C	2.10634300	0.25593800	-0.11366300
C	1.15044400	1.31481400	-0.03456700
H	-1.38861400	-2.36668300	0.08002300
H	1.38942300	2.36661000	-0.07878100
N	1.44354100	-0.97190300	-0.09812700
N	-1.44267100	0.97181300	0.09988500
C	3.54084600	0.42830700	-0.25148400
C	4.12871800	1.59659500	0.27168100
C	4.35792800	-0.49967900	-0.93675900
C	5.49153800	1.87144100	0.13671200
H	3.53982700	2.31629200	0.82927200
C	5.71007000	-0.23582300	-1.09013100
H	3.93633900	-1.39503100	-1.37413800
C	6.26754600	0.92745300	-0.56632600
C	1.99077300	-2.29607900	0.03658900
C	1.74100400	-3.24261100	-0.95869600
C	2.72644100	-2.64242700	1.17253100
C	2.23552000	-4.53877000	-0.81272900
H	1.17689700	-2.96325800	-1.84400300
C	3.22322400	-3.93736400	1.29642900
H	2.90879500	-1.90457900	1.94775500

C	2.98416100	-4.90922400	0.31252400
H	2.04184200	-5.27098700	-1.59199100
H	3.80054400	-4.20055900	2.17884700
C	-1.99040100	2.29571200	-0.03567700
C	-2.72271600	2.64205200	-1.17410500
C	-1.73708700	3.24428900	0.95643800
C	-3.21343700	3.93858200	-1.30268400
H	-2.90075200	1.90463800	-1.95074600
C	-2.22575700	4.54237300	0.80577500
H	-1.16873500	2.96739800	1.83980300
C	-2.97579800	4.91115900	-0.31877300
H	-3.78205800	4.20370300	-2.19020400
H	-2.02369500	5.27799600	1.57962600
C	-3.54000200	-0.42822500	0.25242600
C	-4.12809900	-1.59591800	-0.27174000
C	-4.35685500	0.49944100	0.93833700
C	-5.49102200	-1.87047500	-0.13712800
H	-3.53931100	-2.31530800	-0.82984400
C	-5.70909500	0.23583500	1.09139400
H	-3.93488300	1.39425000	1.37648700
C	-6.26682900	-0.92687100	0.56661700
H	-6.35990100	0.91443100	1.63272000
F	-7.58132300	-1.13516900	0.75785300
F	7.58197600	1.13587300	-0.75775800
C	3.49981200	-6.31843000	0.47657700
H	4.49099000	-6.33065600	0.94136700
H	3.56774300	-6.83568100	-0.48505000
C	-3.52932400	6.30793000	-0.46477000
H	-3.44593800	6.66484900	-1.49663800
H	-3.00695400	7.01696400	0.18405300
O	-5.91610400	-3.02918400	-0.68353300
C	-7.31289800	-3.31575700	-0.86352300
H	-7.33279400	-4.23122200	-1.45547100
H	-7.81150700	-3.48264000	0.09381500
H	-7.81425100	-2.51198700	-1.41027000
H	2.83287700	-6.90668900	1.11994800
H	-4.59328200	6.33819400	-0.19701500
O	5.91629600	3.03065500	0.68227400
C	7.31303400	3.31864600	0.86039100
H	7.81042600	3.48491400	-0.09769100
H	7.33276200	4.23479200	1.45128900
H	7.81571200	2.51593700	1.40747000
H	6.36100300	-0.91478000	-1.63084800

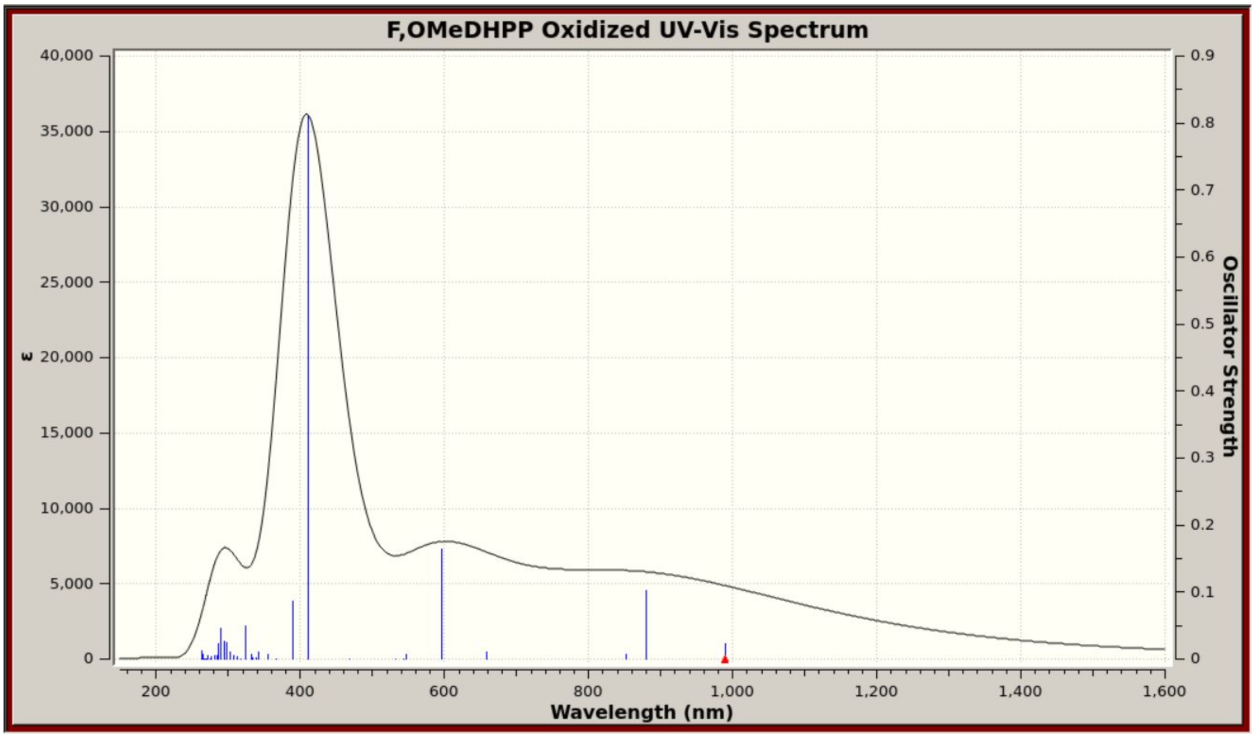

3,4-F_2DHPP

Neutral:

C	-1.96135100	-0.83902700	-0.13659700
C	-0.77949800	-1.57464100	-0.18382000
C	0.27253900	-0.63707900	-0.06533200
C	-0.27253700	0.63707900	0.06532900
C	1.96135300	0.83902700	0.13659500
C	0.77950000	1.57464200	0.18381700
H	-0.72245000	-2.65284700	-0.22387900
H	0.72245200	2.65284700	0.22387700
N	1.65379900	-0.52707100	-0.01840000
N	-1.65379700	0.52707200	0.01839700
C	3.32368800	1.38270900	0.13319300
C	3.62805400	2.49843100	0.93520100
C	4.33585500	0.86188800	-0.69697200
C	4.89274900	3.08461500	0.90861800
H	2.86708500	2.89913300	1.59683900
C	5.59371100	1.44111200	-0.70534200
H	4.15291200	0.01858600	-1.35241700
C	5.87763500	2.55100600	0.08946200
H	5.13262700	3.94327000	1.52744700
C	2.52897300	-1.64581500	0.08822900
C	2.45011100	-2.67161100	-0.85906400
C	3.43769800	-1.75632200	1.14692800
C	3.27191800	-3.79233000	-0.74409900
H	1.74862800	-2.58468100	-1.68345500

C	4.26476200	-2.87284000	1.23783500
H	3.48642300	-0.97333600	1.89633200
C	4.19950400	-3.91119800	0.29792400
H	3.19596100	-4.58364000	-1.48620300
H	4.96632900	-2.94482000	2.06578800
C	-2.52897200	1.64581500	-0.08822800
C	-3.43770000	1.75632200	-1.14692400
C	-2.45010900	2.67161000	0.85906600
C	-4.26476600	2.87284000	-1.23782800
H	-3.48642600	0.97333800	-1.89633000
C	-3.27191800	3.79232800	0.74410500
H	-1.74862400	2.58468000	1.68345600
C	-4.19950700	3.91119600	-0.29791500
H	-4.96633500	2.94482000	-2.06577900
H	-3.19596000	4.58363800	1.48621000
C	-3.32368700	-1.38270800	-0.13319600
C	-3.62805400	-2.49842800	-0.93520700
C	-4.33585400	-0.86188900	0.69697100
C	-4.89274900	-3.08461100	-0.90862600
H	-2.86708500	-2.89912900	-1.59684600
C	-5.59371000	-1.44111200	0.70533900
H	-4.15291000	-0.01859000	1.35241900
C	-5.87763400	-2.55100400	-0.08946800
H	-5.13262700	-3.94326400	-1.52745700
F	-7.11049800	-3.08816300	-0.05378500
F	7.11049700	3.08816600	0.05377800
C	5.11730700	-5.10706800	0.39664300
H	6.09948400	-4.89252600	-0.04528800
H	4.70489200	-5.97361400	-0.13032500
C	-5.11731300	5.10706400	-0.39662900
H	-5.28904200	5.39566100	-1.43943500
H	-4.70488600	5.97361700	0.13031800
F	-6.56032000	-0.94731700	1.50077100
F	6.56032100	0.94731500	-1.50077200
H	-6.09947900	4.89252700	0.04532900
H	5.28901000	-5.39567900	1.43945000

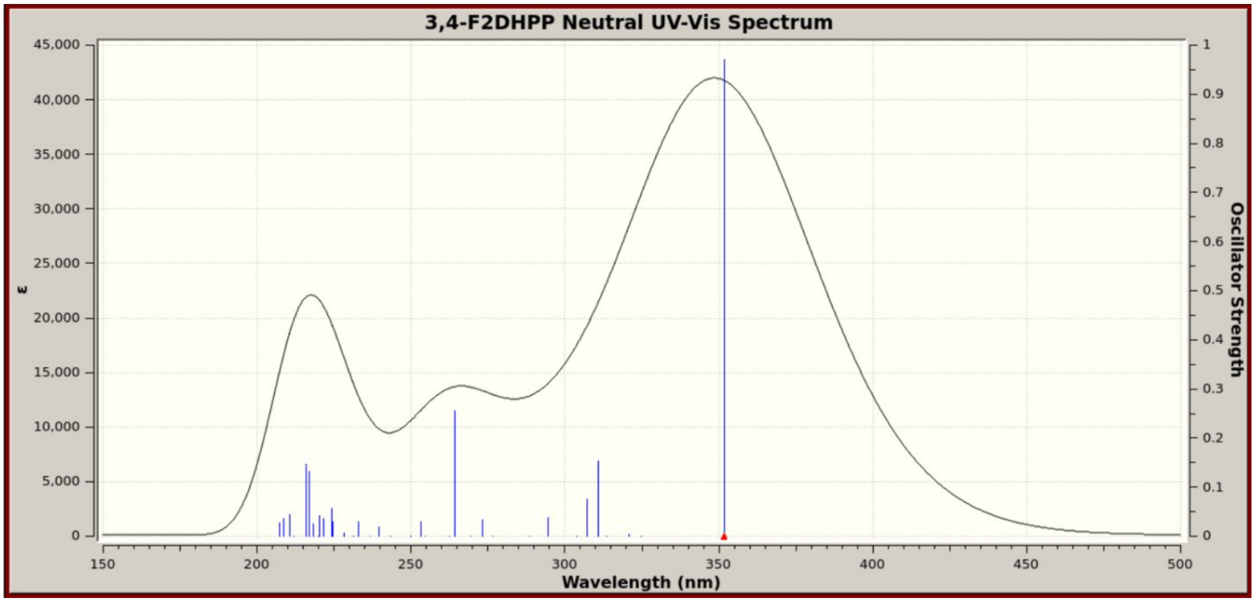

Radical Cation:

C	-1.94593200	-0.84531900	-0.08423400
C	-0.72235100	-1.58371200	-0.14016700
C	0.28859300	-0.64823300	-0.05367900
C	-0.28781900	0.64830400	0.05397800
C	1.94672500	0.84544900	0.08411900
C	0.72309800	1.58381200	0.14019100
H	-0.64862800	-2.66005100	-0.17671200
H	0.64932800	2.66016100	0.17639600
N	1.66512500	-0.51526200	-0.02865000
N	-1.66435200	0.51533700	0.02890900
C	3.27636000	1.42642600	0.08730000
C	3.46829500	2.68402500	0.70472200
C	4.37812800	0.80460800	-0.54385200
C	4.71386300	3.29900200	0.70538400
H	2.64300900	3.16828300	1.21448100
C	5.60930900	1.42964700	-0.54627400
H	4.28644000	-0.14401300	-1.05648000
C	5.78475700	2.67147500	0.07770300
H	4.87492100	4.25689900	1.18788900
C	2.56111000	-1.63906500	0.06783900
C	2.69524100	-2.50158100	-1.02128800
C	3.25750100	-1.88260300	1.25450300
C	3.53781600	-3.60901700	-0.91859100
H	2.15743600	-2.30042200	-1.94347200
C	4.10260700	-2.98593200	1.33567700
H	3.13951400	-1.21374900	2.10165300
C	4.25640900	-3.86995700	0.25576200
H	3.64391700	-4.27515900	-1.77038000

H	4.64806400	-3.16825200	2.25785400
C	-2.56066600	1.63892500	-0.06739600
C	-3.25257600	1.88583300	-1.25554100
C	-2.69327000	2.50249900	1.02157800
C	-4.09302800	2.99309600	-1.33856600
H	-3.13005900	1.22062100	-2.10490800
C	-3.53074200	3.61310200	0.91717900
H	-2.15283300	2.30278800	1.94254900
C	-4.24956600	3.87449900	-0.25750500
H	-4.63012700	3.18180500	-2.26426500
H	-3.63092600	4.28323600	1.76663100
C	-3.27566900	-1.42609300	-0.08824400
C	-3.46773500	-2.68318400	-0.70660100
C	-4.37746800	-0.80440200	0.54291600
C	-4.71351400	-3.29776400	-0.70819400
H	-2.64239400	-3.16731700	-1.21639600
C	-5.60886200	-1.42901400	0.54435600
H	-4.28555600	0.14379800	1.05627400
C	-5.78447900	-2.67033000	-0.08054900
H	-4.87468700	-4.25526100	-1.19145700
F	-6.98805700	-3.23641900	-0.06240700
F	6.98812100	3.23795600	0.05865000
C	5.15777000	-5.07518100	0.36927600
H	6.10965500	-4.81784700	0.84578500
H	5.37458100	-5.50851100	-0.61119600
C	-5.18214400	5.05720100	-0.35358600
H	-5.16754900	5.49783700	-1.35558000
H	-4.91774400	5.83703900	0.36658500
F	-6.65410800	-0.86133500	1.15212400
F	6.65447100	0.86199000	-1.15408200
H	-6.21700900	4.75582400	-0.14647700
H	4.69083400	-5.85705300	0.98153600

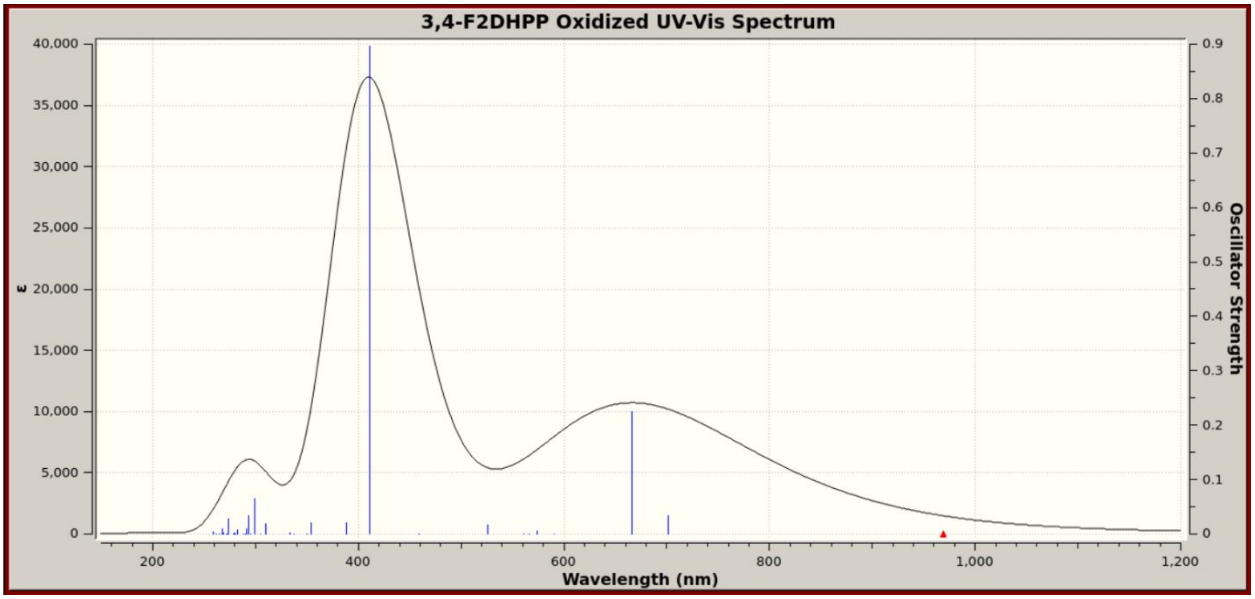

3,4,5-F_3DHPP

Neutral:

C	-2.03980300	-0.62900700	-0.04695700
C	-0.94427300	-1.48912800	-0.08633200
C	0.20284600	-0.66489300	-0.04015500
C	-0.20131900	0.66556100	0.03973700
C	2.04137000	0.62959800	0.04632100
C	0.94585600	1.48975000	0.08571800
H	-1.00184000	-2.56798900	-0.07481000
H	1.00352500	2.56860200	0.07387400
N	1.58805900	-0.70134800	-0.03303900
N	-1.58654800	0.70194700	0.03271200
C	3.45145000	1.02417400	-0.02123500
C	3.88067800	2.15301800	0.70230700
C	4.38211000	0.34747000	-0.83233700
C	5.19338900	2.58725000	0.60049600
H	3.20341200	2.68708800	1.35882500
C	5.69237100	0.79514700	-0.90176200
H	4.09865500	-0.51210900	-1.42689900
C	6.11995500	1.91681900	-0.19447200
C	2.34177100	-1.90222600	0.11486200
C	2.14775200	-2.94974900	-0.79010600
C	3.24653400	-2.06270800	1.17073600
C	2.85142700	-4.14427800	-0.63506800
H	1.45554100	-2.82094900	-1.61688700
C	3.95502400	-3.25377800	1.30155200
H	3.38871200	-1.25680000	1.88322500
C	3.76874000	-4.31857800	0.40765000
H	2.69113300	-4.95012000	-1.34730900

H	4.65938700	-3.36225300	2.12313700
C	-2.34102000	1.90233800	-0.11524200
C	-3.24295000	2.06363200	-1.17287000
C	-2.14480400	2.95157900	0.78790100
C	-3.94861500	3.25658000	-1.30649700
H	-3.38039700	1.25990300	-1.88873600
C	-2.84520300	4.14714800	0.63022000
H	-1.44809000	2.82513800	1.61127400
C	-3.76503200	4.32002400	-0.41122600
H	-4.64643200	3.36828800	-2.13313300
H	-2.67891400	4.95607400	1.33766500
C	-3.44992000	-1.02348300	0.02011100
C	-3.87903700	-2.15227600	-0.70357600
C	-4.38080800	-0.34670200	0.83088700
C	-5.19184200	-2.58633900	-0.60225400
H	-3.20161000	-2.68641900	-1.35987000
C	-5.69114700	-0.79418800	0.89980200
H	-4.09741900	0.51279600	1.42558800
C	-6.11863500	-1.91578900	0.19235700
F	-7.38861100	-2.33569100	0.27142100
F	7.38983800	2.33690200	-0.27401800
C	4.52247600	-5.61626500	0.58129700
H	5.58240900	-5.43875500	0.79589800
H	4.46035400	-6.23823700	-0.31718500
C	-4.55221700	5.60124100	-0.55554800
H	-4.70660700	5.86028900	-1.60869200
H	-4.04331300	6.44118000	-0.07174400
F	-6.58276000	-0.15546100	1.67621800
F	6.58377700	0.15660500	-1.67849300
H	-5.54533700	5.51158200	-0.09555600
H	4.11826900	-6.20299300	1.41665200
F	-5.60464900	-3.66149400	-1.29484400
F	5.60631800	3.66245100	1.29294500

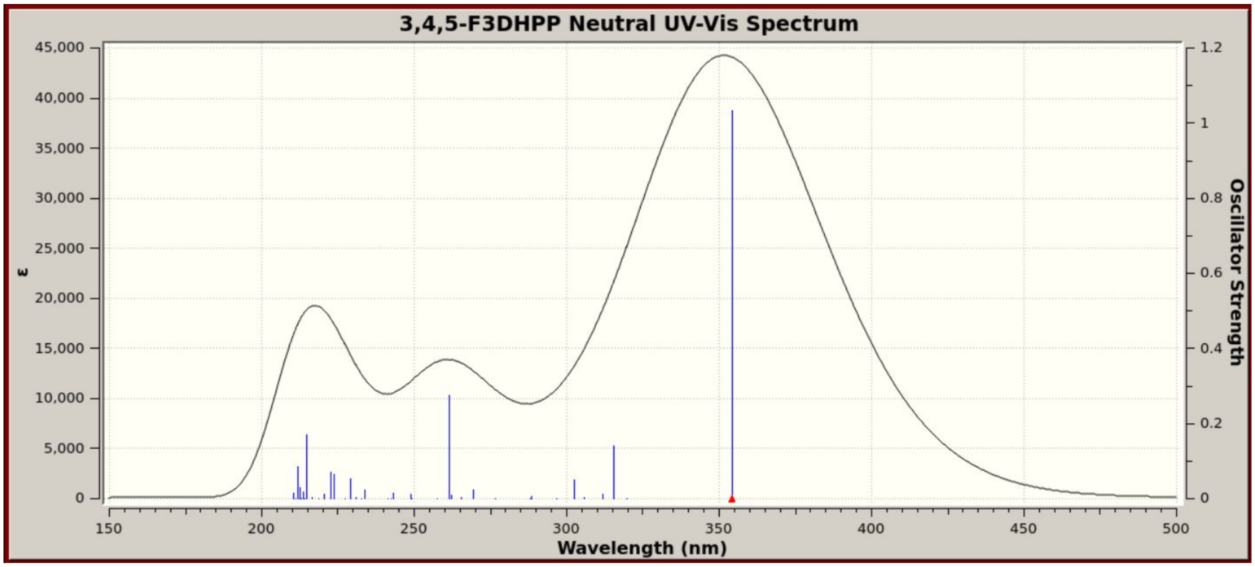

Radical Cation:

C	-2.02896200	-0.62009400	-0.01471700
C	-0.89863000	-1.49596200	-0.05596300
C	0.21261500	-0.67876700	-0.02791600
C	-0.21188100	0.67870000	0.02935400
C	2.02971500	0.62006100	0.01567200
C	0.89933300	1.49591300	0.05711700
H	-0.94831700	-2.57433700	-0.04622300
H	0.94897100	2.57428900	0.04707300
N	1.59563600	-0.70257900	-0.03049900
N	-1.59491100	0.70251300	0.03186900
C	3.41702300	1.04667000	-0.02775800
C	3.75080200	2.30089800	0.53172400
C	4.42602400	0.27993400	-0.64997700
C	5.05474600	2.75851100	0.47259700
H	3.01605600	2.91219100	1.04176300
C	5.71996500	0.76732100	-0.70321000
H	4.21950300	-0.67343200	-1.11737500
C	6.05659100	2.00364500	-0.14408500
C	2.36133600	-1.91677000	0.09855800
C	2.38302500	-2.82636400	-0.96010900
C	3.04127600	-2.19464900	1.28692600
C	3.09751500	-4.01696600	-0.82482500
H	1.85884700	-2.59877200	-1.88401800
C	3.75810200	-3.38288500	1.40050100
H	3.01036300	-1.48745600	2.11031600
C	3.79735400	-4.31591300	0.35217800
H	3.11703900	-4.72032600	-1.65272100
H	4.29172600	-3.59297300	2.32361100
C	-2.36093400	1.91649000	-0.09763900

C	-3.03552200	2.19673000	-1.28830900
C	-2.38107000	2.82750100	0.96008000
C	-3.74664000	3.38831400	-1.40500300
H	-2.99925800	1.49215400	-2.11371900
C	-3.08971700	4.02100800	0.82180300
H	-1.85433800	2.60164200	1.88296800
C	-3.78919100	4.31980800	-0.35573400
H	-4.27041000	3.60348400	-2.33253000
H	-3.10268300	4.72828900	1.64652200
C	-3.41637200	-1.04650100	0.02780900
C	-3.75021000	-2.30035500	-0.53241700
C	-4.42546900	-0.27974900	0.64978800
C	-5.05435100	-2.75757100	-0.47425800
H	-3.01535900	-2.91163000	-1.04233000
C	-5.71961200	-0.76669700	0.70197200
H	-4.21876800	0.67328800	1.11776900
C	-6.05633400	-2.00264200	0.14211600
F	-7.30290800	-2.45061200	0.19525800
F	7.30296100	2.45202300	-0.19819800
C	4.55592200	-5.61201800	0.50135500
H	5.51125200	-5.45899400	1.01390600
H	4.75988100	-6.07220800	-0.46966200
C	-4.58306000	5.59640100	-0.48795900
H	-4.52547000	5.99973800	-1.50399600
H	-4.22776500	6.36428400	0.20534900
F	-6.68133600	-0.06192300	1.30070100
F	6.68154800	0.06271800	-1.30223800
H	-5.64388200	5.42034200	-0.26820500
H	3.98175800	-6.33443300	1.09541400
F	-5.38507800	-3.93169400	-1.01413900
F	5.38541900	3.93296700	1.01178000

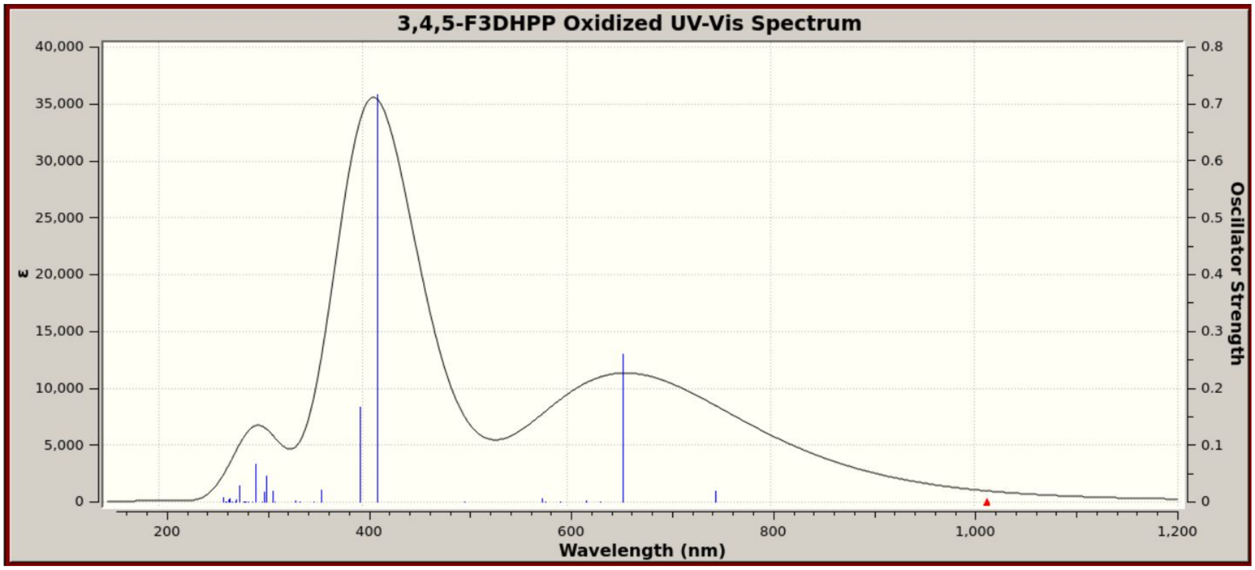

4-OMeDHPP

Neutral:

C	2.06637200	0.55784300	-0.00690500
C	1.00277400	1.45200800	-0.08693200
C	-0.17704900	0.66966300	-0.05619400
C	0.17500900	-0.67052900	0.05676000
C	-2.06845900	-0.55861600	0.00706800
C	-1.00488100	-1.45281000	0.08719200
H	1.10096500	2.52806300	-0.08985000
H	-1.10317300	-2.52885500	0.08970600d
N	-1.56236200	0.75491500	-0.08116000
N	1.56034500	-0.75568200	0.08177100
C	-3.49340600	-0.90053700	-0.08120300
C	-4.00895800	-1.95044000	0.69390100
C	-4.37751300	-0.25609100	-0.97201000
C	-5.34036700	-2.35805000	0.59184200
H	-3.35575700	-2.45300100	1.40173800
C	-5.70559300	-0.64237500	-1.07289500
H	-4.01223700	0.54725200	-1.60343000
C	-6.20010800	-1.69877000	-0.29328800
H	-5.69349100	-3.17394200	1.21245000
H	-6.38193300	-0.14694800	-1.76272900
C	-2.27430500	1.98211700	0.01194600
C	-1.95651000	3.02816100	-0.86109000
C	-3.26404600	2.17827300	0.98295900
C	-2.61784900	4.25149700	-0.75772500
H	-1.19998600	2.87410100	-1.62456900
C	-3.92867500	3.39916100	1.06127500
H	-3.50797100	1.37530600	1.66991500
C	-3.61654400	4.46096800	0.20042800

H	-2.35883500	5.05363000	-1.44517000
H	-4.69942800	3.53268100	1.81719800
C	2.27328700	-1.98229100	-0.01153900
C	3.25980400	-2.17874100	-0.98573000
C	1.95403300	-3.03012200	0.85887200
C	3.92169600	-3.40089700	-1.06817300
H	3.49794000	-1.37754800	-1.67678300
C	2.61261400	-4.25452200	0.75151400
H	1.19314500	-2.87909800	1.61862100
C	3.61311600	-4.46201600	-0.20534000
H	4.68529800	-3.53710600	-1.83084200
H	2.34815300	-5.05988300	1.43308500
C	3.49140400	0.89957800	0.08050300
C	4.00667400	1.94928500	-0.69506300
C	4.37594900	0.25513200	0.97087000
C	5.33821200	2.35667400	-0.59388900
H	3.35313100	2.45184500	-1.40258400
C	5.70415400	0.64121900	1.07090300
H	4.01086700	-0.54800200	1.60266800
C	6.19838800	1.69738400	0.29081300
H	5.69110100	3.17239700	-1.21485100
H	6.38083700	0.14585100	1.76044700
C	-4.32009800	5.79263200	0.32201200
H	-5.39065400	5.66510600	0.51837900
H	-4.21375600	6.38622700	-0.59188700
C	4.35255800	-5.77662700	-0.29449400
H	4.56320100	-6.04869000	-1.33486600
H	3.77720200	-6.59148500	0.15707800
H	5.31749600	-5.72919200	0.22803000
H	-3.90946800	6.38809100	1.14851300
O	7.51944900	2.00022900	0.46744600
O	-7.52100500	-2.00182200	-0.47077100
C	-8.06811300	-3.07155300	0.28124200
H	-8.01421600	-2.87714400	1.36099200
H	-9.11521900	-3.14495500	-0.01882000
H	-7.56125700	-4.02108300	0.06241200
C	8.06633300	3.06962300	-0.28521500
H	7.55984700	4.01933600	-0.06632200
H	9.11364900	3.14285700	0.01415500
H	8.01169400	2.87492300	-1.36487300

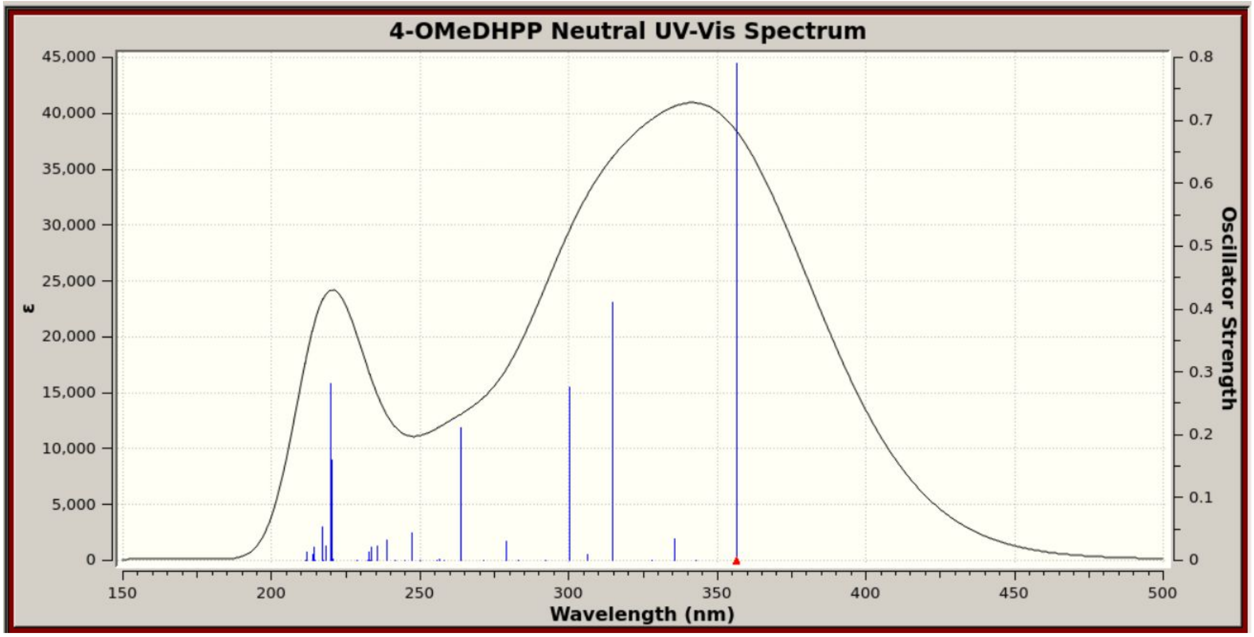

Radical Cation:

C	2.06133300	0.52910700	0.00682100
C	0.97414600	1.44785400	-0.09809500
C	-0.17818500	0.68485900	-0.06230700
C	0.17737400	-0.68481700	0.06388400
C	-2.06216100	-0.52911400	-0.00576900
C	-0.97492000	-1.44783800	0.09932800
H	1.07239900	2.52245100	-0.12622200
H	-1.07310100	-2.52245000	0.12713800
N	-1.55728000	0.77336200	-0.09808600
N	1.55648900	-0.77331100	0.09962500
C	-3.46162400	-0.88510000	-0.07118000
C	-3.88808800	-2.10444700	0.50323400
C	-4.43604600	-0.09443100	-0.73321300
C	-5.20939500	-2.51980600	0.44097700
H	-3.17383800	-2.72387800	1.03619900
C	-5.75052900	-0.50534600	-0.81069200
H	-4.14846400	0.83138300	-1.21563500
C	-6.15857700	-1.71962200	-0.22090100
H	-5.49673200	-3.45376500	0.90869100
H	-6.49380200	0.08849700	-1.33209300
C	-2.26045400	2.02408300	-0.00574800
C	-2.17109300	2.93816200	-1.05702800
C	-2.98992100	2.34118400	1.14323900
C	-2.81876300	4.16972200	-0.95472700
H	-1.61114600	2.68112400	-1.95164700
C	-3.64124700	3.56910800	1.22384500
H	-3.04787300	1.63062000	1.96196700

C	-3.56487300	4.50674000	0.18218900
H	-2.74911600	4.87562000	-1.77806600
H	-4.21309000	3.80747000	2.11695900
C	2.26002400	-2.02382300	0.00684900
C	2.98515700	-2.34243500	-1.14451500
C	2.16846300	-2.93951900	1.05650100
C	3.63095200	-3.57302300	-1.22857300
H	3.03825200	-1.63372800	-1.96517900
C	2.81071700	-4.17363700	0.95083600
H	1.60537500	-2.68453400	1.94974000
C	3.55710800	-4.50996100	-0.18609500
H	4.19386600	-3.81536200	-2.12627500
H	2.73429400	-4.88318100	1.77042700
C	3.46091500	0.88487400	0.07137100
C	3.88748000	2.10360700	-0.50419400
C	4.43532200	0.09446800	0.73366800
C	5.20895900	2.51861200	-0.44281600
H	3.17318100	2.72279200	-1.03738400
C	5.74996800	0.50505700	0.81031000
H	4.14750000	-0.83082300	1.21696900
C	6.15815900	1.71869300	0.21933000
H	5.49640100	3.45208400	-0.91143900
H	6.49328100	-0.08849700	1.33198700
C	-4.25104600	5.84653800	0.29762500
H	-5.24405500	5.74984300	0.74908800
H	-4.36697200	6.32386700	-0.67985900
C	4.27786200	-5.83282400	-0.28461700
H	4.19956000	-6.25661200	-1.29135600
H	3.87620900	-6.56296500	0.42412300
H	5.34689300	-5.71487000	-0.06570100
H	-3.67271500	6.53032700	0.93214800
O	7.46249700	2.01868100	0.34589800
O	-7.46274100	-2.01991300	-0.34824700
C	-7.96031000	-3.23791900	0.21060100
H	-7.82432900	-3.25659300	1.29793500
H	-9.02428100	-3.25367700	-0.02498200
H	-7.47049200	-4.10747500	-0.24210000
C	7.96019500	3.23603100	-0.21424400
H	7.47085000	4.10614600	0.23789900
H	9.02427600	3.25167600	0.02085400
H	7.82374400	3.25379400	-1.30153500

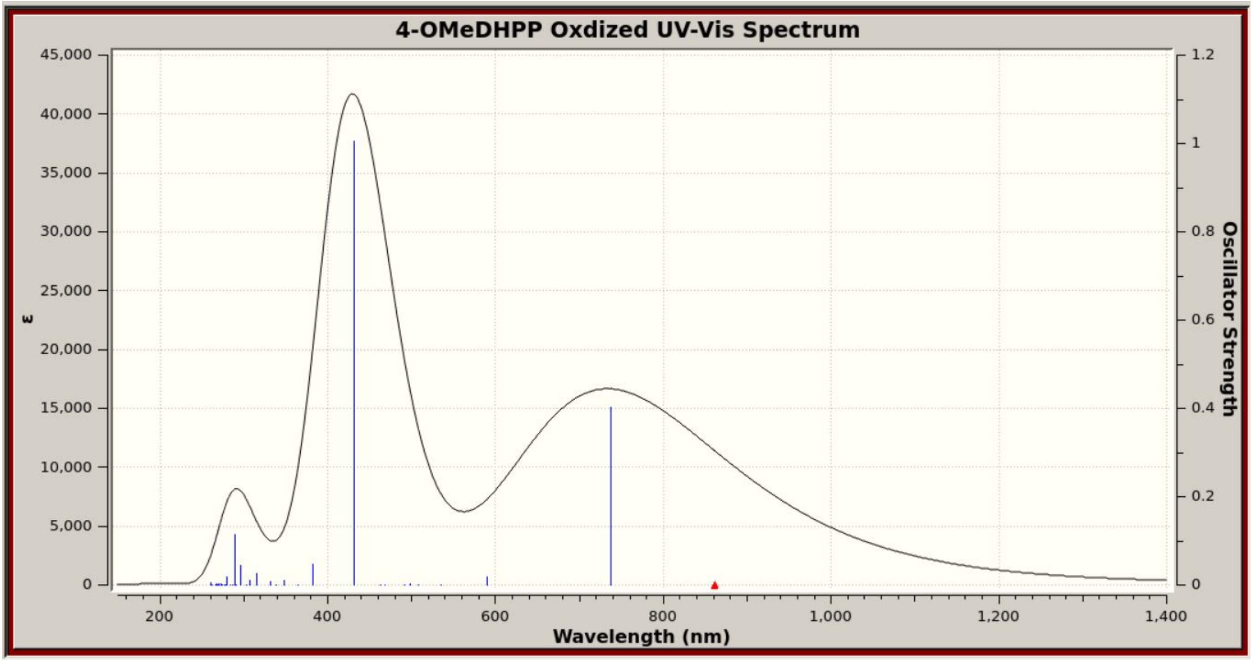

4-SMe-DHPP

Neutral

C	2.10585600	0.34737800	-0.13806200
C	1.12960500	1.33771200	-0.23023700
C	-0.11426000	0.68047400	-0.09249600
C	0.11426100	-0.68048300	0.09250100
C	-2.10585500	-0.34738600	0.13807000
C	-1.12960500	-1.33772000	0.23024600
H	1.32875200	2.39653700	-0.31116000
H	-1.32875100	-2.39654500	0.31117200
N	-1.48285400	0.90099400	-0.06232300
N	1.48285500	-0.90100300	0.06233000
C	2.06597600	-2.19865300	-0.00129800
C	2.93861200	-2.55180600	-1.03725100
C	1.72650900	-3.15119100	0.96470300
C	3.47246000	-3.83671400	-1.08716200
H	3.18891000	-1.82278300	-1.80055700
C	2.25476300	-4.43975900	0.89061100
H	1.05393600	-2.87703800	1.77196800
C	3.14248300	-4.80519500	-0.12832300
H	4.14961000	-4.09601500	-1.89784500
H	1.97738300	-5.17050300	1.64682500
C	-2.06597500	2.19864400	0.00130800
C	-1.72650700	3.15118400	-0.96469100
C	-2.93861300	2.55179400	1.03726000
C	-2.25476300	4.43975200	-0.89059600

H	-1.05393300	2.87703400	-1.77195600
C	-3.47246200	3.83670100	1.08717400
H	-3.18891200	1.82276900	1.80056400
C	-3.14248400	4.80518500	0.12833800
H	-1.97738200	5.17049700	-1.64680800
H	-4.14961300	4.09600000	1.89785600
C	-3.55641200	-0.55828500	0.12865800
C	-4.42395800	0.16042400	-0.71509600
C	-4.11600000	-1.55496200	0.95266700
C	-5.78999800	-0.11043300	-0.73627800
H	-4.02368700	0.92050900	-1.37755400
C	-5.48052200	-1.82370500	0.93323100
H	-3.47001200	-2.10137900	1.63393800
C	-6.33486000	-1.10737900	0.08303700
H	-6.44156500	0.44720000	-1.40246400
H	-5.89220700	-2.58013700	1.59555800
C	3.55641300	0.55827800	-0.12864600
C	4.11600200	1.55495900	-0.95264800
C	4.42395700	-0.16043300	0.71510800
C	5.48052400	1.82370200	-0.93320900
H	3.47001500	2.10137800	-1.63391800
C	5.78999700	0.11042800	0.73629700
H	4.02368700	-0.92052100	1.37756200
C	6.33486000	1.10737500	-0.08301400
H	5.89220800	2.58013400	-1.59553800
H	6.44156200	-0.44720100	1.40248800
S	-8.10388900	-1.41097000	0.07880200
S	8.10389100	1.41095400	-0.07877300
C	-8.18095300	-3.00421500	-0.83040000
H	-7.62488500	-3.78555600	-0.30588400
H	-9.23648700	-3.28726800	-0.87464000
H	-7.79520600	-2.89366900	-1.84704500
C	8.18094700	3.00433500	0.83019000
H	9.23648400	3.28738200	0.87441300
H	7.62490300	3.78560500	0.30554400
H	7.79517400	2.89394600	1.84684300
C	3.74394900	-6.18997600	-0.18470700
H	4.75587800	-6.20308500	0.24178500
H	3.14296500	-6.91124000	0.37854700
H	3.82490900	-6.55027400	-1.21630500
C	-3.74395000	6.18996600	0.18472600
H	-3.14298800	6.91122300	-0.37856100
H	-4.75589600	6.20306700	-0.24172700
H	-3.82487100	6.55027800	1.21632200

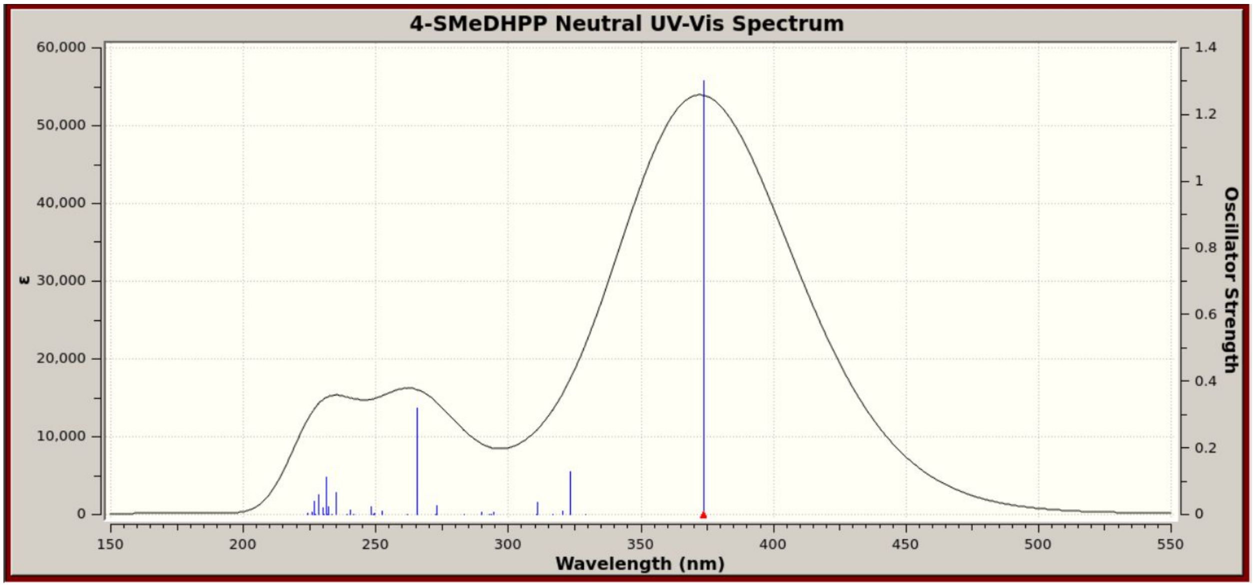

Radical Cation

C	2.10131200	0.33741600	-0.00044500
C	1.10423500	1.35196800	-0.10020100
C	-0.11422900	0.69807500	-0.06126200
C	0.11423000	-0.69807400	0.06126500
C	-2.10131100	-0.33741600	0.00044800
C	-1.10423500	-1.35196700	0.10020700
H	1.30038500	2.41311200	-0.12716800
H	-1.30038600	-2.41311100	0.12717400
N	-1.47888500	0.91369400	-0.09242200
N	1.47888400	-0.91369300	0.09242600
C	2.06372600	-2.22339600	-0.00652000
C	2.75251100	-2.60399300	-1.16136400
C	1.89212100	-3.12990700	1.04117500
C	3.28222400	-3.88851100	-1.25101200
H	2.86749500	-1.90087100	-1.98050800
C	2.41777400	-4.41749800	0.92988200
H	1.35805500	-2.82680400	1.93720100
C	3.12619800	-4.81771200	-0.21071700
H	3.81729800	-4.17893300	-2.15145400
H	2.27922100	-5.11943300	1.74788800
C	-2.06372700	2.22339500	0.00652900
C	-1.89210000	3.12991900	-1.04114700
C	-2.75254400	2.60397700	1.16136400
C	-2.41775900	4.41751100	-0.92984900
H	-1.35802000	2.82682800	-1.93717000
C	-3.28225900	3.88848900	1.25101600
H	-2.86755100	1.90084000	1.98049200
C	-3.12620500	4.81770900	0.21073800
H	-2.27919400	5.11945400	-1.74784500

H	-3.81736000	4.17889600	2.15144800
C	-3.52780900	-0.56369100	-0.06291000
C	-4.42725300	0.31624300	-0.71425500
C	-4.06366500	-1.74312600	0.50461100
C	-5.77581900	0.02649600	-0.78924900
H	-4.05907300	1.21561500	-1.19189900
C	-5.41759700	-2.03012600	0.44192100
H	-3.40904300	-2.43016700	1.03135800
C	-6.30121500	-1.14854000	-0.20831700
H	-6.44017800	0.70963800	-1.31076000
H	-5.78312900	-2.93871400	0.90591500
C	3.52780900	0.56369100	0.06290700
C	4.06366100	1.74312900	-0.50461200
C	4.42726100	-0.31624500	0.71423900
C	5.41759300	2.03013000	-0.44193200
H	3.40903500	2.43017300	-1.03135000
C	5.77582700	-0.02649900	0.78922300
H	4.05908500	-1.21562100	1.19188200
C	6.30121800	1.14854100	0.20829400
H	5.78312100	2.93872100	-0.90592400
H	6.44019000	-0.70964300	1.31072500
S	-8.03678000	-1.39335300	-0.36372000
S	8.03678400	1.39335400	0.36368400
C	-8.34376400	-2.98404100	0.47284300
H	-8.08166500	-2.93640600	1.53271200
H	-9.41906000	-3.15433000	0.38251000
H	-7.81381100	-3.80324800	-0.01933200
C	8.34376000	2.98404500	-0.47287700
H	9.41905700	3.15433400	-0.38255300
H	8.08165200	2.93641200	-1.53274500
H	7.81381100	3.80325000	0.01930400
C	3.72224400	-6.20084200	-0.31484400
H	4.79576000	-6.18401500	-0.08681500
H	3.24905800	-6.89595800	0.38494700
H	3.61340300	-6.60799300	-1.32554400
C	-3.72222400	6.20084700	0.31491200
H	-3.24974200	6.89572400	-0.38558800
H	-4.79596000	6.18388200	0.08794300
H	-3.61242400	6.60838300	1.32535700

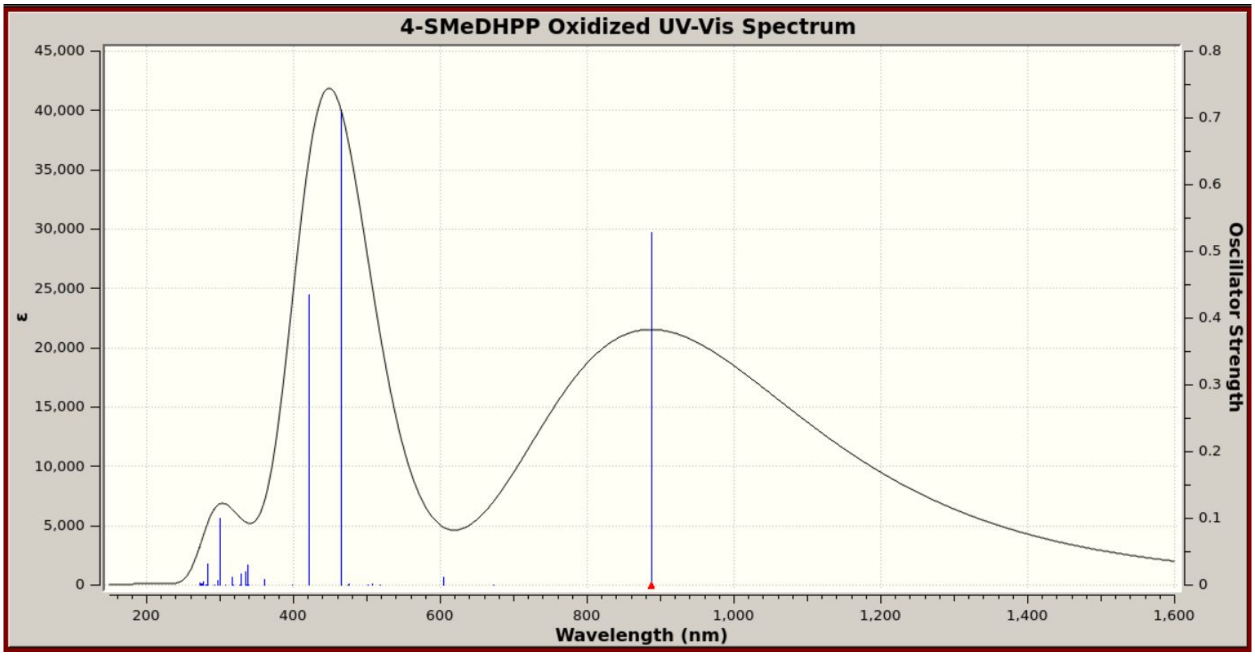

4-tol$_2$ADHPP

Neutral

C	2.13492900	-0.08616200	0.11998200
C	1.29204400	-1.19506400	0.13097100
C	-0.02754300	-0.69174000	0.04663100
C	0.02421500	0.69625100	-0.02879800
C	-2.13829800	0.09051800	-0.10214400
C	-1.29540400	1.19956900	-0.11284400
H	1.62483400	-2.22303100	0.12489700
H	-1.62847500	2.22741200	-0.10561800
N	-1.35874300	-1.08102700	-0.00340800
N	1.35541700	1.08535400	0.02162800
C	1.77066300	2.43588100	0.18489800
C	2.62196500	2.81347400	1.23038800
C	1.28444400	3.41433000	-0.68873700
C	2.98869200	4.14825300	1.38031500
H	2.98533900	2.06234800	1.92333900
C	1.64586500	4.74981400	-0.51434900
H	0.62791600	3.12339300	-1.50321200
C	2.50975600	5.14205300	0.51501700
H	3.65033100	4.42546800	2.19805400
H	1.25456400	5.49838400	-1.19957300
C	-1.77289000	-2.43154500	-0.17042600
C	-1.28807800	-3.41130700	0.70253100
C	-2.62042200	-2.80791700	-1.21937400
C	-1.64711300	-4.74685600	0.52413100

H	-0.63432600	-3.12126500	1.51955900
C	-2.98460600	-4.14296500	-1.37351900
H	-2.98247100	-2.05579100	-1.91195800
C	-2.50715000	-5.13800000	-0.50891300
H	-1.25686000	-5.49641100	1.20888100
H	-3.64271000	-4.41940600	-2.19438600
C	-3.60369800	0.11043400	-0.08273500
C	-4.37027100	-0.76185400	0.71390500
C	-4.29940900	1.07522500	-0.83603800
C	-5.75649100	-0.67158500	0.75829600
H	-3.87261100	-1.50086800	1.33327800
C	-5.68564100	1.16736300	-0.80045700
H	-3.73864100	1.74505900	-1.48198000
C	-6.44044900	0.29326700	-0.00037900
H	-6.31964900	-1.34542400	1.39607700
H	-6.19399500	1.91174900	-1.40499000
C	3.60091200	-0.10614400	0.09680200
C	4.29911200	-1.06809200	0.85126900
C	4.36490100	0.76242400	-0.70640600
C	5.68407200	-1.17261100	0.79691000
H	3.73982900	-1.74585000	1.49000900
C	5.75198800	0.68227500	-0.74248200
H	3.86640100	1.51033400	-1.31424100
C	6.43734400	-0.29145500	0.00358900
H	6.19222800	-1.93268700	1.38174700
H	6.31472600	1.37103100	-1.36448400
C	2.93115000	6.58381900	0.67811100
H	3.90962900	6.76927900	0.21491100
H	2.21443800	7.26631800	0.20966300
H	3.01805500	6.85921800	1.73511200
C	-2.92606400	-6.58001400	-0.67613000
H	-2.20261000	-7.26320600	-0.21913700
H	-3.89903800	-6.77110000	-0.20363300
H	-3.02308800	-6.84943900	-1.73375400
N	-7.85573500	0.38325800	0.04072300
C	8.53802700	-0.20802600	-1.27752400
C	8.04537100	-0.77958000	-2.46085700
C	9.72196000	0.54305400	-1.34041100
C	8.71684000	-0.59393500	-3.66675900
H	7.13115100	-1.36401200	-2.43154100
C	10.39238300	0.70358000	-2.55043700
H	10.11139400	1.00205600	-0.43711200
C	9.90663700	0.14251600	-3.73884400
H	8.31106900	-1.04426300	-4.57027000
H	11.30660700	1.29318000	-2.57245500
C	-8.48738900	1.65748600	0.05075800

C	-9.62709800	1.90020000	-0.73085000
C	-7.98891900	2.69951300	0.84828500
C	-10.24829300	3.14666400	-0.70456000
H	-10.02664000	1.10588800	-1.35353500
C	-8.60974700	3.94583400	0.84931900
H	-7.11531000	2.52556700	1.46863100
C	-9.75146000	4.19793700	0.07674100
H	-11.13311300	3.30753400	-1.31692200
H	-8.20538900	4.73566200	1.47906300
C	-8.64612400	-0.79804700	0.07211200
C	-8.32581200	-1.89905000	-0.73764800
C	-9.77001000	-0.88800900	0.90768900
C	-9.10291700	-3.05376800	-0.69829500
H	-7.46678800	-1.84306100	-1.39880200
C	-10.54842400	-2.04288700	0.92181700
H	-10.03332600	-0.04669200	1.54096200
C	-10.23008400	-3.15265200	0.12831200
H	-8.83469500	-3.89172600	-1.33851600
H	-11.41672600	-2.08490700	1.57609200
N	7.85292500	-0.38227200	-0.04367100
C	8.59098400	-0.65392600	1.14065200
C	9.66703800	-1.55511100	1.12481400
C	8.25974500	-0.03046000	2.35355700
C	10.39057500	-1.81094200	2.28675600
H	9.93029700	-2.05542200	0.19819900
C	8.98082500	-0.31124300	3.51157300
H	7.43114000	0.67003800	2.38466600
C	10.06486900	-1.19874200	3.50434200
H	11.21812400	-2.51633700	2.24784000
H	8.70053400	0.18266800	4.43965200
C	-11.05464600	-4.41752200	0.18054500
H	-11.04846200	-4.94053100	-0.78221200
H	-10.66806500	-5.11964300	0.93208700
H	-12.09724300	-4.20652700	0.44201500
C	10.65136100	0.30368900	-5.04343600
H	11.21839100	1.24074000	-5.06819700
H	11.36923600	-0.51305000	-5.20098500
H	9.96745700	0.30147900	-5.89920500
C	10.86870300	-1.46605500	4.75532100
H	11.28591800	-2.47910900	4.75504400
H	11.71291400	-0.76899800	4.84834100
H	10.25698400	-1.35438600	5.65714200
C	-10.40563400	5.55975500	0.06802700
H	-10.29891700	6.06227400	1.03579000
H	-9.95615200	6.21756500	-0.68857300
H	-11.47497300	5.48986200	-0.15876800

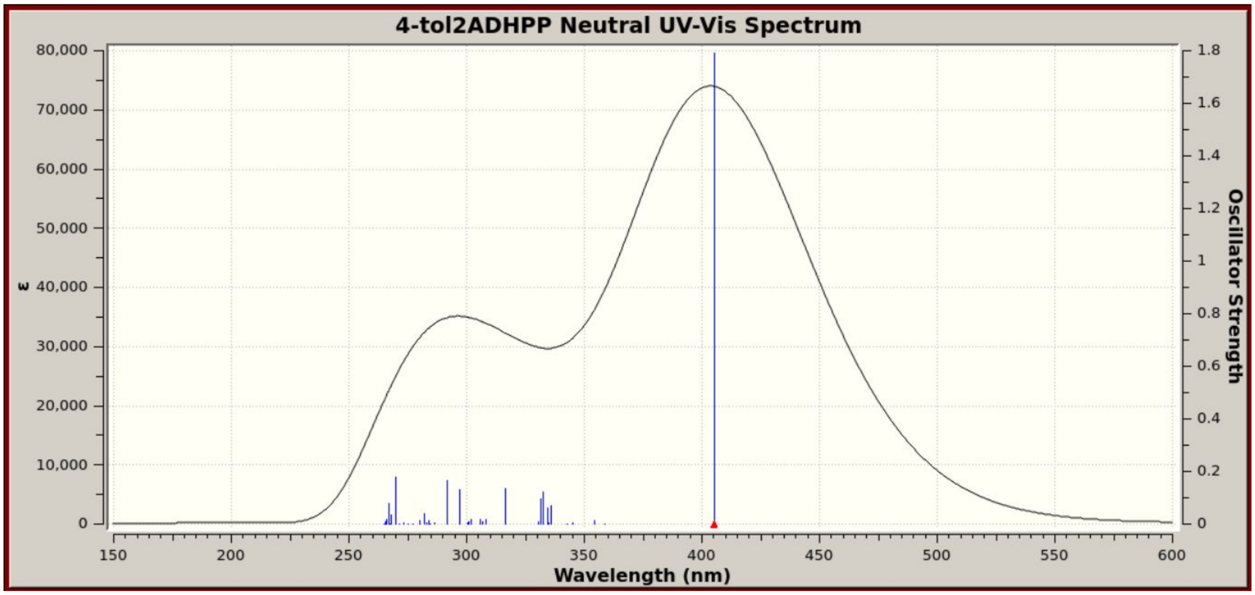

Radical cation

C	2.13007100	-0.05768400	0.09004800
C	1.27538500	-1.19046400	0.14647600
C	-0.02200500	-0.70408400	0.05852400
C	0.02112600	0.70561600	-0.05475200
C	-2.13095500	0.05919500	-0.08644900
C	-1.27627100	1.19198200	-0.14274200
H	1.60899600	-2.21676300	0.17541600
H	-1.60987200	2.21828200	-0.17172300
N	-1.34526100	-1.10114800	0.03304700
N	1.34439000	1.10265900	-0.02937400
C	1.73857800	2.47387400	0.11905000
C	2.35241900	2.91362400	1.29545500
C	1.45273400	3.38294700	-0.90167900
C	2.69162400	4.25705300	1.43355200
H	2.55590900	2.20751500	2.09442000
C	1.78575900	4.72835900	-0.74232600
H	0.97750400	3.03585000	-1.81461800
C	2.41596300	5.18841900	0.42097400
H	3.16747500	4.59149200	2.35207500
H	1.55650200	5.42980700	-1.54039800
C	-1.73930700	-2.47235900	-0.11581800
C	-1.45373600	-3.38165000	0.90475900
C	-2.35271000	-2.91188000	-1.29256800
C	-1.78658000	-4.72708000	0.74493600
H	-0.97886500	-3.03474500	1.81795800
C	-2.69172600	-4.25528200	-1.43113400
H	-2.55601500	-2.20557200	-2.09140400
C	-2.41629400	-5.18690300	-0.41869000

H	-1.55753400	-5.42871000	1.54290600
H	-3.16724300	-4.58952600	-2.34990600
C	-3.57273600	0.08913000	-0.07443500
C	-4.37485000	-0.91295600	0.52588500
C	-4.25134100	1.19910000	-0.63888800
C	-5.75155700	-0.81382100	0.55953900
H	-3.90706400	-1.75889200	1.01456400
C	-5.62618700	1.30439600	-0.61463600
H	-3.68040400	1.97165900	-1.14427500
C	-6.42027500	0.29634900	-0.01268200
H	-6.32879400	-1.58399200	1.05757900
H	-6.10727300	2.15472600	-1.08340400
C	3.57185000	-0.08771400	0.07763800
C	4.25052200	-1.19755900	0.64225500
C	4.37383300	0.91400900	-0.52345800
C	5.62533100	-1.30314700	0.61727700
H	3.67968100	-1.96977400	1.14827900
C	5.75050200	0.81455600	-0.55789700
H	3.90594900	1.75985900	-1.01219500
C	6.41927300	-0.29559800	0.01429100
H	6.10652600	-2.15336200	1.08615200
H	6.32759700	1.58437800	-1.05665600
C	2.80610300	6.63895500	0.57638100
H	3.86290600	6.79268500	0.32188000
H	2.21494900	7.28605900	-0.07873800
H	2.66870800	6.98221000	1.60716800
C	-2.80605600	-6.63746100	-0.57486300
H	-2.21944400	-7.28401900	0.08484000
H	-3.86458600	-6.79044000	-0.32721600
H	-2.66206100	-6.98201400	-1.60433800
N	-7.80473100	0.39583900	0.01796200
C	8.62989900	0.76745500	-0.14810800
C	9.64115700	0.80386700	-1.11698700
C	8.45854600	1.86476200	0.70523100
C	10.46047800	1.92346800	-1.22662000
H	9.77827800	-0.04368700	-1.78124900
C	9.27998800	2.98281200	0.57632200
H	7.68684900	1.83750900	1.46868000
C	10.29821500	3.03410000	-0.38508800
H	11.23719500	1.93805100	-1.98731800
H	9.13530900	3.82602000	1.24728200
C	-8.45854800	1.66769200	-0.07347800
C	-9.49965200	1.85666900	-0.99133600
C	-8.09273100	2.72062600	0.77480300
C	-10.15530800	3.08267100	-1.05768000
H	-9.79341800	1.04125300	-1.64498100

C	-8.75112300	3.94545200	0.68896500
H	-7.30337000	2.57349400	1.50583100
C	-9.79225900	4.15245000	-0.22613600
H	-10.96262200	3.21252300	-1.77425200
H	-8.46058100	4.75053600	1.35941000
C	-8.63016200	-0.76800200	0.14646700
C	-8.45693300	-1.86391500	-0.70843900
C	-9.64794100	-0.80341500	1.10847800
C	-9.28156900	-2.98029700	-0.58633300
H	-7.68610900	-1.83359700	-1.47264900
C	-10.47061400	-1.92128300	1.21107700
H	-9.79259500	0.04655700	1.76801600
C	-10.30207000	-3.03327900	0.37261400
H	-9.13956700	-3.81968400	-1.26263600
H	-11.25694300	-1.93196300	1.96187300
N	7.80366100	-0.39562500	-0.01738800
C	8.45729000	-1.66762800	0.07255800
C	9.50093900	-1.85655500	0.98753400
C	8.08906600	-2.72073000	-0.77451800
C	10.15666000	-3.08261000	1.05232700
H	9.79653100	-1.04104000	1.64022700
C	8.74756300	-3.94559000	-0.69023300
H	7.29773300	-2.57370900	-1.50342900
C	9.79120500	-4.15252500	0.22203800
H	10.96593300	-3.21240300	1.76669800
H	8.45510300	-4.75077100	-1.35972900
C	-11.18215300	-4.25200700	0.51247700
H	-11.18742100	-4.85021900	-0.40403000
H	-10.83284700	-4.90145600	1.32608300
H	-12.21644500	-3.97529000	0.74279400
C	11.21090600	4.23165800	-0.49568900
H	10.75058000	5.12840100	-0.06925600
H	12.15360400	4.06100300	0.04068500
H	11.46783000	4.44474900	-1.53883000
C	10.48909500	-5.48755400	0.32201300
H	11.55627100	-5.36712900	0.53598800
H	10.06386900	-6.09682100	1.13058700
H	10.39153800	-6.06149100	-0.60483500
C	-10.49009800	5.48738300	-0.32774700
H	-10.39358600	6.06193000	0.59882700
H	-10.06404700	6.09612100	-1.13628100
H	-11.55704100	5.36676000	-0.54280400

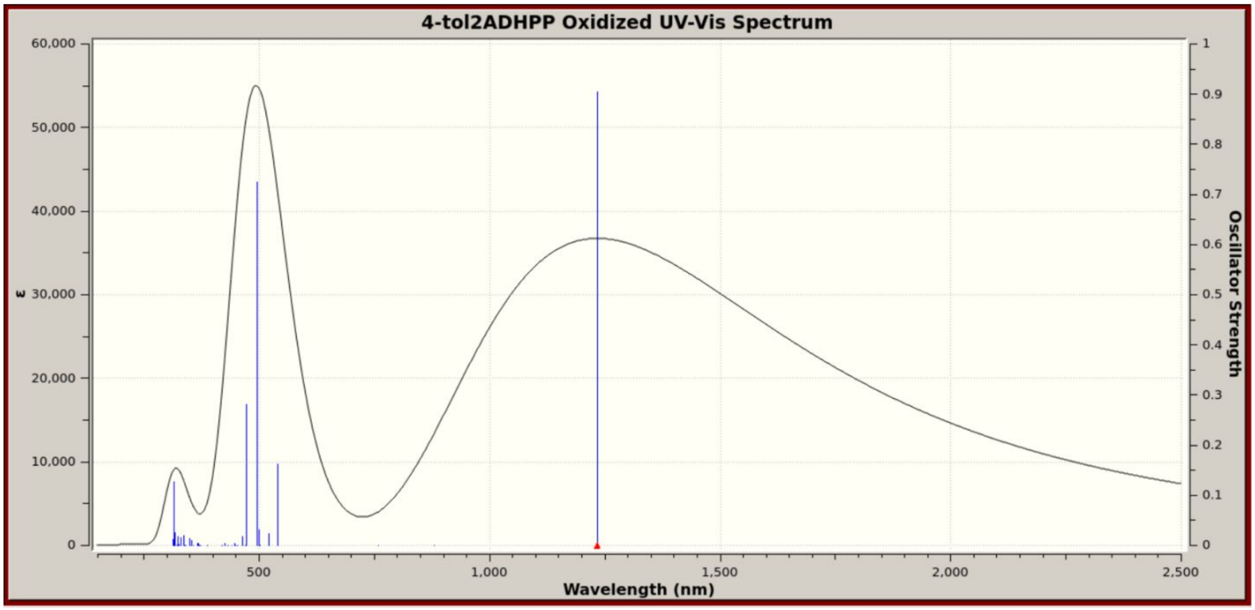

References

(1) Frisch, M. J.; Trucks, G. W.; Schlegel, H. B.; Scuseria, G. E.; Robb, M. A.; Cheeseman, J. R.; Scalmani, G.; Barone, V.; Petersson, G. A.; Nakatsuji, H. Gaussian 16. Gaussian, Inc.: Wallingford CT 2016.

(2) Lindbloom, B. J. brucelindbloom.com http://www.brucelindbloom.com/index.html?ColorCalculator.html (accessed Jan 31, 2023).

(3) Weigert, Frank J., Roberts, J. D. 18C Nuclear Magnetic Resonance Spectroscopy. Determination of Carbon-Fluorine Couplings. *J. Am. Chem. Soc.* **1971**, *93* (10), 2361–2369. https://doi.org/10.1021/ja01969a012.

(4) Nhon, L.; Wilkins, R.; Reynolds, J. R.; Tomlinson, A. Guiding Synthetic Targets of Anodically Coloring Electrochromes through Density Functional Theory. *J. Chem. Phys.* **2021**, *154*, 054110. https://doi.org/10.1063/5.0039511.

(5) Nhon, L.; Tennyson, S. L.; Butt, M. W.; Bacsa, J.; Tomlinson, L.; Reynolds, J. R. Theory-Driven Spectral Control of Bis-EDOT Arylene Radical Cation Chromophores. *Chem.*

Mater. **2022**, *34* (21), 9546–9557. https://doi.org/10.1021/acs.chemmater.2c02054.

(6) Hanson, K.; Roskop, L.; Djurovich, P. I.; Zahariev, F.; Gordon, M. S.; Thompson, M. E. A Paradigm for Blue- or Red-Shifted Absorption of Small Molecules Depending on the Site of π-Extension. *J. Am. Chem. Soc.* **2010**, *132* (45), 16247–16255. https://doi.org/10.1021/ja1075162.

(7) Cardona, C. M.; Li, W.; Kaifer, A. E.; Stockdale, D.; Bazan, G. C. Electrochemical Considerations for Determining Absolute Frontier Orbital Energy Levels of Conjugated Polymers for Solar Cell Applications. *Adv. Mater.* **2011**, *23* (20), 2367–2371. https://doi.org/10.1002/adma.201004554.

(8) Bell, K.-J. J.; Phan, V.; Wagner, E. M.; Hawks, A. M.; Bartlett, K. A.; Collier, G. S. Simple, Tailorable, and Functional Pyrrolopyrrole-Containing Donor-Acceptor Conjugated Polymers. *Macromolecules* **2023**, Under Review.

(9) Christiansen, D. T.; Tomlinson, A. L.; Reynolds, J. R. New Design Paradigm for Color Control in Anodically Coloring Electrochromic Molecules. *J. Am. Chem. Soc.* **2019**, *141*, 3859–3862. https://doi.org/10.1021/jacs.9b01507.

(10) Teran, N. B.; Reynolds, J. R. Discrete Donor-Acceptor Conjugated Systems in Neutral and Oxidized States: Implications toward Molecular Design for High Contrast Electrochromics. *Chem. Mater.* **2017**, *29* (3), 1290–1301. https://doi.org/10.1021/acs.chemmater.6b04725.

(11) Nielsen, C. B.; Angerhofer, A.; Abboud, K. A.; Reynolds, J. R. Discrete Photopatternable π-Conjugated Oligomers for Electrochromic Devices. *J. Am. Chem. Soc.* **2008**, *130* (30), 9734–9746. https://doi.org/10.1021/ja7112273.

(12) Christiansen, D. T.; Wheeler, D. L.; Tomlinson, A. L.; Reynolds, J. R. Electrochromism

of Alkylene-Linked Discrete Chromophore Polymers with Broad Radical Cation Light Absorption. *Polym. Chem.* **2018**, *9* (22), 3055–3066. https://doi.org/10.1039/c8py00385h.
